# HIV seroprevalence in five key populations in Europe: a systematic literature review, 2009 to 2019

**DOI:** 10.2807/1560-7917.ES.2021.26.47.2100044

**Published:** 2021-11-25

**Authors:** Annemarie Rinder Stengaard, Lauren Combs, Virginie Supervie, Sara Croxford, Sarika Desai, Ann K Sullivan, Stine Finne Jakobsen, Quenia Santos, Daniel Simões, Jordi Casabona, Jeffrey V Lazarus, John B F de Wit, Frank M Amort, Anastasia Pharris, Lina Nerlander, Dorthe Raben

**Affiliations:** 1 Centre of Excellence for Health, Immunity and Infections (CHIP), Rigshospitalet, University of Copenhagen, Copenhagen, Denmark; 2 Sorbonne Université, INSERM, Institut Pierre Louis d’Epidémiologie et de Santé Publique, Paris, France; 3 Independent consultant, London, United Kingdom; 4 Directorate of HIV and Sexual Health, Chelsea and Westminster Hospital NHS Foundation Trust, London, United Kingdom; 5 EPIUnit–Instituto de Saúde Pública, Universidade do Porto, Rua das Taipas, n° 135, Porto, Portugal; 6 Grupo de Ativistas em Tratamentos (GAT), Lisboa, Portugal; 7 Centre d'Estudis Epidemiològics sobre les Infeccions de Transmissió Sexual i Sida de Catalunya (CEEISCAT), Barcelona, Spain; 8 Centro de Investigación Biomédica en Red de Epidemiología y Salud Pública (CIBERESP), Barcelona, Spain; 9 Barcelona Institute for Global Health (ISGlobal), Hospital Clínic, University of Barcelona, Barcelona, Spain; 10 Department of Interdisciplinary Social Science, Utrecht University, Utrecht, Netherlands; 11 FH JOANNEUM, University of Applied Sciences, Bad Gleichenberg, Austria; 12 European Centre for Disease Prevention and Control (ECDC), Stockholm, Sweden

**Keywords:** HIV, prevalence, Europe, epidemiology, men who have sex with men, people who inject drugs, prisoners, sex workers, transgender

## Abstract

**Background:**

In Europe, HIV disproportionately affects men who have sex with men (MSM), people who inject drugs (PWID), prisoners, sex workers, and transgender people. Epidemiological data are primarily available from national HIV case surveillance systems that rarely capture information on sex work, gender identity or imprisonment. Surveillance of HIV prevalence in key populations often occurs as independent studies with no established mechanism for collating such information at the European level.

**Aim:**

We assessed HIV prevalence in MSM, PWID, prisoners, sex workers, and transgender people in the 30 European Union/European Economic Area countries and the United Kingdom.

**Methods:**

We conducted a systematic literature review of peer-reviewed studies published during 2009–19, by searching PubMed, Embase and the Cochrane Library. Data are presented in forest plots by country, as simple prevalence or pooled across multiple studies.

**Results:**

Eighty-seven country- and population-specific studies were identified from 23 countries. The highest number of studies, and the largest variation in HIV prevalence, were identified for MSM, ranging from 2.4–29.0% (19 countries) and PWID, from 0.0–59.5% (13 countries). Prevalence ranged from 0.0–15.6% in prisoners (nine countries), 1.1–8.5% in sex workers (five countries) and was 10.9% in transgender people (one country). Individuals belonging to several key population groups had higher prevalence.

**Conclusion:**

This review demonstrates that HIV prevalence is highly diverse across population groups and countries. People belonging to multiple key population groups are particularly vulnerable; however, more studies are needed, particularly for sex workers, transgender people and people with multiple risks.

## Introduction

Men who have sex with men (MSM) and people who inject drugs (PWID) are – and have historically been – disproportionately affected by HIV in Europe as well as globally, which has prompted many European countries to prioritise these groups for HIV prevention, testing, treatment and surveillance activities [1-6]. Thirty-nine percent of new HIV diagnoses reported in the European Union/European Economic Area (EU/EEA) in 2019 were attributed to sex between men. HIV transmission because of injecting drug use accounted for 4% of new diagnoses in the EU/EEA in 2019, but more than a quarter of new diagnoses in two countries and greater than 10% in another three [1]. Historically, PWID have accounted for a much higher proportion (> 50%) of AIDS cases in the mid-1990s in several of the large southern/western European countries [7]. However, other population groups such as prisoners, sex workers, and transgender people, as well as people belonging to several of these key population groups – while smaller in terms of population size and less studied – are also at higher risk for HIV and other sexually transmitted and blood-borne infections because of a range of structural, legal, social, economic, behavioural and biological factors [8-12]. While country-specific estimates of the size of these key population groups vary across the EU/EEA countries and are scarce for some [13], available data suggest that MSM constitute between 0.03–5.6% of the adult (here, 15–64 years) male population [14], PWID comprise 0.34% (range: 0.23–0.47) of the adult (15–64 years) population [5], prisoners make up 0.13% (range: 0.003–0.39) of the total population [15], sex workers constitute ca 0.3% (range: 0.05–0.7) of the adult (15 years or older) female population [16] and transgender people are estimated to be between 0.39–2.7% of the total population [17]. Compared with these groups, the risk of HIV infection in the overall population is very low in the EU/EEA where HIV prevalence has been estimated at 0.2% overall [18], and ranges from less than 0.1% to 0.7% in countries with available data [19,20].

Epidemiological data about the HIV epidemic in Europe are primarily available from national HIV case surveillance systems that collect basic demographic data on people newly diagnosed with HIV, including information on gender, age and probable route of HIV transmission [1]. Probable route of transmission captures data on exposure categories such as injecting drug use and sex between men. However, information on risk factors such as history of sex work or imprisonment and gender identity, e.g. transgender, is not routinely or uniformly collected for surveillance purposes in most countries. As such, notification data from these key population groups are lacking, as is information on people belonging to multiple key population groups, e.g. sex workers who inject drugs. Furthermore, data on new diagnoses do not provide a complete picture of the HIV epidemic since people with undiagnosed HIV are not captured and some countries do not adjust their data to take into account out-migration of people with HIV after they have been diagnosed and registered. Also, accurate population size estimates are often lacking, making it hard to generate prevalence figures from case surveillance data even if robust numerators were available by key population group.

These data limitations restrict our understanding of the epidemiology of HIV in key populations at higher risk of HIV in Europe. Bio-behavioural surveillance studies that assess risk factors for and seroprevalence of HIV in key population groups can help address these shortcomings and provide a broader understanding of the epidemiology of HIV in the EU/EEA. Currently in Europe, such bio-behavioural surveillance occurs in the form of one-off surveys of varying methodological robustness rather than repeated country-wide surveillance. Moreover, there is no established mechanism for collating this type of information at the European level.

To assess the extent to which HIV affects European populations and to generate an additional source of information to better inform HIV prevention and control efforts in Europe, we conducted a systematic literature review of HIV seroprevalence among MSM, PWID, prisoners, sex workers, transgender people, migrants and pregnant women in the 30 EU/EEA countries and the UK. Here we report data for MSM, PWID, prisoners, sex workers and transgender people. Findings for migrants and pregnant women were also collated and are reported elsewhere [21].

## Methods

This review was conducted in accordance with the Preferred Reporting Items for Systematic Reviews and Meta-Analyses (PRISMA) Statement [22].

### Search strategy and selection criteria

Searches for peer-reviewed articles were conducted in PubMed, Embase and the Cochrane Library without any language restrictions on 17–19 June 2019. We used a combination of medical subject headings (MeSH) in PubMed and the Cochrane Library or Emtree terms and keywords in Embase. We included terms for HIV, prevalence, and the names of the relevant key population groups and then applied a geographical search filter to retrieve results for the EU/EEA countries. The detailed search strings are available in the supplement (Table S1a-c). All references retrieved were stored in an EndNote library and duplicates were removed with EndNote version X9 (Thomson Reuters, New York, United States (US)) and manually. 

We included peer-reviewed articles of any study design published between 2009 and 2019 that reported HIV seroprevalence data in adults sampled in 2004 or later (Supplementary Table S2). For the purposes of this study, we considered adults as those aged ≥ 15 years, given that behaviours associated with greater HIV acquisition risk, e.g. sexual contact or injecting drug use, become more prevalent from around that age. Inclusion was restricted to studies with the main purpose of measuring seroprevalence in the included population groups. Studies evaluating HIV testing interventions were excluded as these were often targeted at higher-risk groups within the key populations and frequently did not include people already living with diagnosed HIV when reporting positivity rates. Studies were included if descriptions of HIV testing methods and specimen sampling were available, i.e. studies presenting only self-reported HIV prevalence were excluded. Studies were also excluded if the sample size was fewer than 100 participants, except for studies of transgender people and studies of any population group from countries with a population of 1 million or fewer inhabitants, for which sample sizes of 50 inhabitants or more were accepted. Grey literature and modelling studies were not included. Secondary references from all relevant systematic reviews identified from the search were checked to identify any additional relevant articles not appearing in the original search results.

### Screening and data extraction

Title and abstract screening of all retrieved studies was carried out in pairs, independently of one another and based on the specified inclusion/exclusion criteria (all authors). If the two reviewers disagreed or inclusion could not be determined upon the first screening, then the full text of the article was reviewed. A third reviewer was consulted if consensus could not be reached. Full text articles were retrieved for all included abstracts.

Data extraction was performed simultaneously with the full text review of the articles (ARS, LC, DR, SFJ, AKS, VS, SC, SD). A data extraction protocol specifying all data items to be extracted was developed. Data extraction items included – but were not limited to – the overall study characteristics, study population detail, sampling approach, recruitment setting, laboratory test and HIV prevalence. Each included article was also evaluated for its quality based on a framework that assessed the risk of bias related to the sampling method, sampling venue type/coverage and sample size; for studies of prisoners, risk of bias in terms of gender, age and drug injection was also assessed (Supplementary Table S3). Each criterion was scored with a value between 0 and 2 with higher scores indicating lower risk of bias. Overall (summary) quality scores were calculated for each study (Supplementary Table S4).

To ensure consistency in the data extraction, data were extracted in pairs independently for the first 20 articles and the results were compared. Any differences were discussed within the team and the data extraction protocol was refined accordingly. For the remaining articles, reviewers continued to work in pairs but independently performed full text reviews and provided decisions regarding inclusion/exclusion with reasons for exclusion of papers. One person then performed the main data extraction and the second person performed quality checks. Both reviewers also independently completed the bias assessments and the results were compared at the end of the process. Any discrepancies in the full text review decisions and/or the bias scores were discussed within the pair and a third reviewer was consulted as required to provide input and reach consensus.

The unit for data extraction was ‘study’ and not ‘article’, i.e. if an article reported data from multiple distinct populations, e.g. prevalence data for MSM, PWID and sex workers, or for multiple countries, then each population or country-specific data point was treated as a ‘study’. Inclusion criteria and risk of bias were evaluated at ‘study’ level to the extent permitted by available information. If the same dataset was reported in several articles, then the earliest article reporting on the full study population was retained.

### Data analysis

Data were grouped by key population and presented in forest plots by country with simple or pooled (if two or more studies were identified) prevalence and 95% confidence intervals (CI), built using SAS studio (SAS Institute, Cary, North Carolina, US). If the CI was missing from the extracted data, study-specific 95% CI were calculated using Fisher’s exact test. Studies of lower quality (with summary bias scores of 0) were removed from the forest plots, provided that other studies were available for that given country or population group. If prevalence was reported by city, gender or other population sub-group alone, then overall prevalence was calculated and the study included in the forest plots. If a study reported data for multiple time points, then the most recent data point was used. All prevalence numbers were rounded to one decimal point in text, figures and tables.

## Results

**Figure 1:**
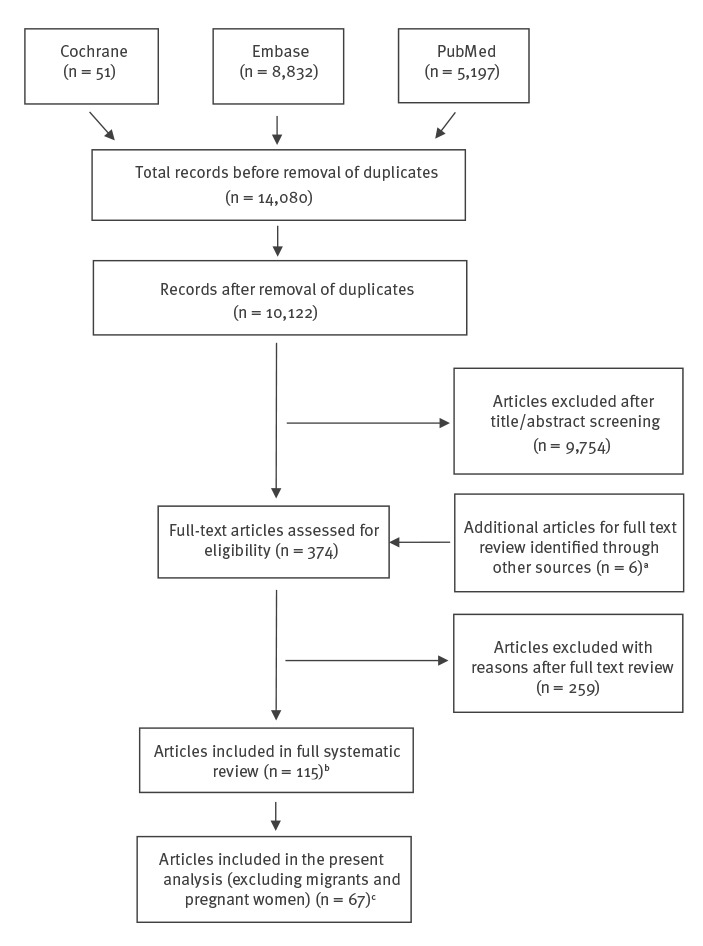
PRISMA flow diagram with results of the systematic literature search and study selection

^a^ Articles identified from screening titles and abstracts of secondary articles identified from reference lists of relevant systematic reviews.

^b^ See [21].

^c^ The 67 articles corresponded to 87 ‘studies’, defined as a prevalence data point relating to a specific country or population group.

The results of our full original literature search covering MSM, PWID, prisoners, sex workers, transgender people, migrants and pregnant women [21] and the selection of studies are outlined in Figure 1. A total of 67 articles reporting HIV seroprevalence in MSM, PWID, prisoners, sex workers and transgender people from 23 EU/EEA countries, including five in languages other than English, were included in this analysis, which corresponded to 87 ‘studies’. The largest number of studies, i.e. data points, were identified for MSM (n = 33) and PWID (n = 30), followed by prisoners (n = 14), sex workers (n = 9) and transgender people (n = 1). Fifteen of the studies included data for people belonging to multiple (overlapping) risk groups (n = 5 for PWID, n = 7 for sex workers and n = 4 for prisoners (Table). The highest number of studies were conducted in Spain (n = 14), the United Kingdom (UK) (n = 11), Italy (n = 8) and Croatia (n = 8). No peer-reviewed published studies that met our inclusion criteria were identified from the following eight EU/EEA countries: Austria, Denmark, Iceland, Ireland, Latvia, Liechtenstein, Malta and Norway.

**Table:** Included HIV prevalence studies for the European Union and European Economic Area and the United Kingdom, by country and population group, 2009–2019 (n = 87 studies)

Country	Number of studies by population group					
	MSM (n = 33^a^)	PWID (n = 30^b,c^)	Prisoners (n = 14^b^)	SW (n = 9^b^)	TG (n = 1)	TOTAL (n = 87)
Belgium	2 [24,29]	0	0	0	0	2
Bulgaria	1 [24]	0	0	0	0	1
Croatia	2 [25,26]	5 [25,39-42]	1 [68]	0	0	8
Cyprus	1 [27]	0	0	0	0	1
Czech Republic	1 [28]	0	0	0	0	1
Estonia	0	2 [64,65]	1 [80]	0	0	3
Finland	0	0	1 [69]	0	0	1
France	2 [34,35]	2 [53,54]	1 [73]	0	0	5
Germany	1 [24]	2 [43,66]	1 [70]	0	0	4
Greece	0	2 [55,56]	0	0	0	2
Hungary	1 [30]	1 [44]	2 [71,72]	0	0	4
Italy	2 [24,28]	1 [57]	3 [74-76]	1 [84]	1 [89]	8
Lithuania	1 [24]	1 [44]	0	0	0	2
Luxembourg	0	1 [45]	0	0	0	1
Netherlands	1 [38]	0	0	3 [81-83]	0	4
Poland	1 [24]	1 [58]	0	0	0	2
Portugal	1 [24]	0	1 [77]	2 [87,88]	0	4
Romania	2 [24,28]	0	0	0	0	2
Slovakia	2 [24,28]	0	0	0	0	2
Slovenia	3 [23,24,28]	0	0	0	0	3
Spain	4 [24,28,36,37]	5 [59-63]	3 [67,78,79]	2 [36,85]	0	14
Sweden	1 [24]	1 [46]	0	0	0	2
United Kingdom	4 [24,31-33]	6 [47-52]	0	1 [86]	0	11
Countries with data^d^	19	13	9	5	1	23

EEA: European Economic Area; EU: European Union; MSM: men who have sex with men; PWID: people who inject drugs; SW: sex workers; TG: transgender people.

^a^ One MSM study [23] was excluded from the plots because of a high risk of bias (summary bias score = 0).

^b^ This number includes studies of people belonging to multiple (overlapping) risk groups (five such studies for PWID [39,47,61,62,66], four for prisoners [68,72,73,79], and seven for sex workers [81,82,84-88]).

^c^ One PWID study [66] was excluded from the forest plots because the original dataset was reported in another study [43]. It was kept in the overview table here and the results description because it reported prevalence data for a different population sub-group (migrant PWID vs non-migrant PWID) which was not reported in the original study [43]. **^d^** No peer-reviewed published studies were identified from eight of the 30 EU/EEA countries and the United Kingdom (Austria, Denmark, Iceland, Ireland, Latvia, Liechtenstein, Malta and Norway).

### Men who have sex with men

**Figure 2:**
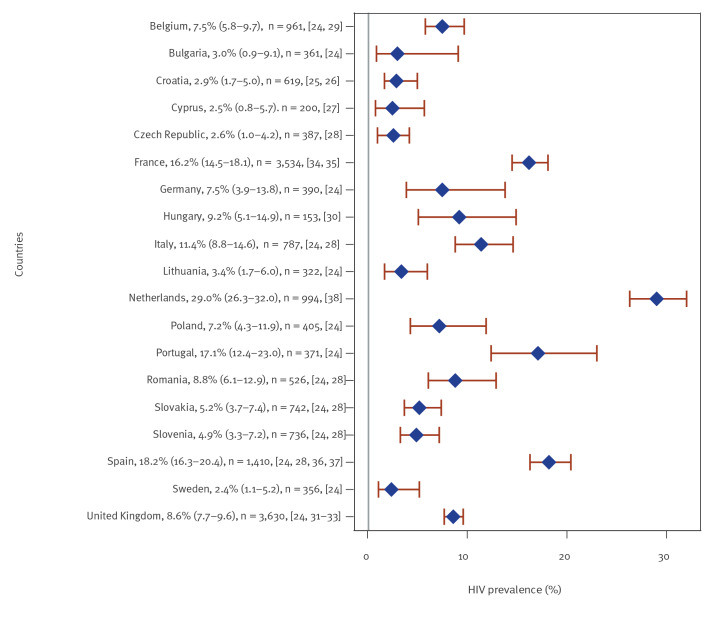
Simple or pooled HIV seroprevalence^a^ among men who have sex with men, by European Union/European Economic Area country and the United Kingdom, 2009–2019 (n = 32 studies)

^a^ Pooled prevalence estimates (combining data from multiple studies) were generated for nine countries with two or more studies available (Belgium, Croatia, France, Italy, Romania, Slovakia, Slovenia, Spain and the United Kingdom).

Of the 33 studies of HIV prevalence among MSM from 19 countries, 32 were included; one [23] study with a summary bias score of 0, i.e. high estimated risk of bias, was excluded (see also Supplementary Table S4). Of the 32 studies included, nearly all studies recruited participants through community-based sampling approaches (time-location, respondent-driven or convenience sampling). Simple or pooled HIV prevalence was < 5% in seven countries (Bulgaria [24], Croatia [25,26], Cyprus [27], the Czech Republic [28], Lithuania [24], Slovenia [24,28] and Sweden [24]), ≥ 5% to < 10% in seven countries (Belgium [24,29], Germany [24], Hungary [30], Poland [24], Romania [24,28], Slovakia [24,28] and the UK [24,31-33]) and highest, ≥ 10% to < 20%, in four western/southern European countries (France [34,35], Italy [24,28], Portugal [24] and Spain [24,28,36,37]) (Figure 2). In one study from the Netherlands [38], conducted in a single low-threshold public sexually transmitted infection (STI) clinic in Amsterdam, prevalence was 29.0%. The majority (n = 30) of the MSM studies were conducted in urban areas, either the capital or another major city, while the remaining three [18,20,28] had national or near-national coverage.

For each country examined, the mean HIV prevalence (blue diamonds), 95% confidence interval (red error bars), total number of participants (‘n’: the size of the study population, either from the individual study or the pooled studies combined) and respective references are given.

### People who inject drugs

**Figure 3:**
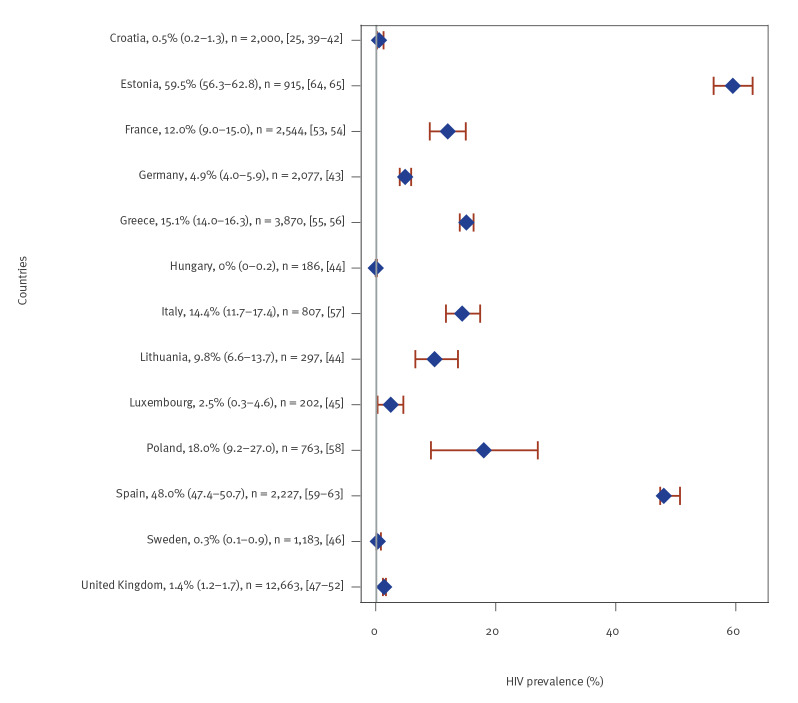
Simple or pooled HIV seroprevalence^a^ among people who inject drugs, by European Union/European Economic Area country and the United Kingdom, 2009–2019 (n = 29 studies)

^a^ Pooled prevalence estimates (combining data from multiple studies) were generated for six countries with 2 or more studies available (Croatia, Estonia, France, Greece, Spain and the United Kingdom).

Of the 30 studies of HIV prevalence among PWID, 29 were included from 13 countries, half of the studies were set in the UK (n = 6), Spain (n = 5) and Croatia (n = 5) (Figure 3). For 22 studies, participants were recruited through harm reduction or drug dependence treatment sites while the remaining studies were primarily based on respondent-driven sampling (RDS). HIV prevalence (simple or pooled) varied greatly and was lowest in the central and northern parts of Europe: < 5% in six countries (Croatia [25,39-42], Germany [43], Hungary [44], Luxembourg [45], Sweden [46] and the UK [47-52]), ≥ 5% to < 10% in Lithuania [44], ≥ 10% to < 20% in four countries (France [53,54], Greece [55,56], Italy [57] and Poland [58]), and 48.0% in Spain [59-63] and 59.5% in Estonia [64,65] (Figure 3). One study [66] was excluded because the original dataset was reported in another study [43] but provided prevalence data for a different population sub-group.

For each country examined, the mean HIV prevalence (blue diamonds), 95% confidence interval (red error bars), total number of participants (‘n’: size of the study population, either from the individual study or the pooled studies combined) and respective references are given.

Among PWID with overlapping risk factors (Supplementary Table S4), prevalence was highest among female PWID who were also sex workers (53.3% vs 33.3% in PWID who were not performing sex work, p < 0.001, in a study from Spain [62]). In a UK-based study, men who have sex with men and inject drugs had an HIV prevalence of 3.2%, which was four times higher than that found among men who have sex with women and inject drugs (0.8%; odds ratio = 4.08; 95% CI: 1.9–8.5) [47]. Migrant PWID from the post-Soviet states tended to have higher prevalence than non-migrant PWID (5.8% vs 4.6%) in a study from eight cities in Germany [66]. Conversely, in a study from Catalonia, Spain, where background HIV prevalence among PWID is one of the highest reported, migrant PWID had significantly lower prevalence than non-migrant PWID (22.4% vs 43.0%; p < 0.001), where migrants were from Europe – both western and eastern Europe – and Africa [61].

### Prisoners

**Figure 4:**
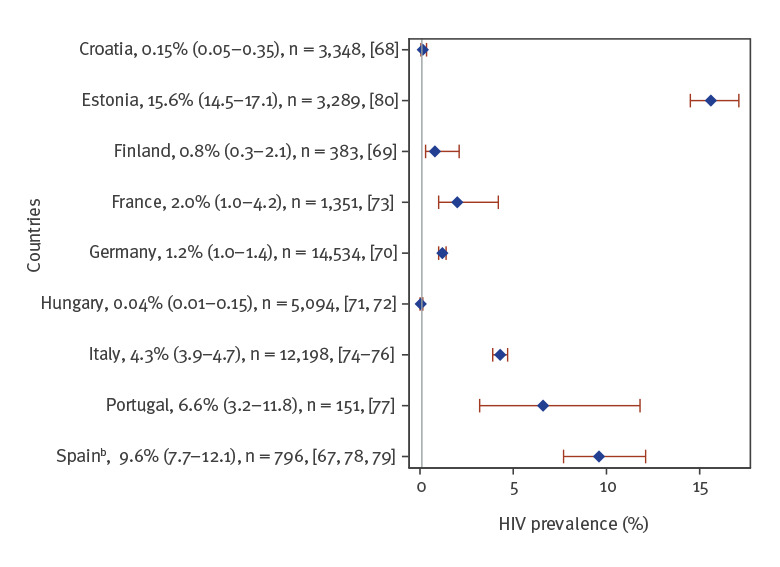
Simple or pooled HIV seroprevalence^a^ among prisoners, by European Union/European Economic Area country and the United Kingdom, 2009–2019 (n = 14 studies)

^a^ Pooled prevalence estimates (combining data from multiple studies) were generated for three countries with two or more studies available (Hungary, Italy and Spain).

^b^ One additional study from Spain [67] reported prevalence of 7.0% but was not included in the pooled prevalence because of missing information about n.

Fourteen studies of HIV prevalence among prisoners were identified from nine countries, with half conducted in three countries: Italy (n = 3), Spain (n = 3) and Hungary (n = 2). One study [67] could not be included in the forest plots because of missing 95% CI and missing information required for CI calculation. Two thirds of the studies on prisoners were conducted in the western part of Europe. Simple or pooled HIV prevalence was < 2% in four countries (Croatia [68], Finland [69], Germany [70] and Hungary [71,72]), ≥ 2% to < 5% in France [73] and Italy [74-76] and ≥ 5% to < 10% in Portugal [77] and Spain [67,78,79], and highest at 15.6% in Estonia [80] (Figure 4).

For each country examined, the mean HIV prevalence (blue diamonds), 95% confidence interval (red error bars), total number of participants (‘n’: size of the study population, either from the individual study or the pooled studies combined) and respective references are given.

Among prisoners belonging to multiple risk groups (Supplementary Table S4. Overview of included HIV seroprevalence studies with key results parameters, by population group and EU/EEA country), prevalence was higher among prisoners who inject drugs compared with those who did not: 39.0% compared with 15.7% in a study from Spain [79], and 0.5% compared with 0.15% in the general prison population in a study from Croatia [68]. Two studies had information about country of origin, but results were inconsistent. One French study found that French-born prisoners had lower prevalence (1.1%) than prisoners from sub-Saharan Africa (15.4%) but not significantly lower prevalence levels than prisoners from North Africa (3.2%) and the Americas (3.5%) [73]. A study from Spain found that HIV prevalence was higher among Spanish-born prisoners compared with those born abroad [79].

### Sex workers

**Figure 5:**
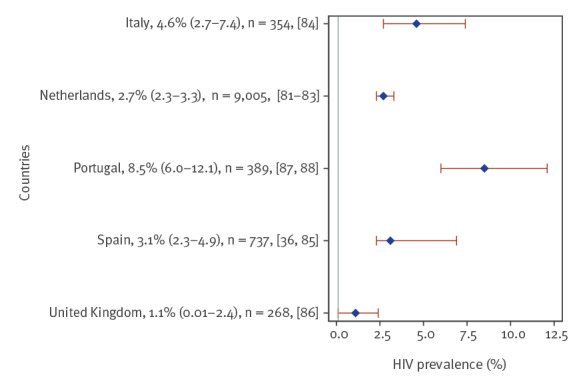
Simple or pooled HIV seroprevalence^a^ among sex workers, by European Union/European Economic Area country and the United Kingdom, 2009–2019 (n = 9 studies)

^a^ Pooled prevalence estimates (combining data from multiple studies) were generated for three countries with 2 or more studies available (the Netherlands, Portugal and Spain).

Nine studies of HIV prevalence among sex workers were included from five countries, the majority from the Netherlands (n = 3), Portugal (n = 2) and Spain (n = 2). Six were based on community-based sampling, of which three were clinic-based and one used a combination of outreach and clinic-based recruitment. Prevalence was < 5% in four countries (the Netherlands [81-83], Italy [84], Spain [36,85] and the UK [86]) and slightly higher at 8.5% in the pooled studies from Portugal [87,88] (Figure 5).

For each country examined, the mean HIV prevalence (blue diamonds), 95% confidence interval (red error bars), total number of participants (‘n’: size of the study population, either from the individual study or the pooled studies combined) and respective references are given.

Among sex workers with multiple risk factors (Supplementary Table S4), prevalence was highest among transgender sex workers (18.8% in the Netherlands [81] and 22.2% in Portugal [87]) and sex workers who inject drugs (13.6% in the Netherlands [81]). In a study of male sex workers, also from the Netherlands, prevalence was 0.1% among heterosexual male sex workers but 3.4% among gay/bisexual male sex workers (p < 0.001) [82]. Migrant sex workers (from eastern Europe, Africa and Latin America) had significantly lower prevalence (0.3%) than non-migrant sex workers (14.7%) in a Spanish study [85]. A study from the UK found that migrant (from eastern Europe) and non-migrant sex workers had similar prevalence levels (1.2% and 0.9%, respectively) [86]. In a Portuguese study [88], documented migrant sex workers tended to have lower prevalence (2.3%) than both non-migrant sex workers (8.0%) and undocumented migrant sex workers (13.6%). The migrant sex workers (documented and undocumented) originated from Latin America (63%), Africa (26%) and Europe (11%).

### Transgender people

Only one study of HIV prevalence among transgender people was identified. This clinic-based study set in Italy reported HIV seroprevalence of 12.1% in 173 people transitioning from male-to-female gender [89]. In addition, as described above, two other studies reported high prevalence among transgender sex workers at 18.8% in the Netherlands [81,87] and 22.2% in Portugal [87].

## Discussion

This review of HIV seroprevalence in the EU/EEA and the UK shows that prevalence is highly diverse across population groups and countries. In terms of populations, our findings suggest that HIV prevalence was highest among MSM and PWID but varied within all groups. However, we also identified more studies for MSM and PWID, exposing a knowledge gap regarding HIV prevalence in the other population groups for a large number of countries, particularly transgender people but also sex workers, for which evidence was confined to two countries in the northern and two in the south-western parts of Europe. Furthermore, we found that individuals with multiple risk factors had higher prevalence than those belonging to one key population group only.

For MSM and PWID, prevalence was highest in the western and southern parts of Europe where the HIV epidemics are older, largely reflecting historical transmission [7], but it was also high in some of the more eastern EU/EEA countries. For MSM, however, incidence appears to have been declining in several western EU/EEA countries, as reflected by a decrease in new HIV diagnoses among MSM in Austria, Belgium, Finland, France, Germany, Greece, Italy, the Netherlands, Portugal and the UK [1]; this trend is likely a result of a combination of prevention interventions, focused and more frequent HIV testing, rapid linkage to care and initiation of antiretroviral therapy (ART), and could potentially lead to reductions in prevalence in the future. Conversely, in the central and eastern EU/EEA, new HIV diagnoses among MSM have increased in recent years in Bulgaria, Cyprus, Poland, Romania and Slovakia, calling for a need to ensure that comprehensive MSM-friendly HIV services are available; this trend suggests that prevalence may also continue to increase in this part of Europe. This review identified relatively few studies of MSM with multiple risks, e.g. foreign-born, PWID or MSM sex workers; however, evidence from France, Belgium and Spain has demonstrated that undiagnosed HIV prevalence appears to be higher in foreign-born MSM compared with non-foreign born MSM [90-92]. For PWID, prevalence varied even more across the EU/EEA, possibly reflecting timing differences in implementation of effective harm reduction programmes, where countries that implemented comprehensive harm reduction interventions early on in the epidemic currently observe lower prevalence levels [50]. Localised outbreaks, such as those occurring in Greece and Romania in 2011–13 [55,93], also contribute to the observed differences in HIV prevalence among PWID and illustrate the importance of maintaining adequate coverage of harm reduction services even in low prevalence settings [94]. Limited, but important, findings related to sex differences indicate that prevalence was higher among female PWID compared with male PWID, suggesting that female PWID may face particular vulnerabilities, including risks of sexual violence, engagement in sex work and dependence on male injecting partners for access to drugs and injecting equipment, as reported elsewhere [95].

Prevalence of HIV and related co-infections is generally higher among prisoners than in the general population in many countries. This is related to high levels of injecting drug use among prisoners, ranging from 2.5% to 37.8% in 15 selected European countries, which represents seven of the nine countries included in this review [96], as well as limited access to harm reduction in some prison settings. HIV prevalence among prisoners was relatively low (< 2%) in about half of identified studies and highest among prisoners who inject drugs, which largely reflects differences in HIV prevalence overall and among PWID in different countries and is consistent with findings reported in a recent global review [10]. Criminalisation of drug use and a related overrepresentation of PWID among prisoners in some countries may also contribute to the observed variation in prevalence.

Prevalence was also relatively low among sex workers who did not belong to other key population groups, ranging from 1.1% to 8.5% overall, and much higher in sex workers who inject drugs and transgender sex workers, ranging from 13.6% to 22.2%. However, data were scarce and covered only five of the 30 EU/EEA countries and the UK. Social, political and cultural contexts such as poverty, risk of violence, criminalisation, discrimination and stigma contribute to increased vulnerabilities among sex workers [11]. Additional structural factors, such as the organisation of sex work and the legal and regulatory policies regarding sex work, may limit sex workers’ ability to negotiate safer sex and access HIV prevention services, and thus further increase the risk of HIV transmission [9].

Data regarding migrant and non-migrant PWID, prisoners and sex workers suggested that that prevalence was higher among foreign-born than non-foreign-born migrants in some countries, and lower in others. This may be explained by differences in levels of injecting drug use in the non-migrant sex workers and prison populations in some countries, a key risk factor for HIV in both populations [10,11]. This may also reflect different background HIV prevalence levels among non-migrant PWID, e.g. high in Spain and low in Germany, and among non-migrant sex workers as well as background prevalence in the migrant’s countries of origin [21]. Of note, when interpreting our results, it should be mentioned that HIV prevalence in a given population does not necessarily reflect the accumulated transmission that has occurred in the country where the prevalence study was conducted since some people living with HIV may have acquired HIV before migrating into that country [97].

We found extremely limited published data on HIV prevalence among transgender people. Although prevalence was high (12.1%) in the single study identified in our review, previous studies from Europe and the United States [12] have reported even higher prevalence levels, particularly among transgender women, with reported pooled prevalence of 19.1% worldwide, 21.6% in five high-income countries, and consistently high infection rates across regions and independent of income level and social, cultural and legal contexts. These findings indicate an urgent need for prevention, testing and care services, as well as additional studies, in this highly vulnerable population group.

Overall, our findings demonstrate that people with overlapping risks are particularly vulnerable – and especially in need of people-centred HIV services. However, more studies are warranted to better understand the sub-group dynamics in the intersections of these high-risk population groups and to inform the design of tailored interventions. There is a continuing need to ensure that key populations at higher risk of HIV have equitable access to HIV services in an environment free of stigma and discrimination, which aim to reduce the risk of infection and onward transmission for these population groups. Evidence-based combination prevention programmes seek to achieve maximum impact on HIV incidence by implementing complementary behavioural, biomedical and structural strategies in the context of a well-researched and understood local epidemiology [98]. Some key elements of combination HIV prevention, include condom provision, pre- and post-exposure prophylaxis (PrEP and PEP), expansion of HIV testing, prompt initiation of antiretroviral treatment (ART) after HIV diagnosis as well as provisions of clean injecting equipment and opioid substitution therapy for PWID [99]. Prison settings present not only challenges but also opportunities for prevention and treatment of HIV and related co-infections. Decriminalisation of drug use and sex work can help the implementation of tailored services and reduce the number of sex workers working in unsafe environments, reducing the risk of HIV transmission both for PWID, sex workers and their partners. Structural measures to reduce social and healthcare related discrimination, marginalisation and violence, alongside other comprehensive social services, can help increase use of HIV and other healthcare services among MSM and transgender people [12,89,100].

Our review has several limitations. Firstly, we only included seroprevalence studies from the published peer-reviewed literature. Hence, unpublished prevalence data or grey literature such as national HIV/STI surveillance reports reporting on prevalence or reports of studies conducted by non-government organisations or other research entities are missing from this review, leaving a gap in the amount of data captured in this analysis. Secondly, sampling methods, recruitment settings and study populations varied greatly across the different studies, impeding the comparability of the data and the methodological robustness of some. Thirdly, a large proportion of studies recruited participants through convenience sampling in a single city or study site and cannot be considered representative of the underlying population at the national level in a given country. Our review includes data sampled in 2004 or later, hence covering a relatively long time period, which may impede direct comparability between older and more recent studies. In terms of the criteria for assessing the risk of bias, the relatively narrow categories for scoring the study sample sizes (100–199; 200+) mean that studies including just over 200 participants received the same risk of bias score as studies covering much larger study samples. Finally, this review covered only studies reporting on measured seroprevalence; many countries now use modelling approaches, which draw on a wide variety of existing surveillance data and serve as an additional robust source of information to assess undiagnosed and total HIV prevalence, overall and in key populations at higher risk.

## Conclusions

This review synthesises previously uncollated evidence on the seroprevalence of HIV across five key population groups in the EU/EEA – MSM, PWID, prisoners, sex workers and transgender people – and also examined combined risk factors and prevalence in people belonging to multiple key population groups. Our results complement other available data sources on the epidemiology of HIV in Europe, particularly HIV case surveillance data published at the European and national level, by providing additional information about HIV prevalence in key populations that are not covered as part of most routine case surveillance data (notably concerning prisoners, sex workers, and transgender people). As such, our findings provide important information for groups who are planning and designing national and local HIV prevention, testing, care and support interventions and serve as a reference for countries with limited or no data. However, the availability of published data varies substantially by country and population group, reflecting both the availability of existing information and the extent to which the data are published in the peer-reviewed literature. In sum, there are limited data and a need to prioritise conducting and publishing HIV seroprevalence studies – particularly among transgender people, but also among male sex workers, female PWID, MSM living outside of capital cities and people with overlapping risks – preferably conducted in a harmonised manner as part of wider national surveillance programmes that include bio-behavioural, modelling and other epidemiologic studies.

## Supplementary Data

495000 Supplement
