# Supplementary material for: HIV seroprevalence in five key populations in Europe: a systematic literature review, 2009 to 2019
Source: Euro Surveill. 2021 Nov 25;26(47):2100044. doi: 10.2807/1560-7917.ES.2021.26.47.2100044 (PMC8619876; doi:10.2807/1560-7917.ES.2021.26.47.2100044)
Supplement: Supplement [file 21-00044_STENGAARD_supplement.pdf]

Supplementary material. HIV seroprevalence in five key populations in Europe: a systematic literature review, 2009 to 2019

This supplementary material is hosted by *Eurosurveillance* as supporting information alongside the article 'HIV seroprevalence in five key populations in Europe: a systematic literature review, 2009 to 2019' on behalf of the authors who remain responsible for the accuracy and appropriateness of the content. The same standards for ethics, copyright, attributions and permissions as for the article apply. Supplements are not edited by Eurosurveillance and the journal is not responsible for the maintenance of any links or email addresses provided therein.

Supplementary Table S1a. Search string and results – PubMed

Search	Terms	Results
#1	Search ("Prevalence"[Mesh] OR "Population Surveillance"[Mesh] OR "Seroepidemiologic Studies"[Mesh] OR "Epidemiological Monitoring"[Mesh] OR prevalen*[TW] OR seroprevalen*[TW] OR sero prevalen*[TW] OR seroepidemiolog*[TW] OR sero epidemiolog*[TW] OR serosurvey*[TW] OR sero survey*[TW] OR seropositiv*[TW] OR sero positiv*[TW] OR serolog*[TI] OR epidemiolog*[TI] OR surveillance*[TI] OR epidemiolog*[OT] OR surveillance*[OT])	1043340
#2	Search ("HIV Infections"[Mesh:NoExp] OR "HIV"[Mesh] OR HIV[TW] OR "Human immune deficiency virus"[TW] OR "Human immune deficiency viruses"[TW] OR Human immunodeficiency virus*[TW] OR Human immuno deficiency virus*[TW])	351509
#3	Search (#1 AND #2)	74441
#4	Search ("HIV Seroprevalence"[Mesh] OR "HIV Seropositivity"[Mesh] OR HIV serodiagnos*[TW] OR HIV serolog*[TW] OR HIV diagnos*[TW] OR HIV screen*[TW] OR ((serolog*[TI] OR serodiagnos*[TI] OR diagnos*[TI] OR screen*[TI] AND (HIV[TI] OR "Human immune deficiency virus"[TI] OR "Human immune deficiency viruses"[TI] OR Human immunodeficiency virus*[TI] OR Human immuno deficiency virus*[TI])) OR ((serolog*[OT] OR serodiagnos*[OT] OR diagnos*[OT] OR screen*[OT] AND (HIV[OT] OR "Human immune deficiency virus"[OT] OR "Human immune deficiency viruses"[OT] OR Human immunodeficiency virus*[OT] OR Human immuno deficiency virus*[OT])))	37222
#5	Search (#3 OR #4)	82315
#6	Search ("Emigrants and Immigrants"[Mesh] OR "Transients and Migrants"[Mesh] OR "person resettlement"[TW] OR "person resettlements"[TW] OR "persons resettlement"[TW] OR "persons resettlements"[TW] OR "people resettlement"[TW] OR "people resettlements"[TW] OR "request for asylum"[TW] OR "request for political asylum"[TW] OR "requested asylum"[TW] OR "requested for political asylum"[TW] OR "requested political asylum"[TW] OR "resettlement of people"[TW] OR "resettlement of person"[TW] OR "resettlement of persons"[TW] OR alien*[TW] OR asylum*[TW] OR displaced*[TW] OR emigrant*[TW] OR emigrat*[TW] OR foreig*[TW] OR immigrant*[TW] OR immigrat*[TW] OR migrant*[TW] OR migrat*[TW] OR minorities[TW] OR minority[TW] OR refugee*[TW] OR born abroad [TW] OR born outside[TW] OR ethnic group [TW] OR ethnicity [TW] OR ((countr*[tiab] OR africa*[tiab] OR asia*[tiab] OR racial*[tiab] OR eastern europe*[tiab] OR endemic area*[tiab] OR foreign*[tiab]) AND (origin*[tiab] OR born*[tiab] OR nationalit*[tiab])) OR origin[TW] OR nationality[TW])	990544

#7	Search ("Prisoners"[Mesh] OR "Prisons"[Mesh] OR cellmate*[TW] OR closed setting*[TW] OR confinement*[TW] OR convict*[TW] OR correctional*[TW] OR custodial*[TW] OR custody[TW] OR custodies[TW] OR detainee*[TW] OR detention*[TW] OR gaol*[TW] OR imprison*[TW] OR incarcerat*[TW] OR inmate*[TW] OR jail*[TW] OR offender[TW] OR offenders[TW] OR penal[TW] OR penitentiar*[TW] OR prison*[TW] OR reformator*[TW] OR remand*[TW])	73347
#8	Search ("Bisexuality"[Mesh] OR "Homosexuality, Male"[Mesh] OR "Sexual and Gender Minorities"[Mesh] OR "male to male sexual contact"[TW] OR "male to male sex"[TW] OR "men having sex with men"[TW] OR "men having sexual relations with men"[TW] OR "men reporting sex with men"[TW] OR "men who had sex with men"[TW] OR "men who have sex with both men and women"[TW] OR "men who have sex with both women and men"[TW] OR "men who have sex with men"[TW] OR "non binary"[TW] OR non heterosexual*[TW] OR bisexual*[TW] OR cruisin'[TW] OR cruising*[TW] OR gay*[TW] OR homosexual*[TW] OR lgbt[TW] OR lesbigay*[TW] OR MASM[TW] OR MSM[TW] OR MSMW[TW] OR GBMSM[TW] OR GB-MSM[TW] OR queer*[TW])	47161
#9	Search ("Transgender Persons"[Mesh] OR "Transsexualism"[Mesh] OR gender reassignment*[TW] OR gender variant*[TW] OR glbt[TW] OR glbtq[TW] OR glbtqq[TW] OR intersex individual*[TW] OR intersex people[TW] OR intersex person*[TW] OR lgbt[TW] OR lgbtq[TW] OR lgbtqq[TW] OR sexual dissident*[TW] OR trans female*[TW] OR trans gender*[TW] OR trans man[TW] OR trans men[TW] OR trans people[TW] OR trans person*[TW] OR trans sexual*[TW] OR trans woman[TW] OR trans women[TW] OR transgender*[TW] OR transman[TW] OR transmen[TW] OR transpeople[TW] OR transperson*[TW] OR transsexual*[TW] OR transwoman[TW] OR transwomen[TW] OR two spirit individual*[TW] OR two spirit people[TW] OR two spirit person*[TW])	9510
#10	Search ("Sex Work"[Mesh] OR "Sex Workers"[Mesh] OR sex work*[TW] OR sex industr*[TW] OR prostitut*[TW] OR exchange sex[TW] OR transactional sex[TW] OR survival sex[TW] OR paid sex[TW] OR "sex for payment"[TW] OR female sex work*[TW] OR fsw[TW] OR male sex work*[TW] OR msw[TW] OR whore*[TW] OR call girl*[TW] OR streetwalker*[TW] OR nightwalker*[TW] OR "lady of the night"[TW] OR "ladies of the night"[TW] OR "walk the pavement"[TW] OR csw)	14712
#11	Search ("Gravidity"[Mesh] OR "Pregnancy"[Mesh] OR "Pregnant Women"[Mesh] OR "Prenatal Care"[Mesh] OR "Prenatal Diagnosis"[Mesh] OR antenatal*[TW] OR ante natal*[TW] OR expectant[TW] OR expecting[TW] OR gravid*[TW] OR pregnanc*[TW] OR pregnant*[TW])	990897
#12	Search ("Substance Abuse, Intravenous"[Mesh] OR "people who inject drugs"[TW] OR "person who inject drugs"[TW] OR "persons who inject drugs"[TW] OR intravenous substance abus*[TW] OR parenteral drug abus*[TW] OR intravenous drug abus*[TW] OR intravenous substance use*[TW] OR intravenous substance misus*[TW] OR parenteral drug use*[TW] OR intravenous drug use*[TW] OR injecting drug use*[TW] OR pwid[TW] OR idu[TW] OR ivdu[TW] OR ((inject*[TI] OR intraven*[TI] OR parenteral[TI] OR use[TI] OR misus*[TI] OR use*[TI]) AND (drug*[TI] OR substance*[TI])) OR ((inject*[OT] OR intraven*[OT] OR parenteral[OT]) AND (drug*[OT] OR substance*[OT])))	33560
#13	Search (#6 OR #7 OR #8 OR #9 OR #10 OR #11 OR #12)	2087848
#14	Search (Europe*[ad] OR Europa*[ad] OR EU[ad] OR EEA[ad] OR EFTA[ad] OR "EU/EEA"[ad] OR "EU/EFTA"[ad] OR ECSC[ad] OR Euratom[ad] OR Eurozone[ad] OR EEC[ad] OR ec[ad] OR (Schengen[ad] AND (area[ad] OR countr*[ad] OR region*[ad]	78623

	OR state[ad] OR states[ad])) OR Euroregion[ad] OR Euroregions[ad] OR Balkan[ad] OR Balkans[ad] OR Baltic[ad] OR (Mediterranean[ad] AND (area[ad] OR countr*[ad] OR region*[ad] OR state[ad] OR states[ad])) OR (Alpine[ad] AND (area[ad] OR countr*[ad] OR region*[ad] OR state[ad] OR states[ad])) OR Scandinavia[ad] OR Scandinavian[ad] OR "Nordic country"[ad] OR "Nordic countries"[ad] OR "Nordic state"[ad] OR "Nordic states"[ad] OR Danubian[ad] OR "Iberian peninsula"[ad] OR "Peninsula iberica"[ad] OR "Peninsule Iberique"[ad] OR "Iberiar Penintsula"[ad] OR Iberia[ad] OR Anatolia[ad] OR Anadolu[ad] OR Anatole[ad] OR Anatolian[ad] OR Yugoslavia[ad] OR Czechoslovakia[ad] OR "Czecho Slovakia"[ad] OR Ceskoslovensko[ad] OR "Cesko slovensko"[ad] OR Benelux[ad] OR Fennoscandia[ad] OR "Fenno Scandinavia"[ad] OR Fennoskandi*[ad] OR (Visegrad[ad] AND (Group[ad] OR Four[ad] OR Triangle[ad])) OR "Visegradska ctyrka"[ad] OR "Visegradska skupina"[ad] OR "Visegradi Egyuttmukodes"[ad] OR "Visegradi negyek"[ad] OR "Grupa Wyszehradzka"[ad] OR "Vysehradska skupina"[ad] OR "Vysehradska stvorka"[ad])	
#15	Search (GB[ad] OR "United kingdom"[ad] OR UK[ad] OR Britain[ad] OR British[ad] OR England[ad] OR English[ad] OR Scotland[ad] OR Scottish[ad] OR Scots[ad] OR Wales[ad] OR Cymru[ad] OR Welsh[ad] OR "North Ireland"[ad] OR "Northern Ireland"[ad] OR Irish[ad] OR Avon[ad] OR Bedfordshire[ad] OR Berkshire[ad] OR Bristol[ad] OR Buckinghamshire[ad] OR Cambridgeshire[ad] OR "Isle of Ely"[ad] OR Cheshire[ad] OR Cleveland[ad] OR Cornwall[ad] OR Cumberland[ad] OR Cumbria[ad] OR Derbyshire[ad] OR Devon[ad] OR Dorset[ad] OR Durham[ad] OR Essex[ad] OR Gloucestershire[ad] OR Hampshire[ad] OR Southampton[ad] OR (Hereford[ad] AND Worcester[ad]) OR Hertfordshire[ad] OR Herefordshire[ad] OR Humberside[ad] OR Huntingdon[ad] OR Huntingdonshire[ad] OR "Isle of Wight"[ad] OR Kent[ad] OR Lancashire[ad] OR Leicestershire[ad] OR Lincolnshire[ad] OR London[ad] OR Manchester[ad] OR Merseyside[ad] OR Middlesex[ad] OR Norfolk[ad] OR Northamptonshire[ad] OR Northumberland[ad] OR Nottinghamshire[ad] OR Oxfordshire[ad] OR Peterborough[ad] OR Rutland[ad] OR Shropshire[ad] OR Salop[ad] OR Somerset[ad] OR Yorkshire[ad] OR Staffordshire[ad] OR Suffolk[ad] OR Surrey[ad] OR Sussex[ad] OR (Tyne[ad] AND Wear[ad]) OR Warwickshire[ad] OR midlands[ad] OR Westmorland[ad] OR Wiltshire[ad] OR Worcestershire[ad] OR "Isle of Man"[ad] OR Jersey[ad] OR Guernsey[ad] OR "Channel Islands"[ad] OR Aberdeen[ad] OR Aberdeenshire[ad] OR Angus[ad] OR Forfarshire[ad] OR Argyll[ad] OR Ayrshire[ad] OR Banffshire[ad] OR Berwickshire[ad] OR bute[ad] OR Caithness[ad] OR Clackmannanshire[ad] OR Cromartyshire[ad] OR Dumfriesshire[ad] OR Dunbartonshire[ad] OR Dumbarton[ad] OR Dundee[ad] OR Lothian[ad] OR Haddingtonshire[ad] OR Edinburgh[ad] OR Fife[ad] OR Glasgow[ad] OR Inverness-shire[ad] OR Kincardineshire[ad] OR Kinross-shire[ad] OR Kirkcudbrightshire[ad] OR Lanarkshire[ad] OR Midlothian[ad] OR Moray[ad] OR Elginshire[ad] OR Nairnshire[ad] OR Orkney[ad] OR Peeblesshire[ad] OR Perthshire[ad] OR Renfrewshire[ad] OR (Ross[ad] AND Cromarty[ad]) OR Ross-shire[ad] OR Roxburghshire[ad] OR Selkirkshire[ad] OR Shetland[ad] OR Zetland[ad] OR Stirlingshire[ad] OR Sutherland[ad] OR Linlithgowshire[ad] OR Wigtownshire[ad] OR Anglesey[ad] OR Brecknockshire[ad] OR Caernarfonshire[ad] OR Carmarthenshire[ad] OR Cardiganshire[ad] OR Ceredigion[ad] OR Clwyd[ad] OR Denbighshire[ad] OR Dyfed[ad] OR Flintshire[ad] OR Glamorgan[ad] OR Gwent[ad] OR Gwynedd[ad] OR Merionethshire[ad] OR Montgomeryshire[ad] OR Monmouthshire[ad] OR Pembrokeshire[ad] OR Powys[ad] OR Radnorshire[ad] OR Antrim[ad] OR Aontroim[ad] OR "Contae Aontroma"[ad] OR Anthrim[ad] OR Antrim[ad] OR Entrim[ad] OR Armagh[ad] OR "Ard Mhacha"[ad] OR Airmagh[ad] OR Belfast[ad] OR (Down[ad] AND (district[ad] OR council[ad] OR County[ad])) OR "An Dun"[ad] OR "an Duin"[ad] OR Doon[ad] OR Doun[ad] OR Fermanagh[ad] OR "Fear Manach"[ad] OR "Fhear Manach"[ad] OR Fermanay[ad] OR Londonderry[ad] OR Doire[ad] OR Dhoire[ad] OR Lunnonderrie[ad] OR Derry[ad] OR Birmingham[ad] OR Leeds[ad] OR Sheffield[ad] OR	6858089

	Bradford[ad] OR Liverpool[ad]) OR (Sweden[ad] OR Sverige[ad] OR Swedish[ad] OR Svenska[ad] OR svenskar[ad] OR Swede[ad] OR Swedes[ad] OR Norrland[ad] OR Mellansverige[ad] OR Smaland[ad] OR Stockholm*[ad] OR Sydsverige[ad] OR Vastsverige[ad] OR Blekinge[ad] OR Dalarna[ad] OR Gavleborg*[ad] OR Gotland*[ad] OR Halland*[ad] OR Jamtland*[ad] OR Jonkoping*[ad] OR Kalmar[ad] OR Kronoberg*[ad] OR Norrbotten*[ad] OR Orebro[ad] OR Ostergotland*[ad] OR Skane[ad] OR Sodermanlands[ad] OR Uppsala[ad] OR Varmland*[ad] OR Vasterbotten*[ad] OR Vasternorrland*[ad] OR Vastmanland*[ad] OR vastergotland*[ad] OR Gotaland*[ad] OR Gothenburg[ad] OR Goteborg[ad] OR Malmo[ad] OR Vasteras[ad] OR Linkoping[ad] OR Helsingborg[ad] OR Halsingborg[ad] OR Norrkoping[ad]) OR (Spain[ad] OR Espana[ad] OR Spanish[ad] OR Espanol*[ad] OR Spaniard*[ad] OR Andalucia[ad] OR Andalusia[ad] OR Aragon[ad] OR Arago[ad] OR Cantabria[ad] OR Canarias[ad] OR "Canary Islands"[ad] OR (Canaries[ad] AND island*[ad]) OR "Castile and leon"[ad] OR "Castilla y Leon"[ad] OR "Castile La Mancha"[ad] OR "Castilla La Mancha"[ad] OR Cataluna[ad] OR Catalonia[ad] OR Ceuta[ad] OR Madrid[ad] OR Melilla[ad] OR Navarra[ad] OR Navarre[ad] OR Valencia*[ad] OR Extremadura[ad] OR Galicia[ad] OR Balears[ad] OR "Balearic Islands"[ad] OR "Balear Islands"[ad] OR Baleares[ad] OR "La Rioja"[ad] OR "Pais Vasco"[ad] OR "Basque Country"[ad] OR "Baske region"[ad] OR Euskadi[ad] OR Asturias[ad] OR Murcia[ad] OR Coruna[ad] OR Alava[ad] OR Araba[ad] OR Albacete[ad] OR Alicante[ad] OR Alacant[ad] OR Almeria[ad] OR Avila[ad] OR Badajoz[ad] OR Badajos[ad] OR Barcelona[ad] OR Burgos[ad] OR Caceres[ad] OR Cadiz[ad] OR Castellon[ad] OR Castello[ad] OR "Ciudad Real"[ad] OR Cordoba[ad] OR Cuenca[ad] OR Eivissa[ad] OR Ibiza[ad] OR Formentera[ad] OR "El Hierro"[ad] OR Fuerteventura[ad] OR Galiza[ad] OR Girona[ad] OR Gerona[ad] OR "Gran Canaria"[ad] OR Granada[ad] OR Guadalajara[ad] OR Guipuzcoa[ad] OR Gipuzkoa[ad] OR Huelva[ad] OR Huesca[ad] OR Jaen[ad] OR "La Gomera"[ad] OR "La Palma"[ad] OR Lanzarote[ad] OR Leon[ad] OR Lleida[ad] OR Lerida[ad] OR Lugo[ad] OR Malaga[ad] OR Mallorca[ad] OR Majorca[ad] OR Menorca[ad] OR Minorca[ad] OR Murcia[ad] OR Ourense[ad] OR Orense[ad] OR Palencia[ad] OR Pontevedra[ad] OR Salamanca[ad] OR Segovia[ad] OR Sevilla[ad] OR Seville[ad] OR Soria[ad] OR Tarragona[ad] OR Tenerife[ad] OR Teruel[ad] OR Toledo[ad] OR Valladolid[ad] OR Vizcaya[ad] OR Biscay[ad] OR Zamora[ad] OR Zaragoza[ad] OR Saragossa[ad] OR "Las Palmas"[ad] OR Bilbao[ad] OR Bilbo[ad]) OR (Slovenia*[ad] OR Slovenija[ad] OR slovensk*[ad] OR Slovenci[ad] OR Slovene*[ad] OR Gorenjska[ad] OR Carniola[ad] OR Goriska[ad] OR Gorizia[ad] OR Jugovzhodna[ad] OR Koroska[ad] OR Carinthia[ad] OR "Notranjsko kraska"[ad] OR "Obalno kraska"[ad] OR "Coastal karst"[ad] OR Osrednjeslovenska[ad] OR Podravska[ad] OR Drava[ad] OR Pomurska[ad] OR Mura[ad] OR Savinjska[ad] OR Savinja[ad] OR Spodnjeposavska[ad] OR Zasavska[ad] OR "Central Sava"[ad] OR Posavska[ad] OR "Lower Sava"[ad] OR Ljubljana[ad] OR Laibach[ad] OR Lubiana[ad] OR Maribor[ad] OR "Marburg an der Drau"[ad] OR Kranj[ad] OR Carnium[ad] OR Creina[ad] OR Chreina[ad] OR Krainbur[ad] OR Koper[ad] OR Capodistria[ad] OR Kopar[ad] OR Celje[ad] OR "Novo mesto"[ad] OR Neustadtl[ad] OR Domzale[ad] OR Velenje[ad] OR Wollan[ad] OR Woellan[ad] OR "Nova Gorica"[ad] OR Kamnik[ad]) OR (Slovakia[ad] OR Slovensk*[ad] OR Slovak*[ad] OR Slovaci[ad] OR Slovenki[ad] OR Bratislav*[ad] OR Presporok[ad] OR Pressburg[ad] OR Pressburg[ad] OR Posonium[ad] OR Banskobystri*[ad] OR "Banska Bystrica"[ad] OR Neusohl[ad] OR Besztercebanya[ad] OR Kosic*[ad] OR Kaschau[ad] OR Kassa[ad] OR Nitrian*[ad] OR Nitra[ad] OR Neutra[ad] OR Nyitra[ad] OR Nyitria[ad] OR Trnav*[ad] OR Tyrnau[ad] OR Nagyszombat[ad] OR Tyrnavia[ad] OR Presov*[ad] OR Trencian*[ad] OR Trencin[ad] OR Trentschin[ad] OR Trencsen[ad] OR Zilina[ad] OR Sillein[ad] OR Zsolna[ad] OR Zylina[ad] OR (Martin[ad] AND (city[ad] OR Svaty[ad])) OR Turocszentmarton[ad] OR Poprad[ad] OR Deutschendorf[ad] OR Zvolen[ad]) OR (Romania*[ad] OR Rumania*[ad] OR Roumania*[ad] OR Romani[ad] OR Rumani[ad] OR Alba[ad] OR Arad[ad] OR Arges[ad] OR Bacau[ad] OR Bihor[ad] OR "Bistrita Nasaud"[ad] OR Botosani[ad] OR Braila[ad] OR Brasov[ad] OR Kronstadt[ad] OR Brasso[ad] OR Brassovia[ad] OR Coron[ad] OR Bucharest[ad]	

	OR Bucuresti[ad] OR Buzau[ad] OR Calarasi[ad] OR "Caras-Severin"[ad] OR Cluj[ad] OR Klausenburg[ad] OR Kolozsvar[ad] OR Constanta[ad] OR Tomis[ad] OR Konstantia[ad] OR Kostence[ad] OR Covasna[ad] OR Dambovita[ad] OR Dolj[ad] OR Galati[ad] OR Galatz[ad] OR Galac[ad] OR Kalas[ad] OR Giurgiu[ad] OR Gorj[ad] OR Harghita[ad] OR Hunedoara[ad] OR Ialomita[ad] OR Iasi[ad] OR Jassy[ad] OR Lassy[ad] OR Ilfov[ad] OR Maramures[ad] OR Mehedinti[ad] OR Mures[ad] OR Neamt[ad] OR (Olt[ad] AND (river[ad] OR county[ad] OR region[ad] OR judetul[ad] OR Raul[ad])) OR Prahova[ad] OR Salaj[ad] OR "Satu Mare"[ad] OR Sibiu[ad] OR Suceava[ad] OR Teleorman[ad] OR Timis[ad] OR Tulcea[ad] OR Valcea[ad] OR Vilcea[ad] OR Vaslui[ad] OR Vrancea[ad] OR Timisoara[ad] OR Temeswar[ad] OR Temeschburg[ad] OR Temeschwar[ad] OR Temesvar[ad] OR Temisvar[ad] OR Timisvar[ad] OR Temesva[ad] OR Craiova[ad] OR Ploiesti[ad] OR Ploesti[ad] OR Oradea[ad] OR Varad[ad] OR Varat[ad]) OR (Portugal[ad] OR Portugues*[ad] OR Azores[ad] OR Acores[ad] OR Madeira[ad] OR Alentejo[ad] OR Algarve[ad] OR Lisboa[ad] OR Lisbon[ad] OR "Alto Tras-os-Montes"[ad] OR (Ave[ad] AND (community[ad] OR intermunicipal[ad] OR comunidade[ad])) OR Mondego[ad] OR Vouga[ad] OR Beira[ad] OR Cavado[ad] OR Lafoes[ad] OR Douro[ad] OR Porto[ad] OR Oporto[ad] OR Tejo[ad] OR Minho[ad] OR Setubal[ad] OR Pinhal[ad] OR "Serra da Estrela"[ad] OR Tamega[ad] OR Leira[ad] OR Santarem[ad] OR Beja[ad] OR Faro[ad] OR Evora[ad] OR Portalegre[ad] OR "Castelo Branco"[ad] OR Guarda[ad] OR Cimbra[ad] OR Aveiro[ad] OR Viseu[ad] OR Braganca[ad] OR Braganza[ad] OR Braga[ad] OR "Vila real"[ad] OR "Viana do Castelo"[ad] OR Gaia[ad] OR Amadora[ad] OR Funchal[ad] OR Coimbra[ad] OR Almada[ad] OR (Agualva[ad] AND Cacem[ad])) OR (Poland[ad] OR Polska[ad] OR Polish[ad] OR Pole[ad] OR Poles[ad] OR Polski[ad] OR Polak[ad] OR Polka[ad] OR Polacy[ad] OR Dolnoslaskie[ad] OR Silesia*[ad] OR Slask[ad] OR Pomorskie[ad] OR Pomerania*[ad] OR Kujawsko[ad] OR Kuyavian[ad] OR Lodzkie[ad] OR Lodz[ad] OR Lubelskie[ad] OR Lublin[ad] OR Lubuskie[ad] OR Lubusz[ad] OR Lubus[ad] OR Malopolskie[ad] OR Mazowieckie[ad] OR Mazowske[ad] OR Masovia[ad] OR Masovian[ad] OR Opolskie[ad] OR Opole[ad] OR Podkarpackie[ad] OR Subcarpathian*[ad] OR Podlaskie[ad] OR Podlachia[ad] OR Podlasie[ad] OR Slaskie[ad] OR Swietokrzyskie[ad] OR "Varmia Mazuria"[ad] OR "Varmian Mazurian"[ad] OR "Varmia Masuria"[ad] OR "Varmian Masurian"[ad] OR "Warmia Mazury"[ad] OR "Warminsko Mazurskie"[ad] OR "Warmian Masurian"[ad] OR Wielkopolskie[ad] OR Zachodniopomorskie[ad] OR Warsaw[ad] OR Warszawa[ad] OR Krakow[ad] OR Cracow[ad] OR Wroclaw[ad] OR Poznan[ad] OR Gdansk[ad] OR Szczecin[ad] OR Bydgoszcz[ad] OR Katowice[ad]) OR (Netherlands[ad] OR Nederland*[ad] OR Dutch*[ad] OR Drenthe[ad] OR Flevoland[ad] OR Friesland[ad] OR Fryslan[ad] OR Frisia[ad] OR Gelderland[ad] OR Guelders[ad] OR Groningen[ad] OR Limburg[ad] OR Brabant[ad] OR Holland[ad] OR Overijssel[ad] OR Overissel[ad] OR Utrecht[ad] OR Zeeland[ad] OR Amsterdam[ad] OR Rotterdam[ad] OR Hague[ad] OR "s-Gravenhage"[ad] OR "Den Haag"[ad] OR Eindhoven[ad] OR Tilburg[ad] OR Almere[ad] OR Breda[ad] OR Nijmegen[ad] OR Nimeguen[ad]) OR (Malta[ad] OR Maltese*[ad] OR Maltin[ad] OR Gozo[ad] OR Ghawdex[ad] OR Valletta[ad] OR "Ill Belt"[ad] OR Birkirkara[ad] OR "B Kara"[ad] OR "B'Kara"[ad] OR Birchircara[ad] OR Mosta[ad] OR Qormi[ad] OR "St Paul s Bay"[ad] OR "St Paul's Bay"[ad] OR "Pawl il Bahar"[ad] OR Zabbar[ad] OR Sliema[ad] OR Naxxar[ad] OR Gwann[ad] OR "St John"[ad] OR Zebbug[ad] OR "Citta rohan"[ad] OR Fgura[ad]) OR (Luxembourg*[ad] OR Luxemburg[ad] OR Letzebuerg[ad] OR Diekirch[ad] OR Grevenmacher[ad] OR "Esch sur Alzette"[ad] OR "Esch Uelzecht"[ad] OR "Esch an der Alzette"[ad] OR "Esch an der Alzig"[ad] OR Dudelange[ad] OR Diddeleng[ad] OR Dudelingen[ad] OR Duedelingen[ad] OR Schifflange[ad] OR Scheffleng[ad] OR Schifflingen[ad] OR Bettembourg[ad] OR Beetebuerg[ad] OR Bettemburg[ad] OR Petange[ad] OR Peiteng[ad] OR Petingen[ad] OR Ettelbruck[ad] OR Ettelbreck[ad] OR Ettelbrueck[ad] OR Diekirch[ad] OR Dikrech[ad] OR Strassen[ad] OR Stroossen[ad] OR Bertrange[ad] OR Bartreng[ad] OR Bartringen[ad]) OR (Lithuania*[ad] OR "Lietuvos Respublika"[ad] OR Lietuva[ad] OR lietuviu[ad] OR Alytus[ad] OR Alytaus[ad] OR Kaunas[ad] OR Kauno[ad] OR Klaipeda[ad] OR Klaipedos[ad] OR	

	Marijampoles[ad] OR Marijampole[ad] OR Panevezys[ad] OR Panevezio[ad] OR Siauliai[ad] OR Siauliu[ad] OR Taurages[ad] OR Taurage[ad] OR Telsiu[ad] OR Telsiai[ad] OR Utenos[ad] OR Utena[ad] OR Vilnius[ad] OR Vilniaus[ad] OR Mazeikiai[ad] OR Jonava[ad] OR Mazeikiu[ad] OR Jonavos[ad]) OR (Latvi*[ad] OR Riga[ad] OR Courland[ad] OR Kurzeme[ad] OR Kurland[ad] OR Latgale[ad] OR Lettgallia[ad] OR Latgola[ad] OR Latgalia[ad] OR Vidzeme[ad] OR Vidumo[ad] OR Semigallia[ad] OR Semigalia[ad] OR Zemgale[ad] OR Pieriga[ad] OR Daugavpils[ad] OR Dinaburg[ad] OR Jekabpils[ad] OR Jakobstadt[ad] OR Jelgava[ad] OR Jurmala[ad] OR Liepaja[ad] OR Libau[ad] OR Rezekne[ad] OR Rezne[ad] OR Rositten[ad] OR Valmiera[ad] OR Wolmar[ad] OR Ventspils[ad] OR Windau[ad] OR Ogre[ad]) OR (Italy[ad] OR Italia*[ad] OR Abruzzo[ad] OR Abruzzi[ad] OR Basilicata[ad] OR Lucania[ad] OR Calabria[ad] OR Campania[ad] OR "Emilia Romagna"[ad] OR "friuli venezia giulia"[ad] OR Lazio[ad] OR Latium[ad] OR Liguria*[ad] OR Lombardy[ad] OR Lombardia[ad] OR Marche[ad] OR Marches[ad] OR Molisano[ad] OR Molise[ad] OR Piedmont*[ad] OR Piemonte[ad] OR Bolzano[ad] OR Bozen[ad] OR Trentino[ad] OR Trento[ad] OR Puglia[ad] OR Apulia[ad] OR Sardinia[ad] OR Sardegna[ad] OR Sicily[ad] OR Sicilia[ad] OR Toscana[ad] OR Tuscany[ad] OR Umbria[ad] OR "Valle d Aosta"[ad] OR "Vallee d Aoste"[ad] OR "Valle d'Aosta"[ad] OR "Vallee d'Aoste"[ad] OR "Aosta Valley"[ad] OR Veneto[ad] OR Venetia[ad] OR Triveneto[ad] OR Rome[ad] OR Roma[ad] OR Milan[ad] OR Milano[ad] OR Naples[ad] OR Napoli[ad] OR Turin[ad] OR Torino[ad] OR Palermo[ad] OR Genoa[ad] OR Genova[ad] OR Bologna[ad] OR Florence[ad] OR Firenze[ad] OR Bari[ad] OR Catania[ad]) OR (Ireland[ad] OR Eire[ad] OR Irish*[ad] OR Fingal[ad] OR "Fine Gall"[ad] OR Dublin[ad] OR "Ath Cliath"[ad] OR "Dun Laoghaire"[ad] OR Wicklow[ad] OR "Cill Mhantain"[ad] OR "Chill Mhantain"[ad] OR Wexford[ad] OR "Loch Garman"[ad] OR Carlow[ad] OR Ceatharlach[ad] OR Kildare[ad] OR "Cill Dara"[ad] OR "Chill Dara"[ad] OR Meath[ad] OR "An Mhi"[ad] OR "Contae na Mi"[ad] OR Louth[ad] OR "Contae Lu"[ad] OR Monaghan[ad] OR Muineachan[ad] OR Mhuineachain[ad] OR Cavan[ad] OR "An Cabhan"[ad] OR "An Cabhain"[ad] OR Longford[ad] OR "An Longfort"[ad] OR "an Longfoirt"[ad] OR Langfurd[ad] OR Westmeath[ad] OR "An Iarmhi"[ad] OR "na Iarmhi"[ad] OR Offaly[ad] OR "Uibh Fhaili"[ad] OR Laois[ad] OR Laoise[ad] OR Kilkenny[ad] OR "Chill Chainnigh"[ad] OR "Cill Chainnigh"[ad] OR Waterford[ad] OR "Port Lairge"[ad] OR Watterford[ad] OR Cork[ad] OR Corcaigh[ad] OR Chorcai[ad] OR Kerry[ad] OR Ciarrai[ad] OR Chiarrai[ad] OR Limerick[ad] OR Luimneach[ad] OR Luimnigh[ad] OR Tipperary[ad] OR "Tiobraid Arann"[ad] OR "Thiobraid Arann"[ad] OR Clare[ad] OR "An Clar"[ad] OR "an Chlair"[ad] OR Galway[ad] OR Gaillimh[ad] OR "na Gaillimhe"[ad] OR Mayo[ad] OR "Maigh Eo"[ad] OR "Mhaigh Eo"[ad] OR Roscommon[ad] OR "Ros comain"[ad] OR Sligo[ad] OR Sligeach[ad] OR Shligigh[ad] OR Leitrim[ad] OR Liatroim[ad] OR Liatroma[ad] OR Donegal[ad] OR "Dhun na nGall"[ad] OR Dinnygal[ad] OR Dunnyga[ad] OR Leinster[ad] OR Laighin[ad] OR "Cuige Laighean"[ad] OR Munster[ad] OR Mumhain[ad] OR "Cuige Mumhan"[ad] OR Connacht[ad] OR Connachta[ad] OR Drogheda[ad] OR "Droichead Atha"[ad] OR Dundalk[ad] OR "Dun Dealgan"[ad] OR Swords[ad] OR Sord[ad] OR Bray[ad] OR Bre[ad] OR Navan[ad] OR "An Uaimh"[ad]) OR (Hungar*[ad] OR Magyarorszag[ad] OR Magyar*[ad] OR Dunantuli[ad] OR Transdanubia[ad] OR Dunantul[ad] OR "Great Plain"[ad] OR "Eszak Alfold"[ad] OR "Del Alfold"[ad] OR "Alfold es eszak"[ad] OR "Northern Alfold"[ad] OR "North Alfold"[ad] OR "South Alfold"[ad] OR "Southern Alfold"[ad] OR Bacs[ad] OR Kiskun[ad] OR Baranya[ad] OR Bekes[ad] OR Borsod[ad] OR Abauj[ad] OR Zemplen[ad] OR Budapest[ad] OR Csongrad[ad] OR Fejer[ad] OR gyor[ad] OR moson[ad] OR sopron[ad] OR hajdu[ad] OR bihar[ad] OR Heves[ad] OR "jasz nagykun szolnok"[ad] OR komarom[ad] OR esztergom[ad] OR Nograd[ad] OR (Pest[ad] AND (megye[ad] OR county[ad])) OR Somogy[ad] OR szabolcs[ad] OR szatmar[ad] OR bereg[ad] OR Tolna[ad] OR Vas[ad] OR Veszprem[ad] OR Zala[ad] OR Debrecen[ad] OR Miskolc[ad] OR Szeged[ad] OR Pecs[ad] OR Gyor[ad] OR Nyiregyhaza[ad] OR Kecskemet[ad] OR Szekesfehervar[ad] OR Szombathely[ad]) OR (Greece[ad] OR "Hellenic republic"[ad] OR Greek*[ad] OR Ellada[ad] OR Elladas[ad] OR "Elliniki	

	Dimokratia"[ad] OR Hellas[ad] OR Hellenes[ad] OR Attica[ad] OR Attiki[ad] OR Makedonia*[ad] OR Macedonia[ad] OR Thraki[ad] OR Thrace[ad] OR Crete[ad] OR Kriti[ad] OR "Ionia Nisia"[ad] OR "Ionion neson"[ad] OR "Ionion nIson"[ad] OR "Ionian islands"[ad] OR "Ionian island"[ad] OR Epirus[ad] OR Ipeiros[ad] OR "Perifereia Ipeirou"[ad] OR "North aegean"[ad] OR "Northern Aegean"[ad] OR "Aegean islands"[ad] OR "Aegean island"[ad] OR "Nisoi Agaiou"[ad] OR "Notio Aigaio"[ad] OR Peloponnese[ad] OR Peloponniso*[ad] OR Thessaly[ad] OR Thessalia[ad] OR Thessalian[ad] OR Petthalia[ad] OR "Voreio Aigaio"[ad] OR "Voreio Aigaiou"[ad] OR "South aegean"[ad] OR "Southern Aegean"[ad] OR "Mount athos"[ad] OR "Oros Athos"[ad] OR Cyclades[ad] OR Cycklades[ad] OR Kiklades[ad] OR Dodecanese[ad] OR Dodekanisa[ad] OR Athens[ad] OR Athina[ad] OR Thessaloniki[ad] OR Thessalonica[ad] OR Patras[ad] OR Patra[ad] OR Pireas[ad] OR Piraeus[ad] OR Larissa[ad] OR Larisa[ad] OR Heraklion[ad] OR Heraclion[ad] OR Iraklion[ad] OR Irakleion[ad] OR Iraklio[ad] OR Volos[ad] OR Rhodes[ad] OR Rodos[ad] OR Ioannina[ad] OR Janina[ad] OR Yannena[ad] OR Chania[ad] OR Chalcis[ad] OR Chalkida[ad]) OR (German*[ad] OR Deutsch*[ad] OR Bundesrepublik[ad] OR Westdeutschland[ad] OR Ostdeutschland[ad] OR Baden[ad] OR Wuerttemberg[ad] OR Wurttemberg[ad] OR Bayern[ad] OR Bavaria[ad] OR Berlin[ad] OR Brandenburg[ad] OR Bremen[ad] OR Oldenburg[ad] OR Mitteldeutschland[ad] OR Rhein[ad] OR Rhine[ad] OR Hannover[ad] OR Braunschweig[ad] OR Gottingen[ad] OR Goettingen[ad] OR Nurnberg[ad] OR Nuernberg[ad] OR Ruhr[ad] OR Koln[ad] OR koeln[ad] OR Bonn[ad] OR Hamburg[ad] OR Hessen[ad] OR Hesse[ad] OR Hessia[ad] OR Mecklenburg[ad] OR Vorpommern[ad] OR Pomerania[ad] OR Niedersachsen[ad] OR Neddersassen[ad] OR Saxony[ad] OR Niederbayern[ad] OR "Northern Rhine"[ad] OR "North Rhine"[ad] OR Westphalia[ad] OR Westfalen[ad] OR "Rhineland Palatinate"[ad] OR "Rheinland Pfalz"[ad] OR Saarland[ad] OR Sachsen[ad] OR "Schleswig Holstein"[ad] OR Thuringia[ad] OR Thuringen[ad] OR Thueringen[ad] OR Munchen[ad] OR Muenchen[ad] OR Munich[ad] OR Frankfurt[ad] OR Stuttgart[ad] OR Dusseldorf[ad] OR Duesseldorf[ad] OR Dortmund[ad] OR Essen[ad]) OR (France[ad] OR French*[ad] OR Francais*[ad] OR Alsace[ad] OR Elsass[ad] OR Aquitaine[ad] OR Aquitania[ad] OR Akitania[ad] OR Aguiene[ad] OR Auvergne[ad] OR Auvernhe[ad] OR Auvernha[ad] OR Normandie[ad] OR Normandy[ad] OR Normaundie[ad] OR Bourgogne[ad] OR Burgundy[ad] OR Bregogne[ad] OR Borgoegne[ad] OR Borgogne[ad] OR Brittany[ad] OR Breizh[ad] OR Bertaeyn[ad] OR Bretagne[ad] OR "Champagne Ardenne"[ad] OR Corse[ad] OR Corsica[ad] OR "Franche Comte"[ad] OR "Frantche Comte"[ad] OR "Franche Comtat"[ad] OR Guadeloupe[ad] OR Guyane[ad] OR Guiana[ad] OR "Languedoc Roussillon"[ad] OR "Lengadoc Rosselhon"[ad] OR "Llenguadoc-Rossello"[ad] OR Limousin[ad] OR Lemosin[ad] OR Lorraine[ad] OR Lothringen[ad] OR Lottringe[ad] OR Martinique[ad] OR "Midi Pyrenees"[ad] OR "Miegjorn Pireneus"[ad] OR "Mieidia Pireneus"[ad] OR "Mediodia Pirineos"[ad] OR "Pays de la Loire"[ad] OR "Broiou al Liger"[ad] OR Picardie[ad] OR Picardy[ad] OR "Poitou Charentes"[ad] OR "Peitau Charantas"[ad] OR "Poetou-Cherentes"[ad] OR Provence[ad] OR Provenca[ad] OR Prouvenco[ad] OR "Cote d Azur"[ad] OR "Cote d'Azur"[ad] OR "Costo d'Azur"[ad] OR "Costo d Azur"[ad] OR "Costa d'Azur"[ad] OR "Costa d Azur"[ad] OR Reunion[ad] OR "Rhone Alpes"[ad] OR "Rono Arpes"[ad] OR "Rose Aups"[ad] OR Ain[ad] OR Aisne[ad] OR Allier[ad] OR "Alpes de Haute Provence"[ad] OR "Haute Alpes"[ad] OR "Alpes Maritimes"[ad] OR Ardeche[ad] OR Ardennes[ad] OR Ariege[ad] OR Aube[ad] OR Aude[ad] OR Aveyron[ad] OR "Bas Rhin"[ad] OR "Bouches du Rhone"[ad] OR Calvados[ad] OR Cantal[ad] OR Charente[ad] OR Cher[ad] OR Correze[ad] OR "Corse du Sud"[ad] OR "Cote d Or"[ad] OR "Cote d'Or"[ad] OR "Cotes d Armor"[ad] OR "Cotes d'Armor"[ad] OR Creuse[ad] OR "Deux Sevres"[ad] OR Dordogne[ad] OR Doubs[ad] OR Drome[ad] OR Essonne[ad] OR Eure[ad] OR Finistere[ad] OR Gard[ad] OR Gers[ad] OR Gironde[ad] OR "Haute Corse"[ad] OR "Haute Garonne"[ad] OR "Haute Marne"[ad] OR "Hautes Alpes"[ad] OR "Haute Saone"[ad] OR "Haute Savoie"[ad] OR "Hautes Pyrenees"[ad] OR "Haute Vienne"[ad] OR "Haut Rhin"[ad] OR "Hauts de Seine"[ad] OR Herault[ad] OR "Ile de France"[ad] OR	

	"Ille et Vilaine"[ad] OR Indre[ad] OR Isere[ad] OR Jura[ad] OR Landes[ad] OR Loire[ad] OR Loiret[ad] OR (Lot[ad] AND (departement[ad] OR department[ad])) OR "Lot et Garonne"[ad] OR "Loir et Cher"[ad] OR Lozere[ad] OR Manche[ad] OR Marne[ad] OR Mayenne[ad] OR Mayotte[ad] OR "Meurthe et Moselle"[ad] OR Meuse[ad] OR Morbihan[ad] OR Moselle[ad] OR (Nord[ad] AND (department[ad] OR departement[ad])) OR Nievre[ad] OR Oise[ad] OR Orne[ad] OR "Pas de calais"[ad] OR "Noord-Nauw van Kales"[ad] OR Paris[ad] OR "Puy de dome"[ad] OR "Pyrenees Atlantiques"[ad] OR "Pyrenees Orientales"[ad] OR Rhone[ad] OR Sarthe[ad] OR Savoie[ad] OR "Seine et Marne"[ad] OR "Seine Maritime"[ad] OR Somme[ad] OR Tarn[ad] OR "Territoire de Belfort"[ad] OR "Val de Marne"[ad] OR "Val d Oise"[ad] OR Var[ad] OR Vaucluse[ad] OR Vendee[ad] OR Vienne[ad] OR Vosges[ad] OR Yonne[ad] OR Yvelines[ad] OR Marseille[ad] OR Lyon[ad] OR Nice[ad] OR Nantes[ad] OR Strasbourg[ad] OR Montpellier[ad] OR Bordeaux[ad] OR Lille[ad] OR Toulouse[ad] OR "Outre Mer"[ad] OR "Seine Saint Denis"[ad]) OR (Finland[ad] OR Finnish*[ad] OR Finn[ad] OR Finns[ad] OR Suomi[ad] AND Suomen[ad] OR Suomalaiset[ad] OR Aland[ad] OR Ahvenanmaa[ad] OR Uusimaa[ad] OR Nyland[ad] OR Karelia[ad] OR Karjala[ad] OR Karelen[ad] OR Ostrobothnia[ad] OR Pohjanmaa[ad] OR Osterbotten[ad] OR Savonia[ad] OR Savo[ad] OR Savolax[ad] OR Kainuu[ad] OR Kajanaland*[ad] OR "Kanta Hame"[ad] OR Tavastia[ad] OR Tavastland[ad] OR Kymenlaakso[ad] OR Kymmenedalen[ad] OR Lapland[ad] OR Lappi[ad] OR Lappland[ad] OR "Paijat Hame"[ad] OR Pirkanmaa[ad] OR Birkaland[ad] OR Satakunta[ad] OR Satakunda[ad] OR Helsinki[ad] OR Helsingfors[ad] OR Espoo[ad] OR Esbo[ad] OR Tampere[ad] OR Tammerfors[ad] OR Vantaa[ad] OR Vanda[ad] OR Oulu[ad] OR Uleaborg[ad] OR Turku[ad] OR Abo[ad] OR Jyvaskyla[ad] OR Kuopio[ad] OR Lahti[ad] OR Lahtis[ad] OR Kouvola[ad]) OR (Estonia*[ad] OR Eesti[ad] OR Eestlased[ad] OR Eestlane[ad] OR Harju[ad] OR Harjumaa[ad] OR Hiiu[ad] OR Hiiumaa[ad] OR "Ida Viru"[ad] OR "Ida Virumaa"[ad] OR Jarvamaa[ad] OR Jarva[ad] OR Jogevamaa[ad] OR Jogeva[ad] OR Laanemaa[ad] OR Laane[ad] OR "Laane Virumaa"[ad] OR Parnu[ad] OR Parnumaa[ad] OR Polva[ad] OR Polvamaa[ad] OR Rapla[ad] OR Raplamaa[ad] OR Saare[ad] OR Saaremaa[ad] OR Tartu[ad] OR Tartumaa[ad] OR Valga[ad] OR Valgamaa[ad] OR Viljandimaa[ad] OR Viljandi[ad] OR Voru[ad] OR Vorumaa[ad] OR Tallinn[ad] OR Narva[ad] OR "Kohtla Jarve"[ad] OR Rakvere[ad] OR Maardu[ad] OR Sillamae[ad] OR Kuressaare[ad]) OR (Denmark[ad] OR Danish*[ad] OR dane[ad] OR danes[ad] OR Danmark[ad] OR dansk*[ad] OR Hovedstaden[ad] OR Midtjylland[ad] OR Nordjylland[ad] OR Sjaelland[ad] OR Sealand[ad] OR "Zealand region"[ad] OR "region Zealand"[ad] OR Syddanmark[ad] OR Jutland[ad] OR Jylland[ad] OR Sonderjyllands[ad] OR Copenhagen[ad] OR Kobenhavn[ad] OR Arhus[ad] OR Aarhus[ad] OR Bornholm[ad] OR Frederiksberg[ad] OR Frederiksborg[ad] OR Ringkjobing[ad] OR Viborg[ad] OR Vejle[ad] OR Roskilde[ad] OR Storstrom[ad] OR Vestsjaellands[ad] OR "West Zealand"[ad] OR Funen[ad] OR Ribe[ad] OR "Kalaallit Nunaat"[ad] OR Gronland[ad] OR Foroyar[ad] OR Faeroerne[ad] OR "Faroe islands"[ad] OR Aalborg[ad] OR Alborg[ad] OR Odense[ad] OR Esbjerg[ad] OR Gentofte[ad] OR Gladsaxe[ad] OR Randers[ad] OR Kolding[ad]) OR (Czech*[ad] OR Cesky[ad] OR Ceska[ad] OR Cech[ad] OR Cestina[ad] OR Prague[ad] OR Praha[ad] OR Prag[ad] OR Stredoces*[ad] OR Jihoces*[ad] OR Bohemia[ad] OR Bohemian[ad] OR Plzen*[ad] OR Pilsen[ad] OR Karlovars*[ad] OR "Karlovy Vary"[ad] OR Usteck*[ad] OR Usti[ad] OR Liberec*[ad] OR "Hradec Kralove"[ad] OR Kralovehradec*[ad] OR Pardubic*[ad] OR Olomouc*[ad] OR Olomoc[ad] OR Holomoc[ad] OR Moravskoslezs*[ad] OR Jihomorav*[ad] OR Moravia[ad] OR Moravian[ad] OR Morava[ad] OR Vysocina[ad] OR Zlin[ad] OR Zlinsk*[ad] OR "Ceske Budejovice"[ad] OR Budweis[ad] OR Brno[ad] OR Ostrava[ad]) OR (Cyprus[ad] OR Cypriot*[ad] OR Kypros[ad] OR Kibris*[ad] OR kypriaki[ad] OR Kyprioi[ad] OR Nicosia[ad] OR Lefkosa[ad] OR Lefkosia[ad] OR Famagusta[ad] OR Magusa[ad] OR Ammochostos[ad] OR Gazimagusa[ad] OR Kyrenia[ad] OR Girne[ad] OR Keryneia[ad] OR Larnaca[ad] OR Larnaka[ad] OR Iskele[ad] OR Limassol[ad] OR Lemesos[ad] OR Limasol[ad] OR Leymosun[ad] OR Paphos[ad] OR Pafos[ad] OR Baf[ad] OR Strovolos[ad] OR Lakatamia[ad] OR Lakadamya[ad] OR "Kato Polemidia"[ad] OR	

	"Kato Polemidhia"[ad] OR Aglandjia[ad] OR Eglence[ad] OR Aglantzia[ad] OR Aradhippou[ad] OR Aradippou[ad] OR Engomi[ad]) OR (Croat*[ad] OR Hrvatsk*[ad] OR hrvati[ad] OR Bjelovar[ad] OR "Bjelovarsko bilogorska"[ad] OR "Brod Posavina"[ad] OR "Brodsko posavska"[ad] OR "Dubrovnik Neretva"[ad] OR "dubrovacko neretvanska"[ad] OR Zagreb[ad] OR Zagrebacka[ad] OR Istria[ad] OR Istarska[ad] OR Karlovacka[ad] OR Karlovac[ad] OR "Koprivnicko krizevacka"[ad] OR Koprivnica[ad] OR Krizevci[ad] OR "Krapina Zagorje"[ad] OR "Krapinsko zagorska"[ad] OR "Lika Senj"[ad] OR "Licko senjska"[ad] OR Medimurska[ad] OR Medimurje[ad] OR Osijek[ad] OR Baranja[ad] OR "Osjecko baranjska"[ad] OR "Pozega Slavonia"[ad] OR "Pozesko slavonska"[ad] OR "Primorje Gorski Kotar"[ad] OR "Primorsko goranska"[ad] OR "Sibensko kninska"[ad] OR "Sibensko kninske"[ad] OR Sibenik[ad] OR Knin[ad] OR Sisak[ad] OR "Sisacko moslavacka"[ad] OR Moslavina[ad] OR "Splitsko dalmatinska"[ad] OR Split[ad] OR Dalmatia[ad] OR Varazdin[ad] OR Varazdinska[ad] OR Viroviticko[ad] OR podravska[ad] OR Virovitica[ad] OR Podravina[ad] OR "Vukovarsko srijemska"[ad] OR Vukovar[ad] OR Srijem[ad] OR Zadar[ad] OR Zadarska[ad] OR Rijeka[ad] OR "Velika gorica"[ad] OR "Slavonski brod"[ad] OR Pula[ad]) OR (Bulgaria*[ad] OR Balgariya[ad] OR Balgarija[ad] OR Blagoevgrad*[ad] OR "Pirin Macedonia"[ad] OR Burgas[ad] OR Dobrich[ad] OR Gabrovo[ad] OR Haskovo[ad] OR Kardzhali[ad] OR Kurdzhali[ad] OR Kyustendil[ad] OR Lovech[ad] OR Lovec[ad] OR Montana[ad] OR Pazardzhik[ad] OR Pernik[ad] OR Pleven*[ad] OR Plovdiv[ad] OR Razgrad[ad] OR Rousse[ad] OR Ruse[ad] OR Rusenka[ad] OR Shumen[ad] OR Silistra[ad] OR Sliven[ad] OR Smolyan[ad] OR Sofia[ad] OR Sofyiska[ad] OR Sofiiska[ad] OR "Stara Zagora"[ad] OR Targovishte[ad] OR Varna[ad] OR "Veliko Tarnovo"[ad] OR Vidin[ad] OR Vratsa[ad] OR Vratza[ad] OR Yambol[ad]) OR (Belgi*[ad] OR Belge*[ad] OR Belg[ad] OR Brussel*[ad] OR Bruxelles[ad] OR Bruxelloise[ad] OR Walloon*[ad] OR Wallon*[ad] OR Vlaams[ad] OR Flander*[ad] OR Flandern[ad] OR Flandre[ad] OR Flemish[ad] OR Flamand[ad] OR Flemisch[ad] OR Flamisch*[ad] OR Vlaanderen[ad] OR Flamande[ad] OR Waals[ad] OR Antwerp*[ad] OR Anvers[ad] OR Henegouwen[ad] OR Hennegau[ad] OR Hainault[ad] OR Hainaut[ad] OR Liege[ad] OR Luik[ad] OR Luttich[ad] OR Limbourg[ad] OR Limburg[ad] OR Namur[ad] OR Namen[ad] OR Ostflandern[ad] OR Westflandern[ad] OR Ghent[ad] OR Gent[ad] OR Gand[ad] OR Charleroi[ad] OR Bruges[ad] OR Brugge*[ad] OR Schaerbeek[ad] OR Schaarbeek[ad] OR Anderlecht[ad] OR Leuven[ad] OR Louvain[ad]) OR (Austria*[ad] OR Osterreich*[ad] OR Oesterreich*[ad] OR Ostosterreich[ad] OR Ostoesterreich[ad] OR Sudosterreich[ad] OR Sudoesterreich[ad] OR Westosterreich[ad] OR Westoesterreich[ad] OR Burgenland[ad] OR Carinthia[ad] OR Karnten[ad] OR Kaernten[ad] OR Niederosterreich[ad] OR Niederoesterreich[ad] OR Oberosterreich[ad] OR Oberoesterreich[ad] OR Salzburg[ad] OR Saizburg[ad] OR Styria[ad] OR Steiermark[ad] OR Tyrol[ad] OR Tirol[ad] OR Vorarlberg[ad] OR Vienna[ad] OR Wien[ad] OR Graz[ad] OR Linz[ad] OR Innsbruck[ad] OR Klagenfurt[ad] OR Villach[ad] OR Wels[ad] OR "St Polten"[ad] OR "St Poelten"[ad] OR "Sankt Polten"[ad] OR "Sankt Poelten"[ad] OR Dornbirn[ad])	
#16	Search (Iceland[ad] OR Icelandic*[ad] OR islenska*[ad] OR Icelander*[ad] OR islendinga*[ad] OR Islendigar[ad] OR Inslenska[ad] OR Reykjavik[ad] OR Reykjavikurborg[ad] OR Hofudborgarsvaedi[ad] OR Sudurnes[ad] OR Vesturland[ad] OR Vestfirdir[ad] OR Westfjords[ad] OR Nordurland[ad] OR Austurland[ad] OR Sudurland[ad] OR Kopavogur[ad] OR Hafnarfjordur[ad] OR Akureyri[ad] OR Gardabaer[ad] OR Mosfellsbaer[ad] OR Keflavik[ad] OR Akranes[ad] OR Selfoss[ad] OR Seltjarnarnes[ad]) OR (Switzerland[ad] OR Schweiz[ad] OR Schweizerische[ad] OR Swiss[ad] OR Suisse*[ad] OR Svizzera[ad] OR Svizzeri[ad] OR Svizzers[ad] OR Svizra[ad] OR Helvetica[ad] OR Aargau[ad] OR Argovia[ad] OR Ausserrhoden[ad] OR "Outer Rhodes"[ad] OR Innerrhoden[ad] OR "Inner Rhodes"[ad] OR Basel[ad] OR Bale[ad] OR Basilea[ad] OR Bern[ad] OR Berne[ad] OR Berna[ad] OR Fribourg[ad] OR Freiburg[ad] OR Friburg[ad] OR Geneva[ad] OR Geneve[ad] OR Genf[ad] OR	460830

	Ginevra[ad] OR Genevra[ad] OR Glarus[ad] OR Graubunden[ad] OR Graubuenden[ad] OR Grisons[ad] OR Grigioni[ad] OR Grischun[ad] OR jura[ad] OR Lucerne[ad] OR Luzern[ad] OR Losanna[ad] OR Neuchatel[ad] OR Nidwalden[ad] OR Nidwald[ad] OR Obwalden[ad] OR Obwald[ad] OR Schaffhausen[ad] OR Schaffhouse[ad] OR Schwyz[ad] OR Solothurn[ad] OR Soleure[ad] OR Thurgau[ad] OR Thurgovia[ad] OR Ticino[ad] OR Tessin[ad] OR Uri[ad] OR Valais[ad] OR Wallis[ad] OR Vaud[ad] OR Zug[ad] OR Zurich[ad] OR Zuerich[ad] OR Zurigo[ad] OR Lausanne[ad] OR Losanna[ad] OR Winterthur[ad] OR Winterthour[ad] OR "St Gallen"[ad] OR "Saint Gallen"[ad] OR "Sankt Gallen"[ad] OR "Saint Gall"[ad] OR "San Gallo"[ad] OR "Son Gagl"[ad] OR Turitg[ad]) OR (Norway[ad] OR Norwegian*[ad] OR Norge[ad] OR Noreg[ad] OR Norgga[ad] OR Akershus[ad] OR "Aust Agder"[ad] OR Buskerud[ad] OR Finnmark[ad] OR Hedmark[ad] OR Hordaland[ad] OR "More og Romsdal"[ad] OR "More and Romsdal"[ad] OR "More Romsdal"[ad] OR Nordland[ad] OR Trondelag[ad] OR Oppland[ad] OR Oslo[ad] OR Ostfold[ad] OR Rogaland[ad] OR "Sogn og fjordane"[ad] OR "Sogn and fjordane"[ad] OR "sogn fjordane"[ad] OR Telemark[ad] OR Troms[ad] OR Romsa[ad] OR Romssa[ad] OR "Vest Agder"[ad] OR Vestfold[ad] OR Bergen[ad] OR Stavanger[ad] OR Sandnes[ad] OR Trondheim[ad] OR Trondhjem[ad] OR Kaupangen[ad] OR Nidaros[ad] OR Drammen[ad] OR Fredrikstad[ad] OR Skien[ad] OR Tromso[ad] OR Sarpsborg[ad]) OR (Liechtenstein[ad] OR Lienchtensteiner*[ad] OR Balzers[ad] OR Eschen[ad] OR Gamprin[ad] OR Mauren[ad] OR Planken[ad] OR Ruggell[ad] OR Schaan[ad] OR Schellenberg[ad] OR Triesen[ad] OR Triesenberg[ad] OR Vaduz[ad])	
#17	Search ("Liechtenstein"[Mesh] OR Liechtenstein[tw] OR Lienchtensteiner*[tw] OR Balzers[tw] OR Eschen[tw] OR Gamprin[tw] OR Mauren[tw] OR Planken[tw] OR Ruggell[tw] OR Schaan[tw] OR Schellenberg[tw] OR Triesen[tw] OR Triesenberg[tw] OR Vaduz[tw])) OR ("Norway"[Mesh] OR Norway[tw] OR Norwegian*[tw] OR Norge[tw] OR Noreg[tw] OR Norgga[tw] OR Akershus[tw] OR "Aust Agder"[tw] OR Buskerud[tw] OR Finnmark[tw] OR Hedmark[tw] OR Hordaland[tw] OR "More og Romsdal"[tw] OR "More and Romsdal"[tw] OR "More Romsdal"[tw] OR Nordland[tw] OR Trondelag[tw] OR Oppland[tw] OR Oslo[tw] OR Ostfold[tw] OR Rogaland[tw] OR "Sogn og fjordane"[tw] OR "Sogn and fjordane"[tw] OR "sogn fjordane"[tw] OR Telemark[tw] OR Troms[tw] OR Romsa[tw] OR Romssa[tw] OR "Vest Agder"[tw] OR Vestfold[tw] OR Bergen[tw] OR Stavanger[tw] OR Sandnes[tw] OR Trondheim[tw] OR Trondhjem[tw] OR Kaupangen[tw] OR Nidaros[tw] OR Drammen[tw] OR Fredrikstad[tw] OR Skien[tw] OR Tromso[tw] OR Sarpsborg[tw])) OR ("Switzerland"[Mesh] OR Switzerland[tw] OR Schweiz[tw] OR Schweizerische[tw] OR Swiss[tw] OR Suisse*[tw] OR Svizzera[tw] OR Svizzeri[tw] OR Svizzers[tw] OR Svizra[tw] OR Helvetica[tw] OR Aargau[tw] OR Argovia[tw] OR Ausserrhoden[tw] OR "Outer Rhodes"[tw] OR Innerrhoden[tw] OR "Inner Rhodes"[tw] OR Basel[tw] OR Bale[tw] OR Basilea[tw] OR Bern[tw] OR Berne[tw] OR Berna[tw] OR Fribourg[tw] OR Freiburg[tw] OR Friburg[tw] OR Geneva[tw] OR Geneve[tw] OR Genf[tw] OR Ginevra[tw] OR Genevra[tw] OR Glarus[tw] OR Graubunden[tw] OR Graubuenden[tw] OR Grisons[tw] OR Grigioni[tw] OR Grischun[tw] OR jura[tw] OR Lucerne[tw] OR Luzern[tw] OR Losanna[tw] OR Neuchatel[tw] OR Nidwalden[tw] OR Nidwald[tw] OR Obwalden[tw] OR Obwald[tw] OR Schaffhausen[tw] OR Schaffhouse[tw] OR Schwyz[tw] OR Solothurn[tw] OR Soleure[tw] OR Thurgau[tw] OR Thurgovia[tw] OR Ticino[tw] OR Tessin[tw] OR Uri[tw] OR Valais[tw] OR Wallis[tw] OR Vaud[tw] OR Zug[tw] OR Zurich[tw] OR Zuerich[tw] OR Zurigo[tw] OR Lausanne[tw] OR Losanna[tw] OR Winterthur[tw] OR Winterthour[tw] OR "St Gallen"[tw] OR "Saint Gallen"[tw] OR "Sankt Gallen"[tw] OR "Saint Gall"[tw] OR "San Gallo"[tw] OR "Son Gagl"[tw] OR Turitg[tw])) OR ("Iceland"[Mesh] OR Iceland[tw] OR Icelandic*[tw] OR islenska*[tw] OR Icelander*[tw] OR islendinga*[tw] OR Islendigar[tw] OR Inslenska[tw] OR Reykjavik[tw] OR Reykjavikurborg[tw] OR Hofudborgarsvaedi[tw] OR Sudurnes[tw] OR Vesturland[tw] OR Vestfirdir[tw] OR Westfjords[tw] OR Nordurland[tw] OR Austurland[tw] OR Sudurland[tw] OR Kopavogur[tw] OR	160173

	Hafnarfjordur[tw] OR Akureyri[tw] OR Gardabaer[tw] OR Mosfellsbaer[tw] OR Keflavik[tw] OR Akranes[tw] OR Selfoss[tw] OR Seltjarnarnes[tw])	
#18	Search ("European Union"[Mesh] OR "Europe"[Mesh:noexp] OR Europe*[tw] OR Europa*[tw] OR EU[tw] OR EEA[tw] OR EFTA[tw] OR "EU/EEA"[tw] OR "EU/EFTA"[tw] OR ECSC[tw] OR Euratom[tw] OR Eurozone[tw] OR EEC[tw] OR ec[tw] OR (Schengen[tw] AND (area[tw] OR countr*[tw] OR region*[tw] OR state[tw] OR states[tw])) OR Euroregion[tw] OR Euroregions[tw] OR "Europe, Eastern"[Mesh:noexp] OR "Balkan Peninsula"[Mesh] OR Balkan[tw] OR Balkans[tw] OR "Baltic States"[Mesh] OR Baltic[tw] OR "Mediterranean Region"[Mesh] OR (Mediterranean[tw] AND (area[tw] OR countr*[tw] OR region*[tw] OR state[tw] OR states[tw])) OR (Alpine[tw] AND (area[tw] OR countr*[tw] OR region*[tw] OR state[tw] OR states[tw])) OR "Scandinavian and Nordic Countries"[Mesh] OR Scandinavia[tw] OR Scandinavian[tw] OR "Nordic country"[tw] OR "Nordic countries"[tw] OR "Nordic state"[tw] OR "Nordic states"[tw] OR Danubian[tw] OR "Iberian peninsula"[tw] OR "Peninsula iberica"[tw] OR "Peninsule Iberique"[tw] OR "Iberiar Penintsula"[tw] OR Iberia[tw] OR Anatolia[tw] OR Anadolu[tw] OR Anatole[tw] OR Anatolian[tw] OR "Yugoslavia"[Mesh] OR Yugoslavia[tw] OR "Czechoslovakia"[Mesh] OR Czechoslovakia[tw] OR "Czecho Slovakia"[tw] OR Ceskoslovensko[tw] OR "Cesko slovensko"[tw] OR Benelux[tw] OR Fennoscandia[tw] OR "Fenno Scandinavia"[tw] OR Fennoskandi*[tw] OR (Visegrad[tw] AND (Group[tw] OR Four[tw] OR Triangle[tw])) OR "Visegradska ctyrka"[tw] OR "Visegradska skupina"[tw] OR "Visegradi Egyuttmukodes"[tw] OR "Visegradi negyek"[tw] OR "Grupa Wyszehradzka"[tw] OR "Vysehradska skupina"[tw] OR "Vysehradska stvorka"[tw]) OR ("Austria"[Mesh] OR Austria*[tw] OR Osterreich*[tw] OR Oesterreich*[tw] OR Ostosterreich[tw] OR Ostoesterreich[tw] OR Sudosterreich[tw] OR Sudoesterreich[tw] OR Westosterreich[tw] OR Westoesterreich[tw] OR Burgenland[tw] OR Carinthia[tw] OR Karnten[tw] OR Kaernten[tw] OR Niederosterreich[tw] OR Niederoesterreich[tw] OR Oberosterreich[tw] OR Oberoesterreich[tw] OR Salzburg[tw] OR Saizburg[tw] OR Styria[tw] OR Steiermark[tw] OR Tyrol[tw] OR Tirol[tw] OR Vorarlberg[tw] OR Vienna[tw] OR Wien[tw] OR Graz[tw] OR Linz[tw] OR Innsbruck[tw] OR Klagenfurt[tw] OR Villach[tw] OR Wels[tw] OR "St Polten"[tw] OR "St Poelten"[tw] OR "Sankt Polten"[tw] OR "Sankt Poelten"[tw] OR Dornbirn[tw]) OR ("Belgium"[Mesh] OR Belgi*[tw] OR Belge*[tw] OR Belg[tw] OR Brussel*[tw] OR Bruxelles[tw] OR Bruxelloise[tw] OR Walloon*[tw] OR Wallon*[tw] OR Vlaams[tw] OR Flander*[tw] OR Flandern[tw] OR Flandre[tw] OR Flemish[tw] OR Flamand[tw] OR Flemisch[tw] OR Flamisch*[tw] OR Vlaanderen[tw] OR Flamande[tw] OR Waals[tw] OR Antwerp*[tw] OR Anvers[tw] OR Henegouwen[tw] OR Hennegau[tw] OR Hainault[tw] OR Hainaut[tw] OR Liege[tw] OR Luik[tw] OR Luttich[tw] OR Limbourg[tw] OR Limburg[tw] OR Namur[tw] OR Namen[tw] OR Ostflandern[tw] OR Westflandern[tw] OR Ghent[tw] OR Gent[tw] OR Gand[tw] OR Charleroi[tw] OR Bruges[tw] OR Brugge*[tw] OR Schaerbeek[tw] OR Schaarbeek[tw] OR Anderlecht[tw] OR Leuven[tw] OR Louvain[tw]) OR ("Bulgaria"[Mesh] OR Bulgaria*[tw] OR Balgariya[tw] OR Balgarija[tw] OR Blagoevgrad*[tw] OR "Pirin Macedonia"[tw] OR Burgas[tw] OR Dobrich[tw] OR Gabrovo[tw] OR Haskovo[tw] OR Kardzhali[tw] OR Kurdzhali[tw] OR Kyustendil[tw] OR Lovech[tw] OR Lovec[tw] OR Montana[tw] OR Pazardzhik[tw] OR Pernik[tw] OR Pleven*[tw] OR Plovdiv[tw] OR Razgrad[tw] OR Rousse[tw] OR Ruse[tw] OR Rusenka[tw] OR Shumen[tw] OR Silistra[tw] OR Sliven[tw] OR Smolyan[tw] OR Sofia[tw] OR Sofyiska[tw] OR Sofiiska[tw] OR "Stara Zagora"[tw] OR Targovishte[tw] OR Varna[tw] OR "Veliko Tarnovo"[tw] OR Vidin[tw] OR Vratsa[tw] OR Vratza[tw] OR Yambol[tw]) OR ("Croatia"[Mesh] OR Croat*[tw] OR Hrvatsk*[tw] OR hrvati[tw] OR Bjelovar[tw] OR "Bjelovarsko bilogorska"[tw] OR "Brod Posavina"[tw] OR "Brodsko posavska"[tw] OR "Dubrovnik Neretva"[tw] OR "dubrovacko neretvanska"[tw] OR Zagreb[tw] OR Zagrebacka[tw] OR Istria[tw] OR Istarska[tw] OR Karlovacka[tw] OR Karlovac[tw] OR "Koprivnicko krizevacka"[tw] OR Koprivnica[tw] OR	3891570

	Krizevci[tw] OR "Krapina Zagorje"[tw] OR "Krapinsko zagorska"[tw] OR "Lika Senj"[tw] OR "Licko senjska"[tw] OR Medimurska[tw] OR Medimurje[tw] OR Osijek[tw] OR Baranja[tw] OR "Osjecko baranjska"[tw] OR "Pozega Slavonia"[tw] OR "Pozesko slavonska"[tw] OR "Primorje Gorski Kotar"[tw] OR "Primorsko goranska"[tw] OR "Sibensko kninska"[tw] OR "Sibensko kninske"[tw] OR Sibenik[tw] OR Knin[tw] OR Sisak[tw] OR "Sisacko moslavacka"[tw] OR Moslavina[tw] OR "Splitsko dalmatinska"[tw] OR Split[tw] OR Dalmatia[tw] OR Varazdin[tw] OR Varazdinska[tw] OR Viroviticko[tw] OR podravska[tw] OR Virovitica[tw] OR Podravina[tw] OR "Vukovarsko srijemska"[tw] OR Vukovar[tw] OR Srijem[tw] OR Zadar[tw] OR Zadarska[tw] OR Rijeka[tw] OR "Velika gorica"[tw] OR "Slavonski brod"[tw] OR Pula[tw]) OR ("Cyprus"[Mesh] OR Cyprus[tw] OR Cypriot*[tw] OR Kypros[tw] OR Kibris*[tw] OR kypriaki[tw] OR Kyprioi[tw] OR Nicosia[tw] OR Lefkosa[tw] OR Lefkosia[tw] OR Famagusta[tw] OR Magusa[tw] OR Ammochostos[tw] OR Gazimagusa[tw] OR Kyrenia[tw] OR Girne[tw] OR Keryneia[tw] OR Larnaca[tw] OR Larnaka[tw] OR Iskele[tw] OR Limassol[tw] OR Lemesos[tw] OR Limasol[tw] OR Leymosun[tw] OR Paphos[tw] OR Pafos[tw] OR Baf[tw] OR Strovolos[tw] OR Lakatamia[tw] OR Lakadamya[tw] OR "Kato Polemidia"[tw] OR "Kato Polemidhia"[tw] OR Aglandjia[tw] OR Eglence[tw] OR Aglantzia[tw] OR Aradhippou[tw] OR Aradippou[tw] OR Engomi[tw]) OR ("Czech Republic"[Mesh] OR Czech*[tw] OR Cesky[tw] OR Ceska[tw] OR Cech[tw] OR Cestina[tw] OR Prague[tw] OR Praha[tw] OR Prag[tw] OR Stredoces*[tw] OR Jihoces*[tw] OR Bohemia[tw] OR Bohemian[tw] OR Plzen*[tw] OR Pilsen[tw] OR Karlovars*[tw] OR "Karlovy Vary"[tw] OR Usteck*[tw] OR Usti[tw] OR Liberec*[tw] OR "Hradec Kralove"[tw] OR Kralovehradec*[tw] OR Pardubic*[tw] OR Olomouc*[tw] OR Olomoc[tw] OR Holomoc[tw] OR Moravskoslezs*[tw] OR Jihomorav*[tw] OR Moravia[tw] OR Moravian[tw] OR Morava[tw] OR Vysocina[tw] OR Zlin[tw] OR Zlinsk*[tw] OR "Ceske Budejovice"[tw] OR Budweis[tw] OR Brno[tw] OR Ostrava[tw]) OR ("Denmark"[Mesh] OR Denmark[tw] OR Danish*[tw] OR dane[tw] OR danes[tw] OR Danmark[tw] OR dansk*[tw] OR Hovedstaden[tw] OR Midtjylland[tw] OR Nordjylland[tw] OR Sjaelland[tw] OR Sealand[tw] OR "Zealand region"[tw] OR "region Zealand"[tw] OR Syddanmark[tw] OR Jutland[tw] OR Jylland[tw] OR Sonderjyllands[tw] OR Copenhagen[tw] OR Kobenhavn[tw] OR Arhus[tw] OR Aarhus[tw] OR Bornholm[tw] OR Frederiksberg[tw] OR Frederiksborg[tw] OR Ringkjobing[tw] OR Viborg[tw] OR Vejle[tw] OR Roskilde[tw] OR Storstrom[tw] OR Vestsjaellands[tw] OR "West Zealand"[tw] OR Funen[tw] OR Ribe[tw] OR "Kalaallit Nunaat"[tw] OR Gronland[tw] OR Foroyar[tw] OR Faeroerne[tw] OR "Faroe islands"[tw] OR Aalborg[tw] OR Alborg[tw] OR Odense[tw] OR Esbjerg[tw] OR Gentofte[tw] OR Gladsaxe[tw] OR Randers[tw] OR Kolding[tw]) OR ("Estonia"[Mesh] OR Estonia*[tw] OR Eesti[tw] OR Eestlased[tw] OR Eestlane[tw] OR Harju[tw] OR Harjumaa[tw] OR Hiiu[tw] OR Hiiumaa[tw] OR "Ida Viru"[tw] OR "Ida Virumaa"[tw] OR Jarvamaa[tw] OR Jarva[tw] OR Jogevamaa[tw] OR Jogeva[tw] OR Laanemaa[tw] OR Laane[tw] OR "Laane Virumaa"[tw] OR Parnu[tw] OR Parnumaa[tw] OR Polva[tw] OR Polvamaa[tw] OR Rapla[tw] OR Raplamaa[tw] OR Saare[tw] OR Saaremaa[tw] OR Tartu[tw] OR Tartumaa[tw] OR Valga[tw] OR Valgamaa[tw] OR Viljandimaa[tw] OR Viljandi[tw] OR Voru[tw] OR Vorumaa[tw] OR Tallinn[tw] OR Narva[tw] OR "Kohtla Jarve"[tw] OR Rakvere[tw] OR Maardu[tw] OR Sillamae[tw] OR Kuressaare[tw]) OR ("Finland"[Mesh] OR Finland[tw] OR Finnish*[tw] OR Finn[tw] OR Finns[tw] OR Suomi[tw] AND Suomen[tw] OR Suomalaiset[tw] OR Aland[tw] OR Ahvenanmaa[tw] OR Uusimaa[tw] OR Nyland[tw] OR Karelia[tw] OR Karjala[tw] OR Karelen[tw] OR Ostrobothnia[tw] OR Pohjanmaa[tw] OR Osterbotten[tw] OR Savonia[tw] OR Savo[tw] OR Savolax[tw] OR Kainuu[tw] OR Kajanaland*[tw] OR "Kanta Hame"[tw] OR Tavastia[tw] OR Tavastland[tw] OR Kymenlaakso[tw] OR Kymmenedalen[tw] OR Lapland[tw] OR Lappi[tw] OR Lappland[tw] OR "Paijat Hame"[tw] OR Pirkanmaa[tw] OR Birkaland[tw] OR Satakunta[tw] OR Satakunda[tw] OR Helsinki[tw] OR Helsingfors[tw] OR Espoo[tw] OR Esbo[tw] OR Tampere[tw] OR Tammerfors[tw] OR Vantaa[tw] OR Vanda[tw] OR Oulu[tw] OR Uleaborg[tw] OR Turku[tw] OR Abo[tw] OR Jyvaskyla[tw] OR Kuopio[tw] OR Lahti[tw] OR Lahtis[tw] OR Kouvola[tw]) OR ("France"[Mesh] OR France[tw] OR	

	French*[tw] OR Francais*[tw] OR Alsace[tw] OR Elsass[tw] OR Aquitaine[tw] OR Aquitania[tw] OR Akitania[tw] OR Aguiene[tw] OR Auvergne[tw] OR Auvernhe[tw] OR Auvernha[tw] OR Normandie[tw] OR Normandy[tw] OR Normaundie[tw] OR Bourgogne[tw] OR Burgundy[tw] OR Bregogne[tw] OR Borgoegne[tw] OR Borgogne[tw] OR Brittany[tw] OR Breizh[tw] OR Bertaeyn[tw] OR Bretagne[tw] OR "Champagne Ardenne"[tw] OR Corse[tw] OR Corsica[tw] OR "Franche Comte"[tw] OR "Frantche Comte"[tw] OR "Franche Comtat"[tw] OR Guadeloupe[tw] OR Guyane[tw] OR Guiana[tw] OR "Languedoc Roussillon"[tw] OR "Lengadoc Rosselhon"[tw] OR "Llenguadoc-Rossello"[tw] OR Limousin[tw] OR Lemosin[tw] OR Lorraine[tw] OR Lothringen[tw] OR Lottringe[tw] OR Martinique[tw] OR "Midi Pyrenees"[tw] OR "Miegjorn Pireneus"[tw] OR "Mieidia Pireneus"[tw] OR "Mediodia Pirineos"[tw] OR "Pays de la Loire"[tw] OR "Broiou al Liger"[tw] OR Picardie[tw] OR Picardy[tw] OR "Poitou Charentes"[tw] OR "Peitau Charantas"[tw] OR "Poetou-Cherentes"[tw] OR Provence[tw] OR Provenca[tw] OR Prouvenco[tw] OR "Cote d Azur"[tw] OR "Cote d'Azur"[tw] OR "Costo d'Azur"[tw] OR "Costo d Azur"[tw] OR "Costa d'Azur"[tw] OR "Costa d Azur"[tw] OR Reunion[tw] OR "Rhone Alpes"[tw] OR "Rono Arpes"[tw] OR "Rose Aups"[tw] OR Ain[tw] OR Aisne[tw] OR Allier[tw] OR "Alpes de Haute Provence"[tw] OR "Haute Alpes"[tw] OR "Alpes Maritimes"[tw] OR Ardeche[tw] OR Ardennes[tw] OR Ariege[tw] OR Aube[tw] OR Aude[tw] OR Aveyron[tw] OR "Bas Rhin"[tw] OR "Bouches du Rhone"[tw] OR Calvados[tw] OR Cantal[tw] OR Charente[tw] OR Cher[tw] OR Correze[tw] OR "Corse du Sud"[tw] OR "Cote d Or"[tw] OR "Cote d'Or"[tw] OR "Cotes d Armor"[tw] OR "Cotes d'Armor"[tw] OR Creuse[tw] OR "Deux Sevres"[tw] OR Dordogne[tw] OR Doubs[tw] OR Drome[tw] OR Essonne[tw] OR Eure[tw] OR Finistere[tw] OR Gard[tw] OR Gers[tw] OR Gironde[tw] OR "Haute Corse"[tw] OR "Haute Garonne"[tw] OR "Haute Marne"[tw] OR "Hautes Alpes"[tw] OR "Haute Saone"[tw] OR "Haute Savoie"[tw] OR "Hautes Pyrenees"[tw] OR "Haute Vienne"[tw] OR "Haut Rhin"[tw] OR "Hauts de Seine"[tw] OR Herault[tw] OR "Ile de France"[tw] OR "Ille et Vilaine"[tw] OR Indre[tw] OR Isere[tw] OR Jura[tw] OR Landes[tw] OR Loire[tw] OR Loiret[tw] OR (Lot[tw] AND (departement[tw] OR department[tw])) OR "Lot et Garonne"[tw] OR "Loir et Cher"[tw] OR Lozere[tw] OR Manche[tw] OR Marne[tw] OR Mayenne[tw] OR Mayotte[tw] OR "Meurthe et Moselle"[tw] OR Meuse[tw] OR Morbihan[tw] OR Moselle[tw] OR (Nord[tw] AND (department[tw] OR departement[tw])) OR Nievre[tw] OR Oise[tw] OR Orne[tw] OR "Pas de calais"[tw] OR "Noord-Nauw van Kales"[tw] OR Paris[tw] OR "Puy de dome"[tw] OR "Pyrenees Atlantiques"[tw] OR "Pyrenees Orientales"[tw] OR Rhone[tw] OR Sarthe[tw] OR Savoie[tw] OR "Seine et Marne"[tw] OR "Seine Maritime"[tw] OR Somme[tw] OR Tarn[tw] OR "Territoire de Belfort"[tw] OR "Val de Marne"[tw] OR "Val d Oise"[tw] OR Var[tw] OR Vaucluse[tw] OR Vendee[tw] OR Vienne[tw] OR Vosges[tw] OR Yonne[tw] OR Yvelines[tw] OR Marseille[tw] OR Lyon[tw] OR Nice[tw] OR Nantes[tw] OR Strasbourg[tw] OR Montpellier[tw] OR Bordeaux[tw] OR Lille[tw] OR Toulouse[tw] OR "Outre Mer"[tw] OR "Seine Saint Denis"[tw]) OR ("Germany"[Mesh] OR German*[tw] OR Deutsch*[tw] OR Bundesrepublik[tw] OR Westdeutschland[tw] OR Ostdeutschland[tw] OR Baden[tw] OR Wuerttemberg[tw] OR Wurttemberg[tw] OR Bayern[tw] OR Bavaria[tw] OR Berlin[tw] OR Brandenburg[tw] OR Bremen[tw] OR Oldenburg[tw] OR Mitteldeutschland[tw] OR Rhein[tw] OR Rhine[tw] OR Hannover[tw] OR Braunschweig[tw] OR Gottingen[tw] OR Goettingen[tw] OR Nurnberg[tw] OR Nuernberg[tw] OR Ruhr[tw] OR Koln[tw] OR koeln[tw] OR Bonn[tw] OR Hamburg[tw] OR Hessen[tw] OR Hesse[tw] OR Hessia[tw] OR Mecklenburg[tw] OR Vorpommern[tw] OR Pomerania[tw] OR Niedersachsen[tw] OR Neddersassen[tw] OR Saxony[tw] OR Niederbayern[tw] OR "Northern Rhine"[tw] OR "North Rhine"[tw] OR Westphalia[tw] OR Westfalen[tw] OR "Rhineland Palatinate"[tw] OR "Rheinland Pfalz"[tw] OR Saarland[tw] OR Sachsen[tw] OR "Schleswig Holstein"[tw] OR Thuringia[tw] OR Thuringen[tw] OR Thueringen[tw] OR Munchen[tw] OR Muenchen[tw] OR Munich[tw] OR Frankfurt[tw] OR Stuttgart[tw] OR Dusseldorf[tw] OR Duesseldorf[tw] OR Dortmund[tw] OR Essen[tw]) OR ("Greece"[Mesh] OR Greece[tw] OR "Hellenic republic"[tw] OR Greek*[tw] OR Ellada[tw] OR Elladas[tw] OR	

	"Elliniki Dimokratia"[tw] OR Hellas[tw] OR Hellenes[tw] OR Attica[tw] OR Attiki[tw] OR Makedonia*[tw] OR Macedonia[tw] OR Thraki[tw] OR Thrace[tw] OR Crete[tw] OR Kriti[tw] OR "Ionia Nisia"[tw] OR "Ionion neson"[tw] OR "Ionion nIson"[tw] OR "Ionian islands"[tw] OR "Ionian island"[tw] OR Epirus[tw] OR Ipeiros[tw] OR "Perifereia Ipeirou"[tw] OR "North aegean"[tw] OR "Northern Aegean"[tw] OR "Aegean islands"[tw] OR "Aegean island"[tw] OR "Nisoi Agaiou"[tw] OR "Notio Aigaio"[tw] OR Peloponnese[tw] OR Peloponniso*[tw] OR Thessaly[tw] OR Thessalia[tw] OR Thessalian[tw] OR Petthalia[tw] OR "Voreio Aigaio"[tw] OR "Voreio Aigaiou"[tw] OR "South aegean"[tw] OR "Southern Aegean"[tw] OR "Mount athos"[tw] OR "Oros Athos"[tw] OR Cyclades[tw] OR Cycklades[tw] OR Kiklades[tw] OR Dodecanese[tw] OR Dodekanisa[tw] OR Athens[tw] OR Athina[tw] OR Thessaloniki[tw] OR Thessalonica[tw] OR Patras[tw] OR Patra[tw] OR Pireas[tw] OR Piraeus[tw] OR Larissa[tw] OR Larisa[tw] OR Heraklion[tw] OR Heraclion[tw] OR Iraklion[tw] OR Irakleion[tw] OR Iraklio[tw] OR Volos[tw] OR Rhodes[tw] OR Rodos[tw] OR Ioannina[tw] OR Janina[tw] OR Yannena[tw] OR Chania[tw] OR Chalcis[tw] OR Chalkida[tw]) OR ("Hungary"[Mesh] OR Hungar*[tw] OR Magyarorszag[tw] OR Magyar*[tw] OR Dunantuli[tw] OR Transdanubia[tw] OR Dunantul[tw] OR "Great Plain"[tw] OR "Eszak Alfold"[tw] OR "Del Alfold"[tw] OR "Alfold es eszak"[tw] OR "Northern Alfold"[tw] OR "North Alfold"[tw] OR "South Alfold"[tw] OR "Southern Alfold"[tw] OR Bacs[tw] OR Kiskun[tw] OR Baranya[tw] OR Bekes[tw] OR Borsod[tw] OR Abauj[tw] OR Zemplen[tw] OR Budapest[tw] OR Csongrad[tw] OR Fejer[tw] OR gyor[tw] OR moson[tw] OR sopron[tw] OR hajdu[tw] OR bihar[tw] OR Heves[tw] OR "jasz nagykun szolnok"[tw] OR komarom[tw] OR esztergom[tw] OR Nograd[tw] OR (Pest[tw] AND (megye[tw] OR county[tw])) OR Somogy[tw] OR szabolcs[tw] OR szatmar[tw] OR bereg[tw] OR Tolna[tw] OR Vas[tw] OR Veszprem[tw] OR Zala[tw] OR Debrecen[tw] OR Miskolc[tw] OR Szeged[tw] OR Pecs[tw] OR Gyor[tw] OR Nyiregyhaza[tw] OR Kecskemet[tw] OR Szekesfehervar[tw] OR Szombathely[tw]) OR ("Ireland"[Mesh] OR Ireland[tw] OR Eire[tw] OR Irish*[tw] OR Fingal[tw] OR "Fine Gall"[tw] OR Dublin[tw] OR "Ath Cliath"[tw] OR "Dun Laoghaire"[tw] OR Wicklow[tw] OR "Cill Mhantain"[tw] OR "Chill Mhantain"[tw] OR Wexford[tw] OR "Loch Garman"[tw] OR Carlow[tw] OR Ceatharlach[tw] OR Kildare[tw] OR "Cill Dara"[tw] OR "Chill Dara"[tw] OR Meath[tw] OR "An Mhi"[tw] OR "Contae na Mi"[tw] OR Louth[tw] OR "Contae Lu"[tw] OR Monaghan[tw] OR Muineachan[tw] OR Mhuineachain[tw] OR Cavan[tw] OR "An Cabhan"[tw] OR "An Cabhain"[tw] OR Longford[tw] OR "An Longfort"[tw] OR "an Longfoirt"[tw] OR Langfurd[tw] OR Westmeath[tw] OR "An Iarmhi"[tw] OR "na Iarmhi"[tw] OR Offaly[tw] OR "Uibh Fhaili"[tw] OR Laois[tw] OR Laoise[tw] OR Kilkenny[tw] OR "Chill Chainnigh"[tw] OR "Cill Chainnigh"[tw] OR Waterford[tw] OR "Port Lairge"[tw] OR Watterford[tw] OR Cork[tw] OR Corcaigh[tw] OR Chorcai[tw] OR Kerry[tw] OR Ciarrai[tw] OR Chiarrai[tw] OR Limerick[tw] OR Luimneach[tw] OR Luimnigh[tw] OR Tipperary[tw] OR "Tiobraid Arann"[tw] OR "Thiobraid Arann"[tw] OR Clare[tw] OR "An Clar"[tw] OR "an Chlair"[tw] OR Galway[tw] OR Gaillimh[tw] OR "na Gaillimhe"[tw] OR Mayo[tw] OR "Maigh Eo"[tw] OR "Mhaigh Eo"[tw] OR Roscommon[tw] OR "Ros comain"[tw] OR Sligo[tw] OR Sligeach[tw] OR Shligigh[tw] OR Leitrim[tw] OR Liatroim[tw] OR Liatroma[tw] OR Donegal[tw] OR "Dhun na nGall"[tw] OR Dinnygal[tw] OR Dunnyga[tw] OR Leinster[tw] OR Laighin[tw] OR "Cuige Laighean"[tw] OR Munster[tw] OR Mumhain[tw] OR "Cuige Mumhan"[tw] OR Connacht[tw] OR Connachta[tw] OR Drogheda[tw] OR "Droichead Atha"[tw] OR Dundalk[tw] OR "Dun Dealgan"[tw] OR Swords[tw] OR Sord[tw] OR Bray[tw] OR Bre[tw] OR Navan[tw] OR "An Uaimh"[tw]) OR ("Italy"[Mesh] OR Italy[tw] OR Italia*[tw] OR Abruzzo[tw] OR Abruzzi[tw] OR Basilicata[tw] OR Lucania[tw] OR Calabria[tw] OR Campania[tw] OR "Emilia Romagna"[tw] OR "friuli venezia giulia"[tw] OR Lazio[tw] OR Latium[tw] OR Liguria*[tw] OR Lombardy[tw] OR Lombardia[tw] OR Marche[tw] OR Marches[tw] OR Molisano[tw] OR Molise[tw] OR Piedmont*[tw] OR Piemonte[tw] OR Bolzano[tw] OR Bozen[tw] OR Trentino[tw] OR Trento[tw] OR Puglia[tw] OR Apulia[tw] OR Sardinia[tw] OR Sardegna[tw] OR Sicily[tw] OR Sicilia[tw] OR Toscana[tw] OR Tuscany[tw] OR Umbria[tw] OR "Valle d Aosta"[tw] OR "Vallee d Aoste"[tw] OR "Valle	

	d'Aosta"[tw] OR "Vallee d'Aoste"[tw] OR "Aosta Valley"[tw] OR Veneto[tw] OR Venetia[tw] OR Triveneto[tw] OR Rome[tw] OR Roma[tw] OR Milan[tw] OR Milano[tw] OR Naples[tw] OR Napoli[tw] OR Turin[tw] OR Torino[tw] OR Palermo[tw] OR Genoa[tw] OR Genova[tw] OR Bologna[tw] OR Florence[tw] OR Firenze[tw] OR Bari[tw] OR Catania[tw]) OR ("Latvia"[Mesh] OR Latvi*[tw] OR Riga[tw] OR Courland[tw] OR Kurzeme[tw] OR Kurland[tw] OR Latgale[tw] OR Lettgallia[tw] OR Latgola[tw] OR Latgalia[tw] OR Vidzeme[tw] OR Vidumo[tw] OR Semigallia[tw] OR Semigalia[tw] OR Zemgale[tw] OR Pieriga[tw] OR Daugavpils[tw] OR Dinaburg[tw] OR Jekabpils[tw] OR Jakobstadt[tw] OR Jelgava[tw] OR Jurmala[tw] OR Liepaja[tw] OR Libau[tw] OR Rezekne[tw] OR Rezne[tw] OR Rositten[tw] OR Valmiera[tw] OR Wolmar[tw] OR Ventspils[tw] OR Windau[tw] OR Ogre[tw]) OR ("Lithuania"[Mesh] OR Lithuania*[tw] OR "Lietuvos Respublika"[tw] OR Lietuva[tw] OR lietuviu[tw] OR Alytus[tw] OR Alytaus[tw] OR Kaunas[tw] OR Kauno[tw] OR Klaipeda[tw] OR Klaipedos[tw] OR Marijampoles[tw] OR Marijampole[tw] OR Panevezys[tw] OR Panevezio[tw] OR Siauliai[tw] OR Siauliu[tw] OR Taurages[tw] OR Taurage[tw] OR Telsiu[tw] OR Telsiai[tw] OR Utenos[tw] OR Utena[tw] OR Vilnius[tw] OR Vilniaus[tw] OR Mazeikiai[tw] OR Jonava[tw] OR Mazeikiu[tw] OR Jonavos[tw]) OR ("Luxembourg"[Mesh] OR Luxembourg*[tw] OR Luxemburg[tw] OR Letzebuerg[tw] OR Diekirch[tw] OR Grevenmacher[tw] OR "Esch sur Alzette"[tw] OR "Esch Uelzecht"[tw] OR "Esch an der Alzette"[tw] OR "Esch an der Alzig"[tw] OR Dudelange[tw] OR Diddeleng[tw] OR Dudelingen[tw] OR Duedelingen[tw] OR Schifflange[tw] OR Scheffleng[tw] OR Schifflingen[tw] OR Bettembourg[tw] OR Beetebuerg[tw] OR Bettemburg[tw] OR Petange[tw] OR Peiteng[tw] OR Petingen[tw] OR Ettelbruck[tw] OR Ettelbreck[tw] OR Ettelbrueck[tw] OR Diekirch[tw] OR Dikrech[tw] OR Strassen[tw] OR Stroossen[tw] OR Bertrange[tw] OR Bartreng[tw] OR Bartringen[tw]) OR ("Malta"[Mesh] OR Malta[tw] OR Maltese*[tw] OR Maltin[tw] OR Gozo[tw] OR Ghawdex[tw] OR Valletta[tw] OR "Ill Belt"[tw] OR Birkirkara[tw] OR "B Kara"[tw] OR "B'Kara"[tw] OR Birchircara[tw] OR Mosta[tw] OR Qormi[tw] OR "St Paul s Bay"[tw] OR "St Paul's Bay"[tw] OR "Pawl il Bahar"[tw] OR Zabbar[tw] OR Sliema[tw] OR Naxxar[tw] OR Gwann[tw] OR "St John"[tw] OR Zebbug[tw] OR "Citta rohan"[tw] OR Fgura[tw]) OR ("Netherlands"[Mesh] OR Netherlands[tw] OR Nederland*[tw] OR Dutch*[tw] OR Drenthe[tw] OR Flevoland[tw] OR Friesland[tw] OR Fryslan[tw] OR Frisia[tw] OR Gelderland[tw] OR Guelders[tw] OR Groningen[tw] OR Limburg[tw] OR Brabant[tw] OR Holland[tw] OR Overijssel[tw] OR Overissel[tw] OR Utrecht[tw] OR Zeeland[tw] OR Amsterdam[tw] OR Rotterdam[tw] OR Hague[tw] OR "s-Gravenhage"[tw] OR "Den Haag"[tw] OR Eindhoven[tw] OR Tilburg[tw] OR Almere[tw] OR Breda[tw] OR Nijmegen[tw] OR Nimeguen[tw]) OR ("Poland"[Mesh] OR Poland[tw] OR Polska[tw] OR Polish[tw] OR Pole[tw] OR Poles[tw] OR Polski[tw] OR Polak[tw] OR Polka[tw] OR Polacy[tw] OR Dolnoslaskie[tw] OR Silesia*[tw] OR Slask[tw] OR Pomorskie[tw] OR Pomerania*[tw] OR Kujawsko[tw] OR Kuyavian[tw] OR Lodzkie[tw] OR Lodz[tw] OR Lubelskie[tw] OR Lublin[tw] OR Lubuskie[tw] OR Lubusz[tw] OR Lubus[tw] OR Malopolskie[tw] OR Mazowieckie[tw] OR Mazowske[tw] OR Masovia[tw] OR Masovian[tw] OR Opolskie[tw] OR Opole[tw] OR Podkarpackie[tw] OR Subcarpathian*[tw] OR Podlaskie[tw] OR Podlachia[tw] OR Podlasie[tw] OR Slaskie[tw] OR Swietokrzyskie[tw] OR "Varmia Mazuria"[tw] OR "Varmian Mazurian"[tw] OR "Varmia Masuria"[tw] OR "Varmian Masurian"[tw] OR "Warmia Mazury"[tw] OR "Warminsko Mazurskie"[tw] OR "Warmian Masurian"[tw] OR Wielkopolskie[tw] OR Zachodniopomorskie[tw] OR Warsaw[tw] OR Warszawa[tw] OR Krakow[tw] OR Cracow[tw] OR Wroclaw[tw] OR Poznan[tw] OR Gdansk[tw] OR Szczecin[tw] OR Bydgoszcz[tw] OR Katowice[tw]) OR ("Portugal"[Mesh] OR Portugal[tw] OR Portugues*[tw] OR Azores[tw] OR Acores[tw] OR Madeira[tw] OR Alentejo[tw] OR Algarve[tw] OR Lisboa[tw] OR Lisbon[tw] OR "Alto Tras-os-Montes"[tw] OR (Ave[tw] AND (community[tw] OR intermunicipal[tw] OR comunidade[tw])) OR Mondego[tw] OR Vouga[tw] OR Beira[tw] OR Cavado[tw] OR Lafoes[tw] OR Douro[tw] OR Porto[tw] OR Oporto[tw] OR Tejo[tw] OR Minho[tw] OR Setubal[tw] OR Pinhal[tw] OR "Serra da Estrela"[tw] OR Tamega[tw] OR Leira[tw] OR Santarem[tw] OR Beja[tw] OR Faro[tw] OR Evora[tw] OR Portalegre[tw] OR	

	"Castelo Branco"[tw] OR Guarda[tw] OR Cimbra[tw] OR Aveiro[tw] OR Viseu[tw] OR Braganca[tw] OR Braganza[tw] OR Braga[tw] OR "Vila real"[tw] OR "Viana do Castelo"[tw] OR Gaia[tw] OR Amadora[tw] OR Funchal[tw] OR Coimbra[tw] OR Almada[tw] OR (Agualva[tw] AND Cacem[tw])) OR ("Romania"[Mesh] OR Romania*[tw] OR Rumania*[tw] OR Roumania*[tw] OR Romani[tw] OR Rumani[tw] OR Alba[tw] OR Arad[tw] OR Arges[tw] OR Bacau[tw] OR Bihor[tw] OR "Bistrita Nasaud"[tw] OR Botosani[tw] OR Braila[tw] OR Brasov[tw] OR Kronstadt[tw] OR Brasso[tw] OR Brassovia[tw] OR Coron[tw] OR Bucharest[tw] OR Bucuresti[tw] OR Buzau[tw] OR Calarasi[tw] OR "Caras-Severin"[tw] OR Cluj[tw] OR Klausenburg[tw] OR Kolozsvar[tw] OR Constanta[tw] OR Tomis[tw] OR Konstantia[tw] OR Kostence[tw] OR Covasna[tw] OR Dambovita[tw] OR Dolj[tw] OR Galati[tw] OR Galatz[tw] OR Galac[tw] OR Kalas[tw] OR Giurgiu[tw] OR Gorj[tw] OR Harghita[tw] OR Hunedoara[tw] OR Ialomita[tw] OR Iasi[tw] OR Jassy[tw] OR Lassy[tw] OR Ilfov[tw] OR Maramures[tw] OR Mehedinti[tw] OR Mures[tw] OR Neamt[tw] OR (Olt[tw] AND (river[tw] OR county[tw] OR region[tw] OR judetul[tw] OR Raul[tw])) OR Prahova[tw] OR Salaj[tw] OR "Satu Mare"[tw] OR Sibiu[tw] OR Suceava[tw] OR Teleorman[tw] OR Timis[tw] OR Tulcea[tw] OR Valcea[tw] OR Vilcea[tw] OR Vaslui[tw] OR Vrancea[tw] OR Timisoara[tw] OR Temeswar[tw] OR Temeschburg[tw] OR Temeschwar[tw] OR Temesvar[tw] OR Temisvar[tw] OR Timisvar[tw] OR Temesva[tw] OR Craiova[tw] OR Ploiesti[tw] OR Ploesti[tw] OR Oradea[tw] OR Varad[tw] OR Varat[tw]) OR ("Slovakia"[Mesh] OR Slovakia[tw] OR Slovensk*[tw] OR Slovak*[tw] OR Slovaci[tw] OR Slovenki[tw] OR Bratislav*[tw] OR Presporok[tw] OR Pressburg[tw] OR Pressburg[tw] OR Posonium[tw] OR Banskobystri*[tw] OR "Banska Bystrica"[tw] OR Neusohl[tw] OR Besztercebanya[tw] OR Kosic*[tw] OR Kaschau[tw] OR Kassa[tw] OR Nitrian*[tw] OR Nitra[tw] OR Neutra[tw] OR Nyitra[tw] OR Nyitria[tw] OR Trnav*[tw] OR Tyrnau[tw] OR Nagyszombat[tw] OR Tyrnavia[tw] OR Presov*[tw] OR Trencian*[tw] OR Trencin[tw] OR Trentschin[tw] OR Trencsen[tw] OR Zilina[tw] OR Sillein[tw] OR Zsolna[tw] OR Zylina[tw] OR (Martin[tw] AND (city[tw] OR Svaty[tw])) OR Turocszentmarton[tw] OR Poprad[tw] OR Deutschendorf[tw] OR Zvolen[tw]) OR ("Slovenia"[Mesh] OR Slovenia*[tw] OR Slovenija[tw] OR slovensk*[tw] OR Slovenci[tw] OR Slovene*[tw] OR Gorenjska[tw] OR Carniola[tw] OR Goriska[tw] OR Gorizia[tw] OR Jugovzhodna[tw] OR Koroska[tw] OR Carinthia[tw] OR "Notranjsko kraska"[tw] OR "Obalno kraska"[tw] OR "Coastal karst"[tw] OR Osrednjeslovenska[tw] OR Podravska[tw] OR Drava[tw] OR Pomurska[tw] OR Mura[tw] OR Savinjska[tw] OR Savinja[tw] OR Spodnjeposavska[tw] OR Zasavska[tw] OR "Central Sava"[tw] OR Posavska[tw] OR "Lower Sava"[tw] OR Ljubljana[tw] OR Laibach[tw] OR Lubiana[tw] OR Maribor[tw] OR "Marburg an der Drau"[tw] OR Kranj[tw] OR Carnium[tw] OR Creina[tw] OR Chreina[tw] OR Krainbur[tw] OR Koper[tw] OR Capodistria[tw] OR Kopar[tw] OR Celje[tw] OR "Novo mesto"[tw] OR Neustadtl[tw] OR Domzale[tw] OR Velenje[tw] OR Wollan[tw] OR Woellan[tw] OR "Nova Gorica"[tw] OR Kamnik[tw]) OR ("Spain"[Mesh] OR Spain[tw] OR Espana[tw] OR Spanish[tw] OR Espanol*[tw] OR Spaniard*[tw] OR Andalucia[tw] OR Andalusia[tw] OR Aragon[tw] OR Arago[tw] OR Cantabria[tw] OR Canarias[tw] OR "Canary Islands"[tw] OR (Canaries[tw] AND island*[tw]) OR "Castile and leon"[tw] OR "Castilla y Leon"[tw] OR "Castile La Mancha"[tw] OR "Castilla La Mancha"[tw] OR Cataluna[tw] OR Catalonia[tw] OR Ceuta[tw] OR Madrid[tw] OR Melilla[tw] OR Navarra[tw] OR Navarre[tw] OR Valencia*[tw] OR Extremadura[tw] OR Galicia[tw] OR Balears[tw] OR "Balearic Islands"[tw] OR "Balear Islands"[tw] OR Baleares[tw] OR "La Rioja"[tw] OR "Pais Vasco"[tw] OR "Basque Country"[tw] OR "Baske region"[tw] OR Euskadi[tw] OR Asturias[tw] OR Murcia[tw] OR Coruna[tw] OR Alava[tw] OR Araba[tw] OR Albacete[tw] OR Alicante[tw] OR Alacant[tw] OR Almeria[tw] OR Avila[tw] OR Badajoz[tw] OR Badajos[tw] OR Barcelona[tw] OR Burgos[tw] OR Caceres[tw] OR Cadiz[tw] OR Castellon[tw] OR Castello[tw] OR "Ciudad Real"[tw] OR Cordoba[tw] OR Cuenca[tw] OR Eivissa[tw] OR Ibiza[tw] OR Formentera[tw] OR "El Hierro"[tw] OR Fuerteventura[tw] OR Galiza[tw] OR Girona[tw] OR Gerona[tw] OR "Gran Canaria"[tw] OR Granada[tw] OR Guadalajara[tw] OR Guipuzcoa[tw] OR Gipuzkoa[tw] OR Huelva[tw] OR Huesca[tw] OR Jaen[tw] OR "La	

	Gomera"[tw] OR "La Palma"[tw] OR Lanzarote[tw] OR Leon[tw] OR Lleida[tw] OR Lerida[tw] OR Lugo[tw] OR Malaga[tw] OR Mallorca[tw] OR Majorca[tw] OR Menorca[tw] OR Minorca[tw] OR Murcia[tw] OR Ourense[tw] OR Orense[tw] OR Palencia[tw] OR Pontevedra[tw] OR Salamanca[tw] OR Segovia[tw] OR Sevilla[tw] OR Seville[tw] OR Soria[tw] OR Tarragona[tw] OR Tenerife[tw] OR Teruel[tw] OR Toledo[tw] OR Valladolid[tw] OR Vizcaya[tw] OR Biscay[tw] OR Zamora[tw] OR Zaragoza[tw] OR Saragossa[tw] OR "Las Palmas"[tw] OR Bilbao[tw] OR Bilbo[tw]) OR ("Sweden"[Mesh] OR Sweden[tw] OR Sverige[tw] OR Swedish[tw] OR Svenska[tw] OR svenskar[tw] OR Swede[tw] OR Swedes[tw] OR Norrland[tw] OR Mellansverige[tw] OR Smaland[tw] OR Stockholm*[tw] OR Sydsverige[tw] OR Vastsverige[tw] OR Blekinge[tw] OR Dalarna[tw] OR Gavleborg*[tw] OR Gotland*[tw] OR Halland*[tw] OR Jamtland*[tw] OR Jonkoping*[tw] OR Kalmar[tw] OR Kronoberg*[tw] OR Norrbotten*[tw] OR Orebro[tw] OR Ostergotland*[tw] OR Skane[tw] OR Sodermanlands[tw] OR Uppsala[tw] OR Varmland*[tw] OR Vasterbotten*[tw] OR Vasternorrland*[tw] OR Vastmanland*[tw] OR vastergotland*[tw] OR Gotaland*[tw] OR Gothenburg[tw] OR Goteborg[tw] OR Malmo[tw] OR Vasteras[tw] OR Linkoping[tw] OR Helsingborg[tw] OR Halsingborg[tw] OR Norrkoping[tw]) OR ("Great Britain"[Mesh] OR GB[tw] OR "United kingdom"[tw] OR UK[tw] OR Britain[tw] OR British[tw] OR England[tw] OR English[tw] OR Scotland[tw] OR Scottish[tw] OR Scots[tw] OR Wales[tw] OR Cymru[tw] OR Welsh[tw] OR "North Ireland"[tw] OR "Northern Ireland"[tw] OR Irish[tw] OR Avon[tw] OR Bedfordshire[tw] OR Berkshire[tw] OR Bristol[tw] OR Buckinghamshire[tw] OR Cambridgeshire[tw] OR "Isle of Ely"[tw] OR Cheshire[tw] OR Cleveland[tw] OR Cornwall[tw] OR Cumberland[tw] OR Cumbria[tw] OR Derbyshire[tw] OR Devon[tw] OR Dorset[tw] OR Durham[tw] OR Essex[tw] OR Gloucestershire[tw] OR Hampshire[tw] OR Southampton[tw] OR (Hereford[tw] AND Worcester[tw]) OR Hertfordshire[tw] OR Herefordshire[tw] OR Humberside[tw] OR Huntingdon[tw] OR Huntingdonshire[tw] OR "Isle of Wight"[tw] OR Kent[tw] OR Lancashire[tw] OR Leicestershire[tw] OR Lincolnshire[tw] OR London[tw] OR Manchester[tw] OR Merseyside[tw] OR Middlesex[tw] OR Norfolk[tw] OR Northamptonshire[tw] OR Northumberland[tw] OR Nottinghamshire[tw] OR Oxfordshire[tw] OR Peterborough[tw] OR Rutland[tw] OR Shropshire[tw] OR Salop[tw] OR Somerset[tw] OR Yorkshire[tw] OR Staffordshire[tw] OR Suffolk[tw] OR Surrey[tw] OR Sussex[tw] OR (Tyne[tw] AND Wear[tw]) OR Warwickshire[tw] OR Midlands[tw] OR Westmorland[tw] OR Wiltshire[tw] OR Worcestershire[tw] OR "Isle of Man"[tw] OR Jersey[tw] OR Guernsey[tw] OR "Channel Islands"[tw] OR Aberdeen[tw] OR Aberdeenshire[tw] OR Angus[tw] OR Forfarshire[tw] OR Argyll[tw] OR Ayrshire[tw] OR Banffshire[tw] OR Berwickshire[tw] OR Bute[tw] OR Caithness[tw] OR Clackmannanshire[tw] OR Cromartyshire[tw] OR Dumfriesshire[tw] OR Dunbartonshire[tw] OR Dumbarton[tw] OR Dundee[tw] OR Lothian[tw] OR Haddingtonshire[tw] OR Edinburgh[tw] OR Fife[tw] OR Glasgow[tw] OR Inverness-shire[tw] OR Kincardineshire[tw] OR Kinross-shire[tw] OR Kirkcudbrightshire[tw] OR Lanarkshire[tw] OR Midlothian[tw] OR Moray[tw] OR Elginshire[tw] OR Nairnshire[tw] OR Orkney[tw] OR Peeblesshire[tw] OR Perthshire[tw] OR Renfrewshire[tw] OR (Ross[tw] AND Cromarty[tw]) OR Ross-shire[tw] OR Roxburghshire[tw] OR Selkirkshire[tw] OR Shetland[tw] OR Zetland[tw] OR Stirlingshire[tw] OR Sutherland[tw] OR Linlithgowshire[tw] OR Wigtownshire[tw] OR Anglesey[tw] OR Brecknockshire[tw] OR Caernarfonshire[tw] OR Carmarthenshire[tw] OR Cardiganshire[tw] OR Ceredigion[tw] OR Clwyd[tw] OR Denbighshire[tw] OR Dyfed[tw] OR Flintshire[tw] OR Glamorgan[tw] OR Gwent[tw] OR Gwynedd[tw] OR Merionethshire[tw] OR Montgomeryshire[tw] OR Monmouthshire[tw] OR Pembrokeshire[tw] OR Powys[tw] OR Radnorshire[tw] OR Antrim[tw] OR Aontroim[tw] OR "Contae Aontroma"[tw] OR Anthrim[tw] OR Antrim[tw] OR Entrim[tw] OR Armagh[tw] OR "Ard Mhacha"[tw] OR Airmagh[tw] OR Belfast[tw] OR (Down[tw] AND (district[tw] OR council[tw] OR County[tw])) OR "An Dun"[tw] OR "an Duin"[tw] OR Doon[tw] OR Doun[tw] OR Fermanagh[tw] OR "Fear Manach"[tw] OR "Fhear Manach"[tw] OR Fermanay[tw] OR Londonderry[tw] OR	

	Doire[tw] OR Dhoire[tw] OR Lunnonderrie[tw] OR Derry[tw] OR Birmingham[tw] OR Leeds[tw] OR Sheffield[tw] OR Bradford[tw] OR Liverpool[tw])	
#19	Search (#14 OR #15 OR #16 OR #17 OR #18)	9687092
#20	Search (#5 AND #13 AND #19)	44324
#21	Search (letter[PT] OR news[PT] OR editorial[PT] OR congresses[PT])	1718845
#22	Search (#20 NOT #21)	11913
#23	Search (#22 AND 2009:2019[DP])	5197

Supplementary Table S1b. Search string and results – Embase

Search	Terms	Results
1	exp prevalence/	678086
2	exp seroepidemiology/	3765
3	exp disease surveillance/	25720
4	"seroepidemiolog*".ab,ti.	4388
5	"epidemiolog*".ab,ti.	426775
6	"surveillance*".ab,ti.	217248
7	"serolog*".ab,ti.	128698
8	"serosurvey*".ab,ti.	1477
9	"seroprevalen*".ab,ti.	19710
10	"seropositiv*".ab,ti.	44457
11	"prevalence*".ab,ti.	803053
12	(sero adj2 (survey* or epidemiolog* or prevalen* or positiv*)).ab,ti.	3631
13	1 or 2 or 3 or 4 or 5 or 6 or 7 or 8 or 9 or 10 or 11 or 12	1595973
14	exp Human immunodeficiency virus/	186485
15	exp Human immunodeficiency virus infection/	362799
16	exp acute HIV infection/	397
17	HIV.ab,ti.	373322

18	(human adj3 (immune* or immuno*) adj3 virus*).ab,ti.	92689
19	14 or 15 or 16 or 17 or 18	519379
20	13 and 19	97143
21	exp Human immunodeficiency virus prevalence/	9790
22	((prevalen* or seroprevalen* or 'sero prevalen*' or serodiagnos* or serolog* or diagnos* or screen*) adj10 hiv).ab,ti.	67791
23	((prevalen* or seroprevalen* or 'sero prevalen*' or serodiagnos* or serolog* or diagnos* or screen*) adj10 (human adj3 (immune* or immuno*) adj3 virus*)).ab,ti.	7398
24	21 or 22 or 23	73332
25	20 or 24	124319
26	exp migrant/	33086
27	exp migration/	40913
28	exp minority group/	13561
29	exp ethnic group/	126525
30	exp ethnicity/	68822
31	"emigrant*".ab,ti.	1587
32	(born adj abroad).ab,ti.	299
33	(born adj outside).ab,ti.	1331
34	"immigrant*".ab,ti.	25742
35	"migrant*".ab,ti.	16586
36	"refugee*".ab,ti.	10155
37	"alien*".ab,ti.	7378
38	"migrat*".ab,ti.	351156
39	"emigrat*".ab,ti.	6515
40	"immigrat*".ab,ti.	12902
41	"minorit*".ab,ti.	82176
42	"asylum*".ab,ti.	3666
43	"displaced*".ab,ti.	34986
44	"foreign*".ab,ti.	80501
45	"origin*".ab,ti.	864611
46	nationality.ab,ti.	5780

47	minority.ab,ti.	72890
48	minorities.ab,ti.	13393
49	"resettlement*".ab,ti.	1213
50	(foreign* adj2 born*).ab,ti.	3723
51	26 or 27 or 28 or 29 or 30 or 31 or 32 or 33 or 34 or 35 or 36 or 37 or 38 or 39 or 40 or 41 or 42 or 43 or 44 or 45 or 46 or 47 or 48 or 49 or 50	1611188
52	exp prisoner/	14888
53	exp prison/	13646
54	exp detention camp/	220
55	"incarcerat*".ab,ti.	12339
56	"inmate*".ab,ti.	5347
57	"prisoner*".ab,ti.	8083
58	confinement.ab,ti.	11181
59	"gaol*".ab,ti.	138
60	"jail*".ab,ti.	3770
61	"penitentiar*".ab,ti.	654
62	"prison*".ab,ti.	16708
63	"reformator*".ab,ti.	89
64	custodial.ab,ti.	1073
65	custody.ab,ti.	3009
66	custodies.ab,ti.	14
67	"remand*".ab,ti.	454
68	"detention*".ab,ti.	3752
69	"detainee*".ab,ti.	1005
70	"imprison*".ab,ti.	2678
71	"cellmate*".ab,ti.	26
72	"convict*".ab,ti.	7466
73	offender.ab,ti.	4150
74	offenders.ab,ti.	11366
75	((correctional* or penal or internment or closed) adj5 (facilit* or institution* or camp* or setting*)).ab,ti.	3428

76	52 or 53 or 54 or 55 or 56 or 57 or 58 or 59 or 60 or 61 or 62 or 63 or 64 or 65 or 66 or 67 or 68 or 69 or 70 or 71 or 72 or 73 or 74 or 75	72154
77	exp men who have sex with men/	8764
78	exp LGBT people/	8238
79	exp bisexual male/	1090
80	exp homosexual male/	1889
81	exp male homosexuality/	2733
82	MSM.ab,ti.	13355
83	MASM.ab,ti.	20
84	MSMW.ab,ti.	123
85	GBMSM.ab,ti.	69
86	GB-MSM.ab,ti.	8
87	"cruisin*".ab,ti.	521
88	"queer*".ab,ti.	1055
89	((men or man or male or males) adj3 (who or has or have or having or had or report* or inform*) adj3 sex* adj3 (men or man or male or males)).ab,ti.	18597
90	("male to male" adj3 sex* adj3 contact).ab,ti.	91
91	((gay* or homosexual* or bisexual* or 'non heterosexual*' or 'non binary' or lesbigay* or lbg) adj5 (men or man or male or males)).ab,ti.	12680
92	77 or 78 or 79 or 80 or 81 or 82 or 83 or 84 or 85 or 86 or 87 or 88 or 89 or 90 or 91	41897
93	exp transgender/	4771
94	exp transsexualism/	3467
95	exp transsexuality/	267
96	exp LGBT people/	8238
97	"transgender*".ab,ti.	5977
98	transpeople.ab,ti.	17
99	"transperson*".ab,ti.	257
100	"tran*sex*".ab,ti.	2846
101	"transm*n".ab,ti.	365065
102	"transwom*n".ab,ti.	220
103	"two spirit person*".ab,ti.	3

104	"two spirit individual*".ab,ti.	9
105	two spirit people.ab,ti.	5
106	glbt.ab,ti.	68
107	glbtq.ab,ti.	11
108	glbtqq.ab,ti.	1
109	lgbt.ab,ti.	1284
110	lgbtq.ab,ti.	499
111	lgbtqq.ab,ti.	1
112	(trans adj2 (man or men or male or males or 'wom*n' or female* or gender* or person* or people or individual* or sex*)).ab,ti.	973
113	((gender* or sex*) adj3 (variant* or reassign* or dissident*)).ab,ti.	1975
114	(intersex adj3 (individual* or people or person*)).ab,ti.	153
115	93 or 94 or 95 or 96 or 97 or 98 or 99 or 100 or 101 or 102 or 103 or 104 or 105 or 106 or 107 or 108 or 109 or 110 or 111 or 112 or 113 or 114	379995
116	exp prostitution/	8531
117	exp sex worker/	1455
118	exp transactional sex/	397
119	(sex* adj3 (transactional or work* or industr* or exchange or survival or paid)).ab,ti.	13540
120	"prostitut*".ab,ti.	3014
121	fsw.ab,ti.	1144
122	msw.ab,ti.	3196
123	csw.ab,ti.	497
124	"whore*".ab,ti.	196
125	"call girl*".ab,ti.	9
126	"streetwalker*".ab,ti.	7
127	"nightwalker*".ab,ti.	0
128	"lad* of the night*".ab,ti.	10
129	"walk the pavement*".ab,ti.	1
130	116 or 117 or 118 or 119 or 120 or 121 or 122 or 123 or 124 or 125 or 126 or 127 or 128 or 129	22647
131	exp pregnancy/	570841
132	exp pregnant woman/	70071

133	exp prenatal care/	136564
134	exp prenatal screening/	8208
135	"pregnant*".ab,ti.	210931
136	"pregnanc*".ab,ti.	471775
137	"gravid*".ab,ti.	15852
138	"child bearing".ab,ti.	3838
139	childbearing.ab,ti.	14824
140	"antenatal*".ab,ti.	47384
141	"ante natal* ".ab,ti.	711
142	131 or 132 or 133 or 134 or 135 or 136 or 137 or 138 or 139 or 140 or 141	849205
143	exp intravenous drug abuse/	10109
144	exp injection drug user/	811
145	PWID.ab,ti.	1848
146	IDU.ab,ti.	3770
147	IVDU.ab,ti.	977
148	((inject* or intraven* or parenteral or use* or misuse*) adj3 (drug* or substance*)).ab,ti.	279610
149	143 or 144 or 145 or 146 or 147 or 148	283717
150	51 or 76 or 92 or 115 or 130 or 142 or 149	3076974
151	exp Europe/	1420454
152	exp European Union/	25692
153	exp Eastern Europe/	189081
154	exp Western Europe/	946306
155	exp Southern Europe/	225212
156	exp Northern European/	8245
157	exp Eastern European/	5010
158	exp Central European/	12782
159	exp EU citizen/	30267
160	exp European/	169737
161	exp Western European/	14361
162	exp Southern European/	10563

163	exp Baltic States/	8803
164	exp Scandinavia/	183335
165	exp Czechoslovakia/	23049
166	exp "Yugoslavia (pre-1992)"/ or exp Yugoslavia/	27366
167	exp Balkan Peninsula/	272
168	exp Benelux/	65
169	(Europe* or europa* or eu or eea or efta or "eu/eea" or "eu/efta" or ecsc or euratom or eurozone or eec or ec or (schengen and (area or countr* or region* or state or states)) or euroregion or euroregions).ab,in,ti.	662339
170	(balkan or balkans or baltic or (mediterranean and (area or countr* or region* or state or states)) or (alpine and (area or countr* or region* or state or states)) or scandinavia or scandinavian or (nordic and (countr* or state*)) or danubian or "iberian peninsula" or "peninsula iberica" or "peninsule iberique" or "iberiar penintsula" or iberia or anatolia or anadolu or anatole or anatolian or yugoslavia or czechoslovakia or "czecho slovakia" or ceskoslovensko or "cesko slovensko" or benelux or fennoscandia or "fenno Scandinavia" or fennoskandi* or (visegrad and (group or four or triangle)) or "visegradska ctyrka" or "visegradska skupina" or "visegradi egyuttmukodes" or "segradi negyek" or "grupa wyszehradzka" or "vysehradska skupina" or "vysehradska stvorka").ab,in,ti.	102671
171	exp Austria/	19357
172	exp Austrian/	949
173	(austria* or osterreich* or oesterreich* or ostosterreich or ostoesterreich or sudosterreich or sudoesterreich or westosterreich or westoesterreich or burgenland or carinthia or karnten or kaernten or niederosterreich or niederoesterreich or oberosterreich or oberoesterreich or salzburg or saizburg or styria or steiermark or tyrol or tirol or vorarlberg or vienna or wien or graz or linz or innsbruck or klagenfurt or villach or wels or 'st polten' or 'st poelten' or 'sankt polten' or 'sankt poelten' or dornbirn).ab,in,ti.	275622
174	exp Belgium/	22499
175	exp Belgian/	959
176	(belgi* or belge* or belg or brussel* or bruxelles or bruxelloise or walloon* or wallon* or vlaams or flander* or flandern or flandre or flemish or flamand or flemisch or flamisch* or vlaanderen or flamande or waals or antwerp* or anvers or henegouwen or hennegau or hainault or hainaut or liege or luik or luttich or limbourg or limburg or namur or namen or ostflandern or westflandern or ghent or gent or gand or charleroi or bruges or brugge* or schaerbeek or schaarbeek or anderlecht or leuven or louvain).ab,in,ti.	413077
177	exp Bulgaria/	6899
178	exp "Bulgarian (people)"/ or exp "Bulgarian (citizen)"/	323
179	(bulgaria or balgariya or balgarija or blagoevgrad* or 'pirin macedonia' or burgas or dobrich or gabrovo or haskovo or kardzhali or kurdzhali or kyustendil or lovech or lovec or montana or pazardzhik or pernik or pleven* or plovdiv or razgrad or rousse or ruse or rusenka or shumen or silistra or sliven or smolyan or sofia or sofyiska or sofiiska or 'stara zagora' or targovishte or varna or 'veliko tarnovo' or vidin or vratsa or vratza or yambol).ab,in,ti.	77303
180	exp Croatia/	9528

181	exp "Croatian (citizen)"/	319
182	"Croat (people)"/	4
183	(croat* or hrvatsk* or hrvati or bjelovar or 'bjelovarsko bilogorska' or 'brod posavina' or 'brodsko posavska' or 'dubrovnik neretva' or 'dubrovacko neretvanska' or zagreb or zagrebacka or istria or istarska or karlovacka or karlovac or 'koprivnicko krizevacka' or koprivnica or krizevci or 'krapina zagorje' or 'krapinsko zagorska' or 'lika senj' or 'licko senjska' or medimurska or medimurje or osijek or baranja or 'osjecko baranjska' or 'pozega slavonia' or 'pozesko slavonska' or 'primorje gorski kotar' or 'primorsko goranska' or 'sibensko kninska' or 'sibensko kninske' or sibenik or knin or sisak or 'sisacko moslavacka' or moslavina or 'splitsko dalmatinska' or split or dalmatia or varazdin or varazdinska or viroviticko or podravska or virovitica or podravina or 'vukovarsko srijemska' or vukovar or srijem or zadar or zadarska or rijeka or 'velika gorica' or 'slavonski brod' or pula).ab,in,ti.	125312
184	exp Cyprus/	2058
185	exp Cypriot/	157
186	(cyprus or cypriot* or kypros or kibris* or kypriaki or kyprioi or nicosia or lefkosa or lefkosia or famagusta or magusa or ammochostos or gazimagusa or kyrenia or girne or keryneia or larnaca or larnaka or iskele or limassol or lemesos or limasol or leymosun or paphos or pafos or baf or strovolos or lakatamia or lakadamya or 'kato polemidia' or 'kato polemidhia' or aglandjia or eglence or aglantzia or aradhippou or aradippou or engomi).ab,in,ti.	13775
187	exp "Czech (people)"/ or exp "Czech (citizen)"/ or exp Czech Republic/	13418
188	(czech* or cesky or ceska or cech or cestina or prague or praha or prag or stredoces* or jihoces* or bohemia or bohemian or plzen* or pilsen or karlovars* or 'karlovy vary' or usteck* or usti or liberec* or 'hradec kralove' or kralovehradec* or pardubic* or olomouc* or olomoc or holomoc or moravskoslezs* or jihomorav* or moravia or moravian or morava or vysocina or zlin or zlinsk* or 'ceske budejovice' or budweis or brno or ostrava).ab,in,ti.	175368
189	exp Denmark/	44083
190	exp Danish citizen/	1963
191	exp "Dane (people)"/	133
192	(denmark or danish* or dane or danes or danmark or dansk* or hovedstaden or midtjylland or nordjylland or sjaelland or sealand or 'zealand region' or 'region zealand' or syddanmark or jutland or jylland or sonderjyllands or copenhagen or kobenhavn or arhus or aarhus or bornholm or frederiksberg or frederiksborg or ringkjobing or viborg or vejle or roskilde or storstrom or vestsjaellands or 'west zealand' or funen or ribe or 'kalaallit nunaat' or gronland or foroyar or faeroerne or 'faroe islands' or aalborg or alborg or odense or esbjerg or gentofte or gladsaxe or randers or kolding).ab,in,ti.	331258
193	exp Estonia/	3576
194	exp "Estonian (people)"/ or exp "Estonian (citizen)"/	157
195	(estonia* or eesti or eestlased or eestlane or harju or harjumaa or hiiu or hiiumaa or 'ida viru' or 'ida virumaa' or jarvamaa or jarva or jogevamaa or jogeva or laanemaa or laane or 'laane virumaa' or parnu or parnumaa or polva or polvamaa or rapla or raplamaa or saare or saaremaa or tartu or tartumaa or valga or valgamaa or viljandimaa or viljandi or voru or vorumaa or tallinn or narva or 'kohtla jarve' or rakvere or maardu or sillamae or kuressaare).ab,in,ti.	17908
196	exp Finland/	32673
197	exp "Finn (people)"/ or exp "Finn (citizen)"/	1165

198	(finland or finnish* or finn or finns or suomi or suomen or suomalaiset or aland or ahvenanmaa or uusimaa or nyland or karelia or karjala or karelen or ostrobothnia or pohjanmaa or osterbotten or savonia or savo or savolax or kainuu or kajanaland* or 'kanta hame' or tavastia or tavastland or kymenlaakso or kymmenedalen or lapland or lappi or lappland or 'paijat hame' or pirkanmaa or birkaland or satakunta or satakunda or helsinki or helsingfors or espoo or esbo or tampere or tammerfors or vantaa or vanda or oulu or uleaborg or turku or abo or jyvaskyla or kuopio or lahti or lahtis or kouvola).ab,in,ti.	234593
199	exp France/	108014
200	exp Frenchman/	1694
201	(france or french* or francais* or alsace or elsass or aquitaine or aquitania or akitania or aguiene or auvergne or auvernhe or auvernha or normandie or normandy or normaundie or bourgogne or burgundy or bregogne or borgoegne or borgogne or brittany or breizh or bertaeyn or bretagne or 'champagne ardenne' or corse or corsica or 'franche comte' or 'frantche comte' or 'franche comtat' or guadeloupe or guyane or guiana or 'languedoc roussillon' or 'lengadoc rosselhon' or 'llenguadoc-rossello' or limousin or lemosin or lorraine or lothringen or lottringe or martinique or 'midi pyrenees' or 'miegjorn pireneus' or 'mieidia pireneus' or 'mediodia pirineos' or 'pays de la loire' or 'broiou al liger' or picardie or picardy or 'poitou charentes' or 'peitau charantas' or 'poetou-cherentes' or provence or provenca or prouvenco or 'cote d azur' or 'costo d azur' or 'costa d azur' or reunion or 'rhone alpes' or 'rono arpes' or 'rose aups' or ain or aisne or allier or 'alpes de haute provence' or 'haute alpes' or 'alpes maritimes' or ardeche or ardennes or ariege or aube or aude or aveyron or 'bas rhin' or 'bouches du rhone' or calvados or cantal or charente or cher or correze or 'corse du sud' or 'cote d or' or 'cotes d armor' or creuse or 'deux sevres' or dordogne or doubs or drome or essonne or eure or finistere or gard or gers or gironde or 'haute corse' or 'haute garonne' or 'haute marne' or 'hautes alpes' or 'haute saone' or 'haute savoie' or 'hautes pyrenees' or 'haute vienne' or 'haut rhin' or 'hauts de seine' or herault or 'ile de france' or 'ille et vilaine' or indre or isere or jura or landes or loire or loiret or (lot adj3 (departement or department)) or 'lot et garonne' or 'loir et cher' or lozere or manche or marne or mayenne or mayotte or 'meurthe et moselle' or meuse or morbihan or moselle or (nord adj3 (department or departement)) or nievre or oise or orne or 'pas de calais' or 'noord-nauw van kales' or paris or 'puy de dome' or 'pyrenees atlantiques' or 'pyrenees orientales' or rhone or sarthe or savoie or 'seine et marne' or 'seine maritime' or somme or tarn or 'territoire de belfort' or 'val de marne' or 'val d oise' or var or vaucluse or vendee or vienne or vosges or yonne or yvelines or marseille or lyon or nice or nantes or strasbourg or montpellier or bordeaux or lille or toulouse or 'outre mer' or 'seine saint denis').ab,in,ti.	1490327
202	exp Germany/	160356
203	exp "German (citizen)"/	6237
204	(german* or deutsch* or bundesrepublik or westdeutschland or ostdeutschland or baden or wuerttemberg or wurttemberg or bayern or bavaria or berlin or brandenburg or bremen or oldenburg or mitteldeutschland or rhein or rhine or hannover or braunschweig or gottingen or goettingen or nurnberg or nuernberg or ruhr or koln or koeln or bonn or hamburg or hessen or hesse or hessia or mecklenburg or vorpommern or pomerania or niedersachsen or neddersassen or saxony or niederbayern or 'northern rhine' or 'north rhine' or westphalia or westfalen or 'rhineland palatinate' or 'rheinland pfalz' or saarland or sachsen or 'schleswig holstein' or thuringia or thuringen or thueringen or munchen or muenchen or munich or frankfurt or stuttgart or dusseldorf or duesseldorf or dortmund or essen).ab,in,ti.	2018019
205	exp Greece/	24175
206	exp "Greek (citizen)"/	691
207	exp "Greek (people)"/	313

208	(greece or 'hellenic republic' or greek* or ellada or elladas or 'elliniki dimokratia' or hellas or hellenes or attica or attiki or makedonia* or macedonia or thraki or thrace or crete or kriti or 'ionia nisia' or 'ionion neson' or 'ionion nison' or 'ionian islands' or 'ionian island' or epirus or ipeiros or 'perifereia ipeirou' or 'north aegean' or 'northern aegean' or 'aegean islands' or 'aegean island' or 'nisoi agaiou' or 'notio aigaio' or peloponnese or peloponniso* or thessaly or thessalia or thessalian or petthalia or 'voreio aigaio' or 'voreio aigaiou' or 'south aegean' or 'southern aegean' or 'mount athos' or 'oros athos' or cyclades or cycklades or kiklades or dodecanese or dodekanisa or athens or athina or thessaloniki or thessalonica or patras or patra or pireas or piraeus or larissa or larisa or heraklion or heraclion or iraklion or irakleion or iraklio or volos or rhodes or rodos or ioannina or janina or yannena or chania or chalcis or chalkida).ab,in,ti.	249296
209	exp Hungary/	17340
210	exp "Hungarian (citizen)"/	919
211	exp "Magyar (people)"/	82
212	(hungar* or magyarorszag or magyar* or dunantuli or transdanubia or dunantul or 'great plain' or 'eszak alfold' or 'del alfold' or 'alfold es eszak' or 'northern alfold' or 'north alfold' or 'south alfold' or 'southern alfold' or bacs or kiskun or baranya or bekes or borsod or abauj or zemplen or budapest or csongrad or fejer or moson or sopron or hajdu or bihar or heves or 'jasz nagykun szolnok' or komarom or esztergom or nograd or (pest adj3 (megye or county)) or somogy or szabolcs or szatmar or bereg or tolna or vas or veszprem or zala or debrecen or miskolc or szeged or pecs or gyor or nyiregyhaza or kecskemet or szekesfehervar or szombathely).ab,in,ti.	207584
213	exp Ireland/	32125
214	exp "Irish (citizen)"/	959
215	(ireland or eire or irish* or fingal or 'fine gall' or dublin or 'ath cliath' or 'dun laoghaire' or wicklow or 'cill mhantain' or 'chill mhantain' or wexford or 'loch garman' or carlow or ceatharlach or kildare or 'cill dara' or 'chill dara' or meath or 'an mhi' or 'contae na mi' or louth or 'contae lu' or monaghan or muineachan or mhuineachain or cavan or 'an cabhan' or 'an cabhain' or longford or 'an longfort' or 'an longfoirt' or langfurd or westmeath or 'an iarmhi' or 'na iarmhi' or offaly or 'uibh fhaili' or laois or laoise or kilkenny or 'chill chainnigh' or 'cill chainnigh' or waterford or 'port lairge' or watterford or cork or corcaigh or chorcai or kerry or ciarrai or chiarrai or limerick or luimneach or luimnigh or tipperary or 'tiobraid arann' or 'thiobraid arann' or clare or 'an clar' or 'an chlair' or galway or gaillimh or 'na gaillimhe' or mayo or 'maigh eo' or 'mhaigh eo' or roscommon or 'ros comain' or sligo or sligeach or shligigh or leitrim or liatroim or liatroma or donegal or 'dhun na ngall' or dinnygal or dunnyga or leinster or laighin or 'cuige laighean' or munster or mumhain or 'cuige mumhan' or connacht or connachta or drogheda or 'droichead atha' or dundalk or 'dun dealgan' or swords or sord or bray or bre or navan or 'an uaimh').ab,in,ti.	554872
216	exp Italy/	101255
217	exp "Italian (citizen)"/	3886
218	exp Italic people/	3
219	(italy or italia* or abruzzo or abruzzi or basilicata or lucania or calabria or campania or 'emilia romagna' or 'friuli venezia giulia' or lazio or latium or liguria* or lombardy or lombardia or marche or marches or molisano or molise or piedmont* or piemonte or bolzano or bozen or trentino or trento or puglia or apulia or sardinia or sardegna or sicily or sicilia or toscana or tuscany or umbria or 'valle d aosta' or 'vallee d aoste' or 'aosta valley' or veneto or venetia or triveneto or rome or roma or milan or milano or naples or napoli or turin or torino or palermo or genoa or genova or bologna or florence or firenze or bari or catania).ab,in,ti.	1298554
220	exp Latvia/	2051

221	exp "Latvian (citizen)"/	68
222	exp "Lett (people)"/	17
223	(latvi* or riga or courland or kurzeme or kurland or latgale or lettgallia or latgola or latgalia or vidzeme or vidumo or semigallia or semigalia or zemgale or pieriga or daugavpils or dinaburg or jekabpils or jakobstadt or jelgava or jurmala or liepaja or libau or rezekne or rezne or rositten or valmiera or wolmar or ventspils or windau or ogre).ab,in,ti.	9710
224	exp Lithuania/	3737
225	exp "Lithuanian (people)"/ or exp "Lithuanian (citizen)"/	138
226	(lithuania* or 'lietuvos respublika' or lietuva or lietuviu or alytus or alytaus or kaunas or kauno or klaipeda or klaipedos or marijampoles or marijampole or panevezys or panevezio or siauliai or siauliu or taurages or taurage or telsiu or telsiai or utenos or utena or vilnius or vilniaus or mazeikiai or jonava or mazeikiu or jonavos).ab,in,ti.	17324
227	exp Luxembourg/	1166
228	(luxembourg* or luxemburg or letzebuerg or grevenmacher or 'esch sur alzette' or 'esch uelzecht' or 'esch an der alzette' or 'esch an der alzig' or dudelange or diddeleng or dudelingen or duedelingen or schifflange or scheffleng or schifflingen or bettembourg or beetebuerg or bettemburg or petange or peiteng or petingen or ettelbruck or ettelbreck or ettelbrueck or diekirch or dikrech or strassen or stroossen or bertrange or bartreng or bartringen).ab,in,ti.	10592
229	exp Malta/	1448
230	exp "Maltese (citizen)"/	109
231	(malta or maltese* or maltin or gozo or ghawdex or valletta or 'ill belt' or birkirkara or 'b kara' or birchircara or mosta or qormi or 'st paul s bay' or 'pawl il bahar' or zabbar or sliema or naxxar or gwann or 'st john' or zebbug or 'citta rohan' or fgura).ab,in,ti.	17697
232	exp Netherlands/	73365
233	exp Dutchman/	1694
234	(netherlands or nederland* or dutch* or drenthe or flevoland or friesland or fryslan or frisia or gelderland or guelders or groningen or limburg or brabant or holland or overijssel or overissel or utrecht or zeeland or amsterdam or rotterdam or hague or 's-gravenhage' or 'den haag' or eindhoven or tilburg or almere or breda or nijmegen or nimeguen).ab,in,ti.	777122
235	exp Poland/	43036
236	exp Polish citizen/	1244
237	exp "Pole (people)"/	88
238	(poland or polska or polish or pole or poles or polski or polak or polka or polacy or dolnoslaskie or silesia* or slask or pomorskie or pomerania* or kujawsko or kuyavian or lodzkie or lodz or lubelskie or lublin or lubuskie or lubusz or lubus or malopolskie or mazowieckie or mazowske or masovia or masovian or opolskie or opole or podkarpackie or subcarpathian* or podlaskie or podlachia or podlasie or slaskie or swietokrzyskie or 'varmia mazuria' or 'varmian mazurian' or 'varmia masuria' or 'varmian masurian' or 'warmia mazury' or 'warminsko mazurskie' or 'warmian masurian' or wielkopolskie or zachodniopomorskie or warsaw or warszawa or krakow or cracow or wroclaw or poznan or gdansk or szczecin or bydgoszcz or katowice).ab,in,ti.	379870
239	exp Portugal/	17730

240	exp "Portuguese (citizen)"/	1727
241	(portugal or portugues* or azores or acores or madeira or alentejo or algarve or lisboa or lisbon or 'alto tras-os-montes' or (ave adj3 (community or intermunicipal or comunidade)) or mondego or vouga or beira or cavado or lafoes or douro or porto or oporto or tejo or minho or setubal or pinhal or 'serra da estrela' or tamega or leira or santarem or beja or faro or evora or portalegre or 'castelo branco' or guarda or cimbra or aveiro or viseu or braganca or braganza or braga or 'vila real' or 'viana do castelo' or gaia or amadora or funchal or coimbra or almada or (agualva and cacem)).ab,in,ti.	222351
242	exp Romania/	11634
243	exp "Romanian (citizen)"/	499
244	(romania* or rumania* or roumania* or romani or rumani or alba or arad or arges or bacau or bihor or 'bistrita nasaud' or botosani or braila or brasov or kronstadt or brasso or brassovia or coron or bucharest or bucuresti or buzau or calarasi or 'caras-severin' or cluj or klausenburg or kolozsvar or constanta or tomis or konstantia or kostence or covasna or dambovita or dolj or galati or galatz or galac or kalas or giurgiu or gorj or harghita or hunedoara or ialomita or iasi or jassy or lassy or ilfov or maramures or mehedinti or mures or neamt or (olt and (river or county or region or judetul or raul)) or prahova or salaj or 'satu mare' or sibiu or suceava or teleorman or timis or tulcea or valcea or vilcea or vaslui or vrancea or timisoara or temeswar or temeschburg or temeschwar or temesvar or temisvar or timisvar or temesva or craiova or ploiesti or ploesti or oradea or varad or varat).ab,in,ti.	90588
245	exp Slovakia/	5129
246	exp "Slovak (people)"/ or exp "Slovak (citizen)"/	169
247	(slovakia or slovak* or slovaci or slovenki or bratislav* or presporok or pressburg or pressburg or posonium or banskobystri* or 'banska bystrica' or neusohl or besztercebanya or kosic* or kaschau or kassa or nitrian* or nitra or neutra or nyitra or nyitria or trnav* or tyrnau or nagyszombat or tyrnavia or presov* or trencian* or trencin or trentschin or trencsen or zilina or sillein or zsolna or zylina or (martin and (city or svaty)) or turocszentmarton or poprad or deutschendorf or zvolen).ab,in,ti.	55180
248	exp Slovenia/	4691
249	exp "Slovenian (citizen)"/	203
250	exp "Slovene (people)"/	20
251	(slovenia* or slovenija or slovensk* or slovenci or slovene* or gorenjska or carniola or goriska or gorizia or jugovzhodna or koroska or carinthia or 'notranjsko kraska' or 'obalno kraska' or 'coastal karst' or osrednjeslovenska or podravska or drava or pomurska or mura or savinjska or savinja or spodnjeposavska or zasavska or 'central sava' or posavska or 'lower sava' or ljubljana or laibach or lubiana or maribor or 'marburg an der drau' or kranj or carnium or creina or chreina or krainbur or koper or capodistria or kopar or celje or 'novo mesto' or neustadtl or domzale or velenje or wollan or woellan or 'nova gorica' or kamnik).ab,in,ti.	45407
252	exp Spain/	87383
253	exp Spaniard/	1830
254	exp "Basque (people)"/	103
255	(((spain or espana or spanish or espanol* or spaniard* or andalucia or andalusia or aragon or arago or cantabria or canarias or 'canary islands' or (canaries and island*) or 'castile) and leon') or 'castilla y leon' or 'castile la mancha' or 'castilla la mancha' or	907889

	cataluna or catalonia or ceuta or madrid or melilla or navarra or navarre or valencia* or extremadura or galicia or balears or 'balearic islands' or 'balear islands' or baleares or 'la rioja' or 'pais vasco' or 'basque country' or 'baske region' or euskadi or asturias or coruna or alava or araba or albacete or alicante or alacant or almeria or avila or badajoz or badajos or barcelona or burgos or caceres or cadiz or castellon or castello or 'ciudad real' or cordoba or cuenca or eivissa or ibiza or formentera or 'el hierro' or fuerteventura or galiza or girona or gerona or 'gran canaria' or granada or guadalajara or guipuzcoa or gipuzkoa or huelva or huesca or jaen or 'la gomera' or 'la palma' or lanzarote or leon or lleida or lerida or lugo or malaga or mallorca or majorca or menorca or minorca or murcia or ourense or orense or palencia or pontevedra or salamanca or segovia or sevilla or seville or soria or tarragona or tenerife or teruel or toledo or valladolid or vizcaya or biscay or zamora or zaragoza or saragossa or 'las palmas' or bilbao or bilbo).ab,in,ti.	
256	exp Sweden/	70763
257	exp Swedish citizen/	1633
258	exp "Swede (people)"/	167
259	(sweden or sverige or swedish or svenska or svenskar or swede or swedes or norrland or mellansverige or smaland or stockholm* or sydsverige or vastsverige or blekinge or dalarna or gavleborg* or gotland* or halland* or jamtland* or jonkoping* or kalmar or kronoberg* or norrbotten* or orebro or ostergotland* or skane or sodermanlands or uppsala or varmland* or vasterbotten* or vasternorrland* or vastmanland* or vastergotland* or gotaland* or gothenburg or goteborg or malmo or vasteras or linkoping or helsingborg or halsingborg or norrkoping).ab,in,ti.	503472
260	exp United Kingdom/	384393
261	exp British citizen/	1359
262	('gb' or 'united kingdom' or 'uk' or britain or british or england or english or scotland or scottish or scots or wales or cymru or welsh or 'north ireland' or 'northern ireland' or irish or avon or bedfordshire or berkshire or bristol or buckinghamshire or cambridgeshire or 'isle of ely' or cheshire or cleveland or cornwall or cumberland or cumbria or derbyshire or devon or dorset or durham or essex or gloucestershire or hampshire or southampton or (hereford and worcester) or hertfordshire or herefordshire or humberside or huntingdon or huntingdonshire or 'isle of wight' or kent or lancashire or leicestershire or lincolnshire or london or manchester or merseyside or middlesex or norfolk or northamptonshire or northumberland or nottinghamshire or oxfordshire or peterborough or rutland or shropshire or salop or somerset or yorkshire or staffordshire or suffolk or surrey or sussex or (tyne and wear) or warwickshire or midlands or westmorland or wiltshire or worcestershire or 'isle of man' or jersey or guernsey or 'channel islands' or aberdeen or aberdeenshire or angus or forfarshire or argyll or ayrshire or banffshire or berwickshire or bute or caithness or clackmannanshire or cromartyshire or dumfriesshire or dunbartonshire or dumbarton or dundee or lothian or haddingtonshire or edinburgh or fife or glasgow or 'inverness shire' or kincardineshire or 'kinross shire' or kirkcudbrightshire or lanarkshire or midlothian or moray or elginshire or nairnshire or orkney or peeblesshire or perthshire or renfrewshire or (ross and cromarty) or 'ross shire' or roxburghshire or selkirkshire or shetland or zetland or stirlingshire or sutherland or linlithgowshire or wigtownshire or anglesey or brecknockshire or caernarfonshire or carmarthenshire or cardiganshire or ceredigion or clwyd or denbighshire or dyfed or flintshire or glamorgan or gwent or gwynedd or merionethshire or montgomeryshire or monmouthshire or pembrokeshire or powys or radnorshire or antrim or aontroim or 'contae aontroma' or anthrim or antrim or entrim or armagh or 'ard mhacha' or airmagh or belfast or (down and (district or council or county)) or 'an dun' or 'an duin' or doon or doun or fermanagh or 'fear manach' or 'fhear manach' or fermanay or londonderry or doire or dhoire or lunnonderrie or derry or birmingham or leeds or sheffield or bradford or liverpool).ab,in,ti.	3558263
263	exp Liechtenstein/	112

264	(liechtenstein or lienchtensteiner* or balzers or eschen or gamprin or mauren or planken or ruggell or schaan or schellenberg or triesen or triesenberg or vaduz).ab,in,ti.	3694
265	exp Norway/	38549
266	exp "Norwegian (people)"/ or exp "Norwegian (citizen)"/	1307
267	(norway or norwegian* or norge or noreg or norgga or akershus or 'aust agder' or buskerud or finnmark or hedmark or hordaland or 'more og romsdal' or (more and romsdal) or 'more romsdal' or nordland or trondelag or oppland or oslo or ostfold or rogaland or 'sogn og fjordane' or (sogn and Fjordane) or 'sogn fjordane' or telemark or troms or romsa or romssa or 'vest agder' or vestfold or bergen or stavanger or sandnes or trondheim or trondhjem or kaupangen or nidaros or drammen or fredrikstad or skien or tromso or sarpsborg).ab,in,ti.	204437
268	exp Switzerland/	35145
269	exp Swiss/	2594
270	(switzerland or schweiz or schweizerische or swiss or suisse* or svizzera or svizzeri or svizzers or svizra or helvetica or aargau or argovia or ausserrhoden or 'outer rhodes' or innerrhoden or 'inner rhodes' or basel or bale or basilea or bern or berne or berna or fribourg or freiburg or friburg or geneva or geneve or genf or ginevra or genevra or glarus or graubunden or graubuenden or grisons or grigioni or grischun or jura or lucerne or luzern or neuchatel or nidwalden or nidwald or obwalden or obwald or schaffhausen or schaffhouse or schwyz or solothurn or soleure or thurgau or thurgovia or ticino or tessin or uri or valais or wallis or vaud or zug or zurich or zuerich or zurigo or lausanne or losanna or winterthur or winterthour or 'st gallen' or 'saint gallen' or 'sankt gallen' or 'saint gall' or 'san gallo' or 'son gagl' or turitg).ab,in,ti.	717154
271	exp Iceland/	5403
272	exp Icelander/	117
273	(iceland or icelandic* or islenska* or icelander* or islendinga* or islendigar or inslenska or reykjavik or reykjavikurborg or hofudborgarsvaedi or sudurnes or vesturland or vestfirdir or westfjords or nordurland or austurland or sudurland or kopavogur or hafnarfjordur or akureyri or gardabaer or mosfellsbaer or keflavik or akranes or selfoss or seltjarnarnes).ab,in,ti.	15187
274	151 or 152 or 153 or 154 or 155 or 156 or 157 or 158 or 159 or 160 or 161 or 162 or 163 or 164 or 165 or 166 or 167 or 168 or 169 or 170 or 171 or 172 or 173 or 174 or 175 or 176 or 177 or 178 or 179 or 180 or 181 or 182 or 183 or 184 or 185 or 186 or 187 or 188 or 189 or 190 or 191 or 192 or 193 or 194 or 195 or 196 or 197 or 198 or 199 or 200 or 201 or 202 or 203 or 204 or 205 or 206 or 207 or 208 or 209 or 210 or 211 or 212 or 213 or 214 or 215 or 216 or 217 or 218 or 219 or 220 or 221 or 222 or 223 or 224 or 225 or 226 or 227 or 228 or 229 or 230 or 231 or 232 or 233 or 234 or 235 or 236 or 237 or 238 or 239 or 240 or 241 or 242 or 243 or 244 or 245 or 246 or 247 or 248 or 249 or 250 or 251 or 252 or 253 or 254 or 255 or 256 or 257 or 258 or 259 or 260 or 261 or 262 or 263 or 264 or 265 or 266 or 267 or 268 or 269 or 270 or 271 or 272 or 273	12710169
275	25 and 150 and 274	24055
276	limit 275 to (conference abstract or conference paper or editorial or letter or note)	6617
277	275 not 276	17438
278	limit 277 to yr="2009 -Current"	8832

Supplementary Table S1c. Search string – Cochrane Library

Search	Terms	Results
#1	MeSH descriptor: [Prevalence] explode all trees	4656
#2	MeSH descriptor: [Population Surveillance] explode all trees	625
#3	MeSH descriptor: [Seroepidemiologic Studies] explode all trees	117
#4	MeSH descriptor: [Epidemiological Monitoring] explode all trees	29
#5	(seroepidemiolog* OR epidemiolog* OR surveillance* OR serolog* OR serosurvey* OR seroprevalen* OR seropositiv* OR (sero NEXT/2 (survey* OR epidemiolog* OR prevalen* OR positiv*))):ti,ab,kw (Word variations have been searched)	60204
#6	#1 OR #2 OR #3 OR #4 OR #5	60702
#7	MeSH descriptor: [HIV Infections] this term only	8989
#8	MeSH descriptor: [HIV] explode all trees	2896
#9	(hiv OR (human NEXT/3 (immune* OR immune*) NEXT/3 virus*)):ti,ab,kw (Word variations have been searched)	19254
#10	#7 OR #8 OR #9	19254
#11	#6 AND #10	3283
#12	MeSH descriptor: [HIV Seroprevalence] explode all trees	19
#13	MeSH descriptor: [HIV Seropositivity] explode all trees	702
#14	(((prevalen* OR seroprevalen* OR "sero prevalent" OR "sero prevalents" OR "sero prevalence" OR "sero prevalences" OR serodiagnos* OR serolog* OR diagnos* OR screen*) NEAR/10 hiv) OR ((prevalen* OR seroprevalen* OR "sero prevalent" OR "Sero prevalents" OR "sero prevalence" OR "sero prevalences" OR serodiagnos* OR serolog* OR diagnos* OR screen*) NEAR/10 human NEXT/3 (immune* OR immuno*) NEXT/3 virus*)):ti,ab,kw (Word variations have been searched)	5208
#15	#12 OR #13 OR #14	5700
#16	#11 OR #15	7203
#17	MeSH descriptor: [Emigrants and Immigrants] explode all trees	148
#18	MeSH descriptor: [Transients and Migrants] explode all trees	64
#19	(emigrant* OR immigrant* OR migrant* OR refugee* OR alien* OR migrat* OR emigrat* OR immigrat* OR minorit* OR foreigner* OR minority OR minorities OR origin* OR nationality OR (foreign* NEAR/2 born*) OR resettlement* displaced* OR asylum*:ti,ab,kw (Word variations have been searched)	21094

#20	#17 OR #18 OR #19	21094
#21	MeSH descriptor: [Prisoners] explode all trees	286
#22	MeSH descriptor: [Prisons] explode all trees	107
#23	(incarcerat* OR inmate* OR prisoner* OR confinement OR gaol* OR jail* OR penitentiar* OR prison* OR reformator* OR custodial OR custody OR custodies OR remand* OR detention* OR detainee* OR imprison* OR cellmate* OR convict* OR offender OR offenders OR ((correctional* OR penal OR internment OR closed) NEXT/5 (facilit* OR institution* OR camp* OR setting*))):ti,ab,kw	4522
#24	#21 OR #22 OR #23	4523
#25	MeSH descriptor: [Bisexuality] explode all trees	49
#26	MeSH descriptor: [Homosexuality, Male] explode all trees	293
#27	MeSH descriptor: [Sexual and Gender Minorities] explode all trees	39
#28	msm OR masm OR msmw OR gbmsm OR gb-msm OR cruisin* OR queer* OR ((men OR man OR male OR males) NEXT/3 (who OR has OR have OR having OR had OR report* OR inform*) NEXT/3 sex* NEXT/3 (men OR man OR male OR males)) OR ('male to male' NEAR/3 sex* NEAR/3 contact) OR ((gay* OR homosexual* OR bisexual* OR "non heterosexual" OR "non heterosexuals" OR "non binary" OR lesbigay* OR lbg) NEAR/5 (men OR man OR male OR males))	1507
#29	#25 OR #26 OR #27 OR #28	1527
#30	MeSH descriptor: [Transgender Persons] explode all trees	19
#31	MeSH descriptor: [Transsexualism] explode all trees	26
#32	(transgender* OR transpeople OR transperson* OR tran*sex* OR transm*n OR transwom?n OR "two spirit person" OR "two spirit persons" OR "two spirit individual" OR "two spirit individuals" OR "two spirit people" OR glbt OR glbtq OR glbtqq OR lgbt OR lgbtq OR lgbtqq OR (trans NEXT/2 (man OR men OR male OR males OR wom?n OR female* OR gender* OR person* OR people OR individual* OR sex*)) OR ((gender* OR sex*) NEXT/3 (variant* OR reassign* OR dissident*)) OR (intersex NEXT/3 (individual* OR people OR person*))):ti,ab,kw (Word variations have been searched)	8275
#33	#30 OR #31 OR #32	8275
#34	MeSH descriptor: [Sex Work] explode all trees	96
#35	MeSH descriptor: [Sex Workers] explode all trees	49
#36	((sex* NEAR/3 (transactional OR work* OR industr* OR exchange OR survival OR paid)) OR prostitut* OR fsw OR msw OR whore* OR "call girl" OR "call girls" OR streetwalker* OR nightwalker* OR "lad of the night" OR "lads of the night" OR "walk the pavement" OR "walk the pavements"):ti,ab,kw (Word variations have been searched)	771

#37	#34 OR #35 OR #36	771
#38	MeSH descriptor: [Gravidity] explode all trees	56
#39	MeSH descriptor: [Pregnancy] explode all trees	6925
#40	MeSH descriptor: [Pregnant Women] explode all trees	182
#41	MeSH descriptor: [Prenatal Care] explode all trees	1268
#42	MeSH descriptor: [Prenatal Diagnosis] explode all trees	749
#43	(pregnant* OR pregnanc* OR gravid* OR "child bearing" OR childbearing OR antenatal OR "ante natal"):ti,ab,kw	51627
#44	#38 OR #39 OR #40 OR #41 OR #42 OR #43	51816
#45	MeSH descriptor: [Substance Abuse, Intravenous] explode all trees	378
#46	(pwid OR idu OR ivdu OR<br>((inject* OR intraven* OR parenteral OR use OR misuse) NEAR/3 (drug* OR substance*))):ti,ab,kw	25441
#47	#45 OR #46	25552
#48	#20 OR #24 OR #29 OR #33 OR #37 OR #44 OR #47	107802
#49	#16 AND #48 with Cochrane Library publication date between Jan 2009 and Oct 2019	2184
	[Results in Cochrane Database of Systematic Reviews (CDSR)]	51

Supplementary Table S2. Inclusion and exclusion criteria

Inclusion criteria	Exclusion criteria
Articles published in 2009 or later reporting data from populations sampled in 2004 or later, including studies with data collection ending after 2004 (irrespective of start date).	Articles falling outside the specified sampling period or publication date range.
Articles reporting data from one or more EU/EEA Member States (see Table 4) and/or any of their regions/districts (overseas territories included).	Articles reporting data on non-EU/EEA countries only or not separating EU/EEA data out from non-EU/EEA country data.
Articles with the main purpose of assessing HIV prevalence in humans.	Articles not reporting data on HIV prevalence, or if virological markers tested for were not specified in the article.<br>Articles with the main aim of offering testing or assessing/evaluating a testing intervention but also reporting on positivity (will often be biased toward high-risk groups, previous positives will be excluded).<br>Articles reporting only self-reported HIV prevalence.
Articles reporting data from key population groups listed in Table 1.<br>Studies conducted in healthcare settings such as STI clinics, OST centres, gynaecologist etc.	Studies sampling only MSM practicing chemsex.<br>Studies of health care workers, military personnel or in/outpatients (patients in hospital settings and emergency departments, including specific diagnosis-related populations such as persons with hepatitis, tuberculosis, concurrent STIs and other indicator conditions, as well as blood donors
Peer-reviewed published literature.	Grey literature, including prevalence data published in national reports, conference abstracts or similar.<br>Modelling studies with no new measured prevalence estimates.<br>Articles reporting only data from a study not conducted in humans, environmental studies, technology assessments (studies on diagnostic and/or laboratory methods).<br>Opinion papers, editorials, guidelines or recommendations, perspectives, position papers, correspondence articles, systematic reviews or meta-analyses without original prevalence data.<br>Articles reporting data on pregnant women in countries with a population of >5 million inhabitants, but with a sample size <100 participants or with a sample size <50 participants for countries with a population of <5 million.<br>Articles reporting data on risk groups with a sample size of <100 participants. Sample sizes <100 and ≥50 are accepted for:<br>- Studies of transgender (TG) or groups with KP overlap<br>- Any risk group study from countries with a population of ≤1 million.
Studies reporting HIV prevalence data in individuals aged 15 years or older.	Studies specifically focused on children (aged 0-14 years), unless migrants.

Supplementary Table S3. Criteria for assessing the risk of bias in MSM/PWID/SW/TG and migrants

Population group	Data item	Criteria	Score
MSM/PWID/SW/TG	Sampling venue coverage	Single venue<br>Multi-centre/single venue type<br>Multi-centre/multiple venue types	0<br>1<br>2
	Sampling method	Convenience (non-random or non-exhaustive)<br>Exhaustive or random	0<br>1
	Sample size*	100-199<br>200+	0<br>1
Migrants	Age	Clear age bias (i.e. juvenile offenders only) or no information<br>No clear age bias in age profile of subjects	0<br>1
	Gender	Clear bias in gender or no information<br>No clear bias in gender distribution of subjects; could be considered representative if information is limited	0<br>1
	PWID	Exclusively among PWID/former PWID prisoners<br>PWID not used to select subjects; no bias toward PWID	0<br>1
	Population coverage	Single centre/local<br>Multi-centre/local or regional<br>Multi-centre/national	0<br>1<br>2
	Sampling method	Convenience (non-random or non-exhaustive)<br>Exhaustive or random	0<br>1
	Sample size	100-199<br>200+	0<br>1

MSM: men who have sex with men; PWID: people who inject drugs; SW: sex workers; TG: transgender people.

* For studies of transgender people, the sample size criteria for study inclusion was >50; studies with 50-100 participants were scored 0; studies with 100+ participants were scored 1.

Supplementary Table S4. Overview of included HIV seroprevalence studies with key results parameters, by population group and EU/EEA country

Population group	Country	Author (year)	Geographical coverage	N	Sampling method[a]	Sampling period	HIV prevalence (95% CI)	Total bias score[b]
Men who have sex with men	Belgium [29]	Vanden Berghe (2011)	Antwerp and Ghent	379	Time Location Sampling (TLS)	2009-2010	6.0% (4.9-9.1%)	4
	Belgium [24]	Mirandola (2018)	Brussels	582	TLS	2013-2014	12.3% (7.6-19.4%)	4
	Bulgaria [24]	Mirandola (2018)	Sofia	361	TLS	2013-2014	3.0% (0.9-9.1%)	4
	Croatia [25]	Kolaric (2011)	National (7 cities)	387	Convenience, NGO-based	2003-2006	3.0% (1.3-6.3%)	3
	Croatia [26]	Bozicevic (2012)	Zagreb	232	Respondent-driven sampling (RDS)	2010-2011	3.6% (1.1-5.1%)	3
	Cyprus [27]	Pylli (2014)	National	200	TLS	2011-2012	2.5% (0.08-5.7%)	3
	Czech Republic [28]	Mirandola (2009)	Prague	387	TLS	2008-2009	2.6% (1.0-4.2%)	4
	France [34]	Velter (2012)	Paris	2646	TLS	2009	17.7% (15.3-20.4%)	4
	France [35]	Sommen (2018)	National	886	TLS	2015	14.3% (12.0-16.9%)	4
	Germany [24]	Mirandola (2018)	Hamburg	390	TLS	2013-2014	7.5% (3.9-13.8%)	4
	Hungary [30]	Amirkhanian (2009)	Budapest	153	Convenience, community-based	2007	9.2% (5.1-14.9%)	2
	Italy [28]	Mirandola (2009)	Verona	390	TLS	2008-2009	11.8% (8.6-15.0%)	4
	Italy [24]	Mirandola (2018)	Verona	397	RDS	2013-2014	9.6% (4.5-14.9%)	4
	Lithuania [24]	Mirandola (2018)	Vilnius	322	RDS	2013-2014	3.4% (0.0-6.9%)	4
	Netherlands [38]	Achterbergh (2017)	Amsterdam	994	Exhaustive, STI-clinic based	2011	29% (26.3-32.0%)	1
	Poland [24]	Mirandola (2018)	Warsaw	405	TLS	2013-2014	7.2% (4.3-11.9%)	4
	Portugal [24]	Mirandola (2018)	Lisbon	371	TLS	2013-2014	17.1% (12.4-23.0%)	4
	Romania [28]	Mirandola (2009)	Bucharest	345	TLS	2008-2009	4.6% (2.4-6.8%)	4
	Romania [24]	Mirandola (2018)	Bucharest	181	RDS	2013-2014	18.0% (9.1-27.0%)	4
	Slovakia [28]	Mirandola (2009)	Bratislava	342	TLS	2008-2009	6.1% (3.5-8.6%)	4
	Slovakia [24]	Mirandola (2018)	Bratislava	400	RDS	2013-2014	4.3% (2.2-6.2%)	4

	Slovenia [23][c]	Klavs (2009)	Ljubljana	124<br>137	Convenience, NGO-based	2007<br>2008	2.4% (-)<br>2.2% (-)	0
	Slovenia [28]	Mirandola (2009)	Ljubljana	389	TLS	2008-2009	5.1% (2.9-7.3%)	4
	Slovenia [24]	Mirandola (2018)	Ljubljana	347	TLS	2013-2014	4.4% (2.1-8.9%)	4
	Spain [28]	Mirandola (2009)	Barcelona	388	TLS	2008-2009	17.0% (13.3-20.7)	4
	Spain [36]	Etcheverry (2010)	Madrid and Barcelona	299	Convenience, NGO-based	2005	4.7% (2.6-7.7%)	3
	Spain [37]	Folch (2010)	Catalonia	321<br>323	Convenience, community-based	2004<br>2006	24% (19.4-29.0%)<br>19.8% (15.6-24.6%)	3
	Spain [24]	Mirandola (2018)	Barcelona	400	TLS	2013-2014	14.2% (10.1-19.5%)	4
	Sweden [24]	Mirandola (2018)	Stockholm	356	TLS	2013-2014	2.4% (1.1-5.2%)	4
	United Kingdom [31]	McDaid (2011)	Glasgow and Edinburgh	1344<br>1228	TLS	2005<br>2008	4.4% (3.4-6.0%)<br>4.6% (3.5-6.0%)	4
	United Kingdom [32]	Wallace (2014)	Glasgow and Edinburgh	1218	TLS	2011	4.8% (3.8-6.2%)	3
	United Kingdom [33]	Aghaizu (2016)	London	1 377<br>1 503<br>1 153<br>1 106<br>965<br>782	Convenience, community-based	2004<br>2005<br>2006<br>2008<br>2011<br>2013	13% (-)<br>13% (-)<br>17% (-)<br>15% (-)<br>8% (-)<br>14% (11.2-16.2%)	3
	United Kingdom [24]	Mirandola (2018)	Brighton	402	TLS	2013-2014	17.6% (13.8-22.3%)	4
People who inject drugs	Croatia [25]	Kolaric (2011)	National (7 cities)	323	Convenience, NGOs and harm reduction sites	2003-2006	0.6% (0.0-2.2%)	3
	Croatia [39][d]	Kolaric (2010)	Split, Zagreb, Rijeka, national prison system	601<br>200	Convenience, harm reduction sites and prisons	2007	PWID 0.0% (-)<br>- PWID/prisoners 0.0% (-)	3
	Croatia [40]	Kolovrat (2010)	Brod-Posavina County	103	Exhaustive, drug treatment centres	2009	0.0% (-)	3
	Croatia [41]	Vilibić-Čavlek (2018)	National	153	Convenience, VCT, NGOs and harm reduction sites	2011-2013	0.7% (0.0-3.6%)	2
	Croatia [42]	Handanagic (2016)	Zagreb, Split, Rijeka	820	RDS	2014-2015	0.5% (0.1-1.2%)	4

	Estonia [64]	Uusküla (2011)	Tallinn	350<br>350<br>327	RDS	2005<br>2007<br>2009	54% (-)<br>55% (-)<br>50% (44.6-55.7%)	4
	Estonia [65]	Uusküla (2015)	Kohtla-Järve	370	RDS	2012	63% (59-67%)	4
	France [53]	Jauffret-Roustide (2009)	National	2389	TLS, harm reduction and community sites	2004-2005	10.8% (6.8-16.6%)	4
	France [54]	Weill-Barillet (2016)	National	1389	Random, harm reduction sites	2011	13% (10.2-17.2%)	4
	Germany [43][e]	Wenz (2016)	Eight cities	2077	RDS	2011-2014	Berlin 3.9% (-)<br>Cologne 6.1% (-)<br>Essen 0.0% (-)<br>Frankfurt 9.1% (-)<br>Hamburg 1.6% (-)<br>Hannover 8.7% (-)<br>Leipzig 3.0% (-)<br>Munich 5.0% (-)	4
	Germany [66][d,e]	Derks (2018)	Eight cities	1526	RDS	2011-2014	PWID/non-migrants 4.6% (-)<br>PWID/migrants 5.8% (-)	4
	Greece [55]	Hatzakis (2015)	Athens	3308	RDS	2012-2013	15.1% (13.9-16.4%)	4
	Greece [56]	Fotiou (2016)	Central and southern Greece	562	Exhaustive, OST sites	2013	15.7% (12.8-18.9%)	4
	Hungary [44]	Gyarmathy (2010)	Budapest	186	Convenience, NSP sites and outreach	2005-2006	0.0% (-)	2
	Italy [57]	Camoni (2009)	National	807	Random, drug treatment centres	2005	14.4% (11.7-17.4%)	3
	Lithuania [44]	Gyarmathy (2010)	Vilnius	297	Convenience, NSP sites and outreach	2008-2009	9.8% (6.6-13.7%)	2
	Luxembourg [45]	Removille (2011)	National	202	Convenience, drug treatment centres and prisons	2005	2.5% (0.3-4.6%)	3
	Poland [58]	Rosinska (2015)	National (6 regions)	763	Convenience, community, harm reduction and drug treatment sites	2005	18.0% (9.2-27.0%)	3
	Spain [59]	Huntington (2010)	Catalonia	296	Convenience, community-based	2006	58.1% (52.5-63.7%)	3

	Spain [60]	Sanvisens (2014)	Barcelona	1170	Exhaustive, OST sites	1992-2010	61.3% (58.4-64.1%)	3
	Spain [61][d]	Folch (2011)	Catalonia	748	Convenience, harm reduction sites	2008-2009	34.5% (31.1-38.0%)<br>- PWID/non-migrants 43.0% (-)<br>- PWID/migrants 22.4% (-)	2
	Spain [62][d]	Folch (2013)	Catalonia	810<br>159<br>-	Convenience, harm reduction sites	2008-2011	Men 31.5% (-)<br>Women 38.7% (-)<br>- PWID/FSW 53.3% (-)	3
	Spain [63]	Folch (2016)	Catalonia	761	Convenience, harm reduction sites and community	2010-2011	33.2% (29.8-36.5%)	3
	Sweden [46]	Blome (2011)	Malmö	1183	Exhaustive, NSP site	1997-2005	0.3% (0.0-0.9%)	2
	United Kingdom [47][d]	Marongiu (2012)	National	8671<br>317<br>8354	Exhaustive, harm reduction and drug treatment sites	1998-2007	0.9% (0.7-1.1%)<br>- PWID/MSM 3.2% (-)<br>- PWID/MSW 0.8% (-)	3
	United Kingdom [48]	Hickman (2009)	Bristol	299	RDS	2006	0.7% (0.1-2.4%)	3
	United Kingdom [49]	Hope (2014)	National	7149<br>6054	Exhaustive, harm reduction and drug treatment sites	2004-2007<br>2008-2011	1.4% (-)<br>1.4% (1.1-1.7%)	4
	United Kingdom [50]	Hope (2016)	National	383<br>228	Exhaustive, harm reduction and drug treatment sites (only men injecting image and performance enhancing drugs (IPEDs))	2010-2011<br>2012-2013	1.2% (-)<br>0.8% (0.1-3.1%)	4
	United Kingdom [51]	Hope (2017)	National	5261	Exhaustive, harm reduction and drug treatment sites	2011-2014	1.1% (0.8-1.4%)	4
	United Kingdom [52]	McAuley (2019)	Greater Glasgow and Clyde	927<br>865<br>915<br>821	Convenience, NSP sites	2012<br>2014<br>2016<br>2018	0.1% (0.0-0.6%)<br>1% (-)<br>3% (-)<br>5% (3.4-6.2%)	3
Prisoners	Croatia [68][d]	Burek (2010)	National	3348<br>815	Convenience	2005-2007	0.15% (0.1-0.4%)<br>- Prisoners/PWID 0.5% (-)	6
	Estonia [80]	Kivimets (2014)	National	3289	Exhaustive	2012	15.6% (14.5-17.1%)	6
	Finland [69]	Viitanen (2011)	National	88<br>295	Random	2006	Female 1.1% (0.0-6.2%)<br>Male 0.7% (0.1-2.4%)	7

	France [73][d]	Semaille (2013)	National	1351	Random	2010	2.0% (1.0-4.2%)<br>- Prisoners/non-migrants 1.1% (0.4-2.5%)<br>- Prisoners/migrants (SSA) 15.4% (6.6-31.8%)<br>- Prisoners/migrants (north Africa) 3.2% (0.4-24.6%)<br>- Prisoner/migrants (Americas) 3.5% (0.8–13.5%)<br>- Prisoners/migrants (Asia) 0.0%<br>- Prisoners/migrants (eastern Europe) 0.0%<br>- Prisoners/migrants (western Europe) 0.04% (0.005–0.4%)	7
	Germany [70]	Schulte (2009)	National	14537	Convenience	2006	1.2% (1.0-1.4%)	7
	Hungary [71]	Treso (2012)	National	4894	Exhaustive	2009	0.04% (0.0-0.15%)	6
	Hungary [72][d]	Vanya (2017)	Country's largest prison	200	Convenience	2014	Prisoners/MSM and/or PWID 0.0% (-)	2
	Italy [74]	Sagnelli (2012)	National	2339	Exhaustive	-	3.8% (3.1-4.7%)	5
	Italy [75]	Sinisgalli (2016)	Tuscany	3229	Exhaustive	2012	1.2% (0.9-1.6%)	6
	Italy [76]	Monarca (2015)	National	6630	Exhaustive	2013	5.1% (4.6-5.7%)	5
	Portugal [77]	Marques (2011)	Coimbra	151	Exhaustive	2008	6.6% (3.2-11.8%)	4
	Spain [67]	Hernandez-Fernandez (2010)	National	NA	Exhaustive	2004<br>2006<br>2008<br>2009	11.2% (-)<br>9.0% (-)<br>7.8% (-)<br>7.0% (-)	3
	Spain [78]	Ferrer-Castro (2012)	Ourense	425	Exhaustive (clinical records)	2009	8.5% (6.0-11.5%)	4
	Spain [79][d]	Marco (2012)	National	371<br>87<br>229<br>149	Random	2008	10.8% (7.5%-14%)<br>- Prisoners/PWID 39.0% (-)<br>- Prisoners/non-migrants 15.7% (-)<br>- Prisoners/migrants 2.7% (-)	6
Sex workers	Italy [84][d]	Zermiani (2012)	Verona	354<br>286	Convenience, outreach and STI clinic	1999-2007	FSW 4.6% (2.7-7.4%)<br>- FSW/Africa 4.9% (-)	2

			2<br>57			- FSW/South America 0.0% (-)<br>- FSW/Eastern Europe 3.5% (-)	
Netherlands [81][d]	van Veen (2010)	Rotterdam, Amsterdam, the Hague	547<br>69<br>88<br>390	Convenience, community-based	2002-2005	SW 5.7% (4.0-7.9%)<br>- FSW 1.5% (0.6-3.2%)<br>- SW/PWID 13.6% (7.6-22.0%)<br>- SW/TG 18.8% (10.9-29.4%)	3
Netherlands [82][d]	Fournet (2016)	National	2688<br>1881	Exhaustive, STI clinic	2006-2012	SW/male 2.5% (1.9-3%)<br>- SW/MSM 3.4% (-)	3
Netherlands [83]	Verscheijden (2015)	National	5770	Exhaustive, STI clinic	2013	0.1% (0.0-0.2%)	3
Portugal [87][d]	Dias (2015)	Lisbon	213<br>9<br>28<br>176	Convenience, community-based	2011	8.9% (5.5-13.6%)<br>- SW/TG 22.2% (0.0-56.0%)<br>- SW/male 10.7% (0.0-23.0%)<br>- SW/female 8.0% (4.0-12.0%)	2
Portugal [88][d]	Dias (2017)	National	176<br>88<br>44<br>44	Convenience, community-based	2011	8.0% (4.4-13.0%)<br>- FSW/non-migrant 8.0% (-)<br>- FSW/documented migrant 2.3% (-)<br>- FSW/undocumented migrant 13.6% (-)	2
Spain [36]	Etcheverry (2010)	Barcelona and Madrid	337	Convenience, NGOs	2005	4.2% (2.3-6.9%)	3
Spain [85][d]	Folch (2014)	Catalonia	400	Convenience, community-based	2005	1.8% (-)<br>- FSW/non-migrant 9.3% (-)<br>- FSW/migrant 0.8% (-)	3
			400		2007	2.5% (-)<br>- FSW/non-migrant 8.9% (-)<br>- FSW/migrant 1.5% (-)	
			400		2009	2.5% (-)<br>- FSW/non-migrant 11.4% (-)<br>- FSW/migrant 1.4% (-)	
			400		2011	1.5% (0.6-3.2%)<br>- FSW/non-migrant 14.7% (-)<br>- FSW/migrant 0.3% (-)	
United Kingdom [86][d]	Platt (2011)	London	268<br>105	Convenience, NGOs, clinic and community-based	2009	1.1% (0.1-2.4%)<br>- FSW/non-migrant 0.9% (-)	3

				163			- FSW/migrant 1.2% (-)	
Transgender people	Italy [89]	Luzzati (2016)	Trieste	20<br>173	Exhaustive, clinic-based	2000-2014	Female-to-male 0.0% (0.0-16.8%)<br>Male-to-female 12.1% (7.7-18.0%)	2

MSM: men who have sex with men; PWID: people who inject drugs; SW: sex workers; TG: transgender people; SSA: Sub-Saharan Africa.

[a] Sampling method: TLS = Time Location Sampling, RDS = Respondent Driven Sampling (both community-based).

[b] Bias score range: From 0 (high risk of bias) to 4 (low risk of bias) for MSM, PWID, SW and TG; and from 0 (high risk of bias) to 7 (low risk of bias) for prisoners. Detailed criteria for the risk of bias assessments are available in supplementary Table 3.

[c] Excluded from forest plot due to a high risk of bias (summary bias score = 0) and since other studies were available for MSM in Slovenia.

[d] Study including data for multiple (overlapping) risk groups.

[e] Both studies are based on the same data source; hence only Wenz [36] (original paper reporting on the full study population) was included in the forest plot while Derks [59] (later paper reporting on a sub-set of the original study population) is listed here because it reports prevalence data for a different population sub-group (migrant PWID versus non-migrant PWID).

Supplementary Table S5. Articles excluded after full-text review, grouped by reasons for exclusion

Reasons for exclusion	Number of excluded articles
Notification or incident data; or no HIV prevalence data; or data from evaluation of testing interventions; study protocol; clinical focus	79
Self-reported serological status; unclear confirmation of serological status	9
Duplicate reference/data	22
No original data (commentary, erratum, guidelines, etc.)	18
Review or overview article	50
Modelled data only	10
Sampling period outside defined criteria	9
Sample size too small	5
Did not address relevant key population group or did not distinguish drug injectors from drug users who did not inject	16
Study not conducted in the EU/EEA	14
Study not related to HIV	9
Sampled only persons with a hepatitis, tuberculosis or a concurrent STI	7
In-patient setting	7
Grey literature	4
TOTAL	259
